# Subfunctionalization of Paralog Transcription Factors Contributes to Regulation of Alkaloid Pathway Branch Choice in *Catharanthus roseus*

**DOI:** 10.3389/fpls.2021.687406

**Published:** 2021-05-25

**Authors:** Maite Colinas, Jacob Pollier, Dries Vaneechoutte, Deniz G. Malat, Fabian Schweizer, Liesbeth De Milde, Rebecca De Clercq, Joana G. Guedes, Teresa Martínez-Cortés, Francisco J. Molina-Hidalgo, Mariana Sottomayor, Klaas Vandepoele, Alain Goossens

**Affiliations:** ^1^Department of Plant Biotechnology and Bioinformatics, Ghent University, Ghent, Belgium; ^2^VIB Center for Plant Systems Biology, Ghent, Belgium; ^3^VIB Metabolomics Core, Ghent, Belgium; ^4^CIBIO/InBIO, Centro de Investigação em Biodiversidade e Recursos Genéticos, Universidade do Porto, Vairaão, Portugal; ^5^I3S-Instituto de Investigação e Inovação em Saúde, IBMC-Instituto de Biologia Molecular e Celular, Universidade do Porto, Porto, Portugal; ^6^ICBAS–Instituto de Ciências Biomédicas Abel Salazar, Universidade do Porto, Porto, Portugal; ^7^Faculdade de Ciências, Universidade do Porto, Porto, Portugal

**Keywords:** AP2/ERF transcription factor, bHLH transcription factor, *Catharanthus roseus*, Madagascar periwinkle, monoterpenoid indole alkaloids, transcription factor gene clusters, transcription factor paralogs, synergistic regulation

## Abstract

*Catharanthus roseus* produces a diverse range of specialized metabolites of the monoterpenoid indole alkaloid (MIA) class in a heavily branched pathway. Recent great progress in identification of MIA biosynthesis genes revealed that the different pathway branch genes are expressed in a highly cell type- and organ-specific and stress-dependent manner. This implies a complex control by specific transcription factors (TFs), only partly revealed today. We generated and mined a comprehensive compendium of publicly available *C. roseus* transcriptome data for MIA pathway branch-specific TFs. Functional analysis was performed through extensive comparative gene expression analysis and profiling of over 40 MIA metabolites in the *C. roseus* flower petal expression system. We identified additional members of the known BIS and ORCA regulators. Further detailed study of the ORCA TFs suggests subfunctionalization of ORCA paralogs in terms of target gene-specific regulation and synergistic activity with the central jasmonate response regulator MYC2. Moreover, we identified specific amino acid residues within the ORCA DNA-binding domains that contribute to the differential regulation of some MIA pathway branches. Our results advance our understanding of TF paralog specificity for which, despite the common occurrence of closely related paralogs in many species, comparative studies are scarce.

## Introduction

Plant specialized metabolites are chemically diverse, typically species- or taxa-specific and have often evolved in adaptation to the ecological niche of the plant. Within the plant, their production often exclusively takes place in specific organs or cell types and may be up-regulated precisely in response to defined environmental conditions [for a review, see Colinas and Goossens (2018)]. The integration of internal cues, such as cell type environment and developmental stage, and external cues, such as the presence of pathogens or herbivores, is predominantly regulated at the transcriptional level by specific transcription factors (TFs). Different TFs might act independently or cooperatively, forming regulatory modules that ensure the optimal spatiotemporal expression of specific metabolic pathway genes.

The medicinal plant *Catharanthus roseus* is well-known as being the only source of the important anti-cancer compounds vinblastine and vincristine (Van Der Heijden et al., 2004). These complex molecules belong to the class of monoterpenoid indole alkaloids (MIAs); around 150 different MIAs are estimated to occur in *C. roseus* (Van Der Heijden et al., 2004). The identification of many of the involved biosynthetic enzymes has revealed that genes involved in the different MIA pathway steps and branches are expressed in a cell type-specific, organ-specific and stress-dependent manner, making this species an ideal model to study how a modular system of different TFs possibly regulates specialized metabolism (Courdavault et al., 2014; Dugé De Bernonville et al., 2015).

**FIGURE 1:**
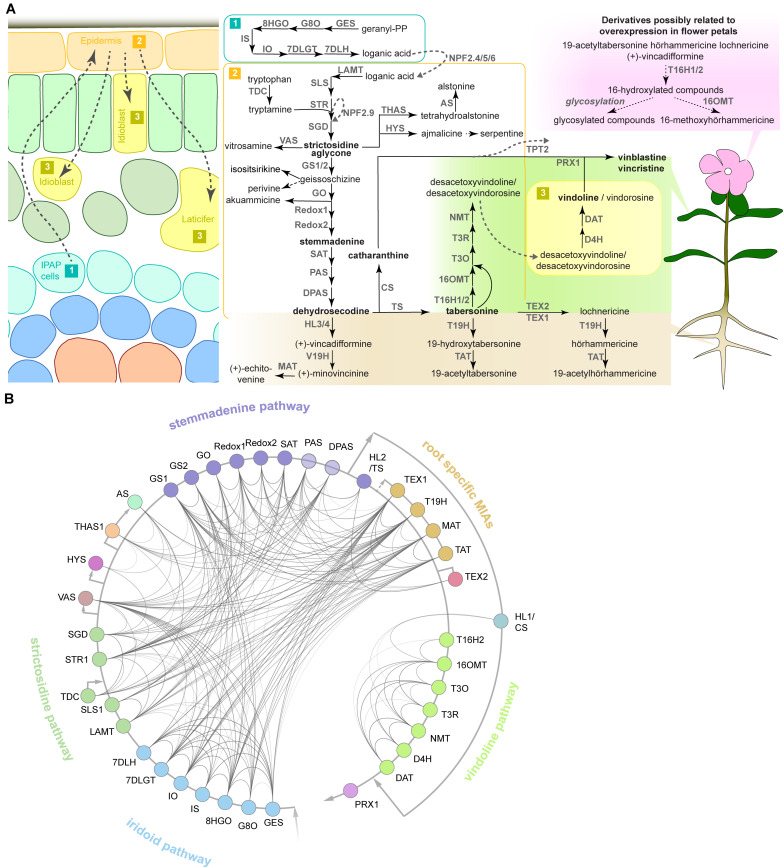
The MIA pathway consists of different branches with corresponding co-expression networks. **(A)** The MIA network and known or presumed cellular and organ-specific expression of the encoding MIA biosynthesis genes based on the current knowledge and available data. MIA biosynthesis is compartmentalized into the three different cell types shown on the left, although a cell type-specific expression has not been experimentally verified for all MIA biosynthesis genes such as the root MIA biosynthesis genes. Available organ-specific transcriptome data revealed that in particular two MIA branches are either root- or leaf-specific as shown on the right. In our study, transient overexpression of TFs in *C. roseus* flower petals is used to assess the impact on MIA biosynthesis at the gene expression level (by qPCR) and MIA metabolite level (by LC-MS analysis). Some MIA derivatives are detected in this flower petal expression system in this study, but it is not known where they occur in wild-type plants (upper right). Arrows indicate known characterized enzymatic reactions and corresponding enzymes; dashed black arrows indicate assumed enzymatic or chemical reactions for which no specific enzyme has been identified. Dashed gray arrows indicate transport and corresponding transporters. Note that NPF2.9 is an intracellular transporter conferring vacuolar export of strictosidine and TPT2 is an epidermis-specific transporter exporting catharanthine to the leaf surface. For clarity, only selected MIA metabolites are shown. **(B)** Co-expression network of MIA biosynthesis genes. From an expression atlas of 82 samples KNN networks were created. A line between genes indicates co-expression. Opacity of the lines is scaled according to the mutual rank of co-expression between two genes in their KNN 500 clusters (darker = stronger co-expression). To simplify the complex MIA biosynthesis network, we have colored the different pathway branches. Abbreviations: GES, geraniol synthase; G8O, geraniol-8-oxidase; 8HGO, 8-hydroxygeraniol oxidoreductase; IS, iridoid synthase; IO, iridoid oxidase; 7DLGT, 7-deoxyloganetic acid glucosyl transferase; 7DLH, 7-deoxyloganic acid hydroxylase; LAMT, loganic acid *O*-methyltransferase; SLS, secologanin synthase; TDC, tryptophan decarboxylase; STR, strictosidine synthase; NPF, nitrate/peptide family; SGD, strictosidine β-glucosidase; VAS, nitrosamine synthase; THAS, tetrahydroalstonine synthase; AS, a lstonine synthase: HYS, heteroyohimbine synthase; GS, geissoschizine synthase; GO, geissoschizine oxidase; SAT, stemmadenine-*O*-acetyl- transferase; PAS, precondylocarpine acetate synthase; DPAS, dihydroprecondylocarpine synthase; CS, catharanthine synthase; TPT, catharanthine transporter; T16H, tabersonine 16-hydroxylase; 16OMT, 16-hydroxytabersonine *O*-methyltransferase; T3O, tabersonine 3-oxygenase; T3R, tabersonine 3-reductase; NMT, 2,3-dihydro-3-hydroxytabersonine-N-methyltransferase; D4H, desacetoxyvindoline 4-hydroxylase; DAT, deacetylvindoline 4-*O*-acetyltransferase; PRX, peroxidase; HL, hydrolase; V19H, vincadifformine-19-hydroxylase; MAT, minovincinine-19-*O*-acetyltransferase; T19H, tabersonine 19-hydroxylase; TAT, 19-hydroxytabersonine 19-*O*-acetyltransferase; TEX, tabersonine epoxidase.

Different MIA pathway steps and branches can be organized into *subdivisions* with connected gene expression patterns (Figure 1). The first of such *subdivisions* is the *iridoid pathway*, i.e., the biosynthesis of the precursor loganic acid from geranyl diphosphate (GPP) via seven enzymatic steps occurring in the specialized internal phloem-associated parenchyma (IPAP) cells (Geu-Flores et al., 2012; Asada et al., 2013; Simkin et al., 2013; Miettinen et al., 2014; Salim et al., 2014). An iridoid intermediate, presumably loganic acid, is then transported to the epidermis, possibly by the recently characterized nitrate/peptide family (NPF) transporters (NPF2.4, NPF2.5, and NPF2.6) (Yamamoto et al., 2016, 2019; Larsen et al., 2017). The coupling of the then converted iridoid secologanin to the tryptophan-derived indole moiety tryptamine marks the formation of the first MIA strictosidine, from which, after deglycosylation, all MIAs derive (*strictosidine pathway*). A single enzymatic step is needed to convert the strictosidine aglycone either into the heterohymbines ajmalicine or tetrahydroalstonine (and subsequently alstonine) (Stavrinides et al., 2016; Dang et al., 2018), or into nitrosamine (Stavrinides et al., 2018). The branch leading to the precursors of the anti-cancer compounds has recently been resolved and involves seven enzymatic steps to yield the unstable intermediate dehydrosecodine (named *stemmadenine pathway* according to the name of one of the intermediates) (Tatsis et al., 2017; Caputi et al., 2018; Qu et al., 2018, 2019). From the latter either tabersonine or catharanthine are formed by two different enzymes (Caputi et al., 2018; Qu et al., 2018). Catharanthine forms one moiety of the MIA dimers vinblastine and vincristine and has been additionally suggested to have a repellent function as a monomer after secretion to the leaf surface by an epidermal ATP-binding cassette (ABC) transporter (Roepke et al., 2010; Yu and De Luca, 2013). The second monomer, vindoline, is produced from tabersonine via seven enzymatic steps, from which the two last steps are thought to take place in specialized idioblasts or laticifers (St-Pierre and De Luca, 1995; St-Pierre et al., 1999; Levac et al., 2008; Liscombe et al., 2010; Guirimand et al., 2011; Liscombe and O’connor, 2011; Besseau et al., 2013; Qu et al., 2015). Noteworthy, the *vindoline pathway* exclusively occurs in the green aerial parts of the plant (Liu et al., 2019). By contrast, in roots (particularly hairy roots), a different set of interconvertible *root-specific MIAs* (lochnericine, hörhammericine, minovincinine among others) is formed (Laflamme et al., 2001; Giddings et al., 2011; Carqueijeiro et al., 2018a, b; Williams et al., 2019). Finally, the assumed spatially separated monomers vindoline and catharanthine are dimerized to α-3′,4′-anhydrovinblastine, the direct precursor of vinblastine and vincristine, presumably by a peroxidase (Costa et al., 2008). Noteworthy, a very recent study suggests that catharanthine, as well as ajmalicine and its oxidation product serpentine, may also accumulate in idioblasts and laticifers in addition to the epidermis (Yamamoto et al., 2019). Thus, more research is needed in order to clarify the hypothesis of spatial separation of the monomers and differences in the sites of biosynthesis and accumulation of MIA monomers.

There has been a substantial increase in knowledge about which specific TFs from different families control the different MIA pathway steps and branches, although not complete yet. The main focus has been on the jasmonate (JA)-dependent transcriptional activation, because many (but not all, see below) MIA pathway steps are up-regulated upon exposure to this phytohormone that mainly mediates defense against necrotrophic pathogens and herbivores (Aerts et al., 1994; Zhang et al., 2011; De Geyter et al., 2012; Goossens et al., 2016, 2017; Zhou and Memelink, 2016). The primary response regulator of JA-dependent up-regulation of target genes is the basic helix-loop-helix (bHLH) TF MYC2, whose activity is post-translationally suppressed in the absence of JA (Chini et al., 2007; Goossens et al., 2017; Wasternack and Strnad, 2019). Recently, we have described how overexpression of the de-repressed *CrMYC2a*^*D126N*^ variant can boost the expression of the JA-responsive MIA biosynthesis genes (Schweizer et al., 2018). This up-regulation may partly occur directly and/or via the up-regulation of branch-specific TFs described below (Schweizer et al., 2018). For instance the regulators clade IVa bHLH iridoid synthesis (BIS) 1 and 2 TFs exclusively up-regulate the *iridoid pathway* genes (Van Moerkercke et al., 2015; Van Moerkercke et al., 2016). The expression of *strictosidine pathway* genes is controlled by members of the octadecanoid derivative-responsive *catharanthus* apetala2-domain (ORCA) clade (Menke et al., 1999; Van Der Fits and Memelink, 2000;Van Der Fits and Memelink, 2001; Paul et al., 2016; Paul et al., 2020; Singh et al., 2020). In addition, overexpression of ORCA3 has been shown to increase the expression of the *iridoid pathway* genes to some extent, part of the *stemmadenine pathway* genes and, when overexpressed in combination with *CrMYC2a*^*D126N*^, *root specific MIA* genes in a highly synergistic manner (Schweizer et al., 2018). While there is some knowledge about the target gene profiles of the other ORCAs (Paul et al., 2016, 2020; Singh et al., 2020), a direct comparison of them under the same experimental condition is currently lacking. Moreover, it has not been clarified, which, if any, of the ORCAs, in addition to ORCA3, act synergistically with CrMYC2a^*D126N*^. Noteworthy, the *vindoline pathway* does not appear to be up-regulated by JA and seems to be rather repressed upon overexpression of *CrMYC2a*^*D126N*^ (Schweizer et al., 2018). Very recently it has been shown that, the *vindoline pathway* is at least in part up-regulated by a GATA TF and down-regulated by phytochrome-interacting factor TFs in a light-dependent manner (Liu et al., 2019). However, the moderate changes in expression of the vindoline pathway genes and metabolite levels induced by these TFs suggest the presence of additional regulators. Other TFs, with a more limited impact on MIA biosynthesis have been described [for a review, see Liu et al. (2017)].

Together, the increasing identification of MIA biosynthesis genes over the recent years revealed a network of pathway branches (*subdivisions*) with differential gene expression profiles. This suggests the existence of specific regulatory factors acting independently or cooperatively in a branch-specific manner. Here, we sought out for regulators for pathway branches for which regulation was unclear or unknown by using co-expression analysis of publicly available and hitherto unpublished in-house generated RNA-Seq datasets. We identified additional members of well-known BIS, ORCA, and MYB clades. Our results further suggest that subfunctionalization of the ORCA TFs leads to differential regulation of particular subsets of MIA pathway genes. Likewise, synergistic up-regulation of MIA pathway genes in combination with de-repressed CrMYC2a appears to be paralog dependent.

## Materials and Methods

### Compilation and Generation of RNA-Seq Datasets

In total, 82 RNA-Seq samples were mapped to the latest *C. roseus* draft genome version (Kellner et al., 2015)^1^ and compiled in our compendium. In addition to publicly available datasets, RNA-Seq data from additional in-house experiments were included (see Supplementary Table 1). Two datasets consist of cell-type enriched transcriptomes obtained from macro-dissected stem (peeled) and leaf tissue (central vein compared to rest of leaf) as described previously (Van Moerkercke et al., 2015). A third dataset consists of RNA-Seq data from flower petals infiltrated with *Agrobacterium tumefaciens* C58C1 or infiltration buffer as control. The fourth dataset consists of transcriptomes of stably transformed hairy root lines constitutively overexpressing *BIS1*, *ORCA3* or the *green fluorescent protein* (*GFP*) (Van Moerkercke et al., 2015). In all cases, RNA was extracted from three biological replicates, as described previously (Van Moerkercke et al., 2015) and described below, and sequenced (50 bp, single-end) with an Illumina HiSeq2500 by GATC Biotech^2^.

### RNA-Seq Data Processing

RNA-Seq data was obtained from eight publicly available and unpublished datasets, covering 81 samples listed in Supplementary Table 1. Raw RNA-Seq data was processed with Prose from the Curse toolset (Vaneechoutte and Vandepoele, 2019). Prose uses FastQC (v0.11.7) (Andrews, 2012) and Trimmomatic (v0.38) (Bolger et al., 2014) to perform adapter clipping and quality trimming, and Kallisto (v0.44.0) (Bray et al., 2016) to quantify gene and transcript expression levels in Transcript Per Million (TPM) normalized values. For adapter clipping, Trimmomatic was run with the sliding window size set to 4 and the seed mismatches set to 2. Adapters were detected by FastQC and clipped in each sample separately. For quality trimming, Trimmomatic was run with a simple clip threshold of 10 and a minimum required quality of 15. Reads shorter than 32 bp after quality control were discarded. For Kallisto, an index of the *C. roseus* transcripts was created with a k-mer size of 31. The wrongly split transcript sequence for the *SGD* gene was manually corrected by merging the two CRO_T111319 and CRO_T111320. To process single-end reads with Kallisto, the mean fragment length was set to 200 and the standard deviation to 20. The *C. roseus* expression atlas is available in Supplementary Dataset 1. Prose was used to generate k-nearest neighbor (KNN) networks from the expression atlas of 82 samples. In this network, each gene was connected to its top 500 most co-expressing genes. This co-expression information was used for network and heatmap visualization.

### Candidate Selection

**FIGURE 2:**
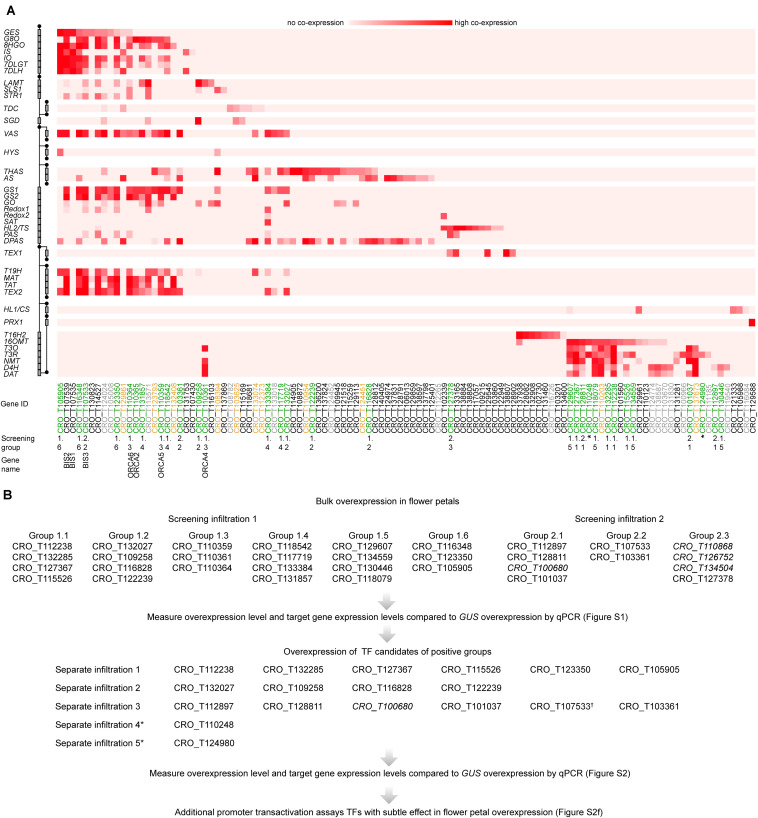
MIA regulator candidate selection and screening procedure. **(A)** Pathway genes (left) are plotted against regulator candidates (below); the relative rank of a regulator in the top 500 most co-expressed genes is depicted in red, with dark red indicating low ranks and strong associations between regulator and pathway gene. TF gene IDs are color-coded, indicating which genes by manual sequence analyses are likely not specific TFs and were thus not further considered (gray), which were considered for screening but cloning failed (yellow), and which were tested (green). Previously characterized TFs are labeled with their names in addition to “BIS3,” identified in this study. **(B)** Overview about the screening procedure. To enhance the output, TF candidates were bulk-overexpressed (“Screening groups”) by mixing *A. tumefaciens* strains containing the respective constructs. The screening was performed in two independent rounds of infiltration (“Screening infiltration 1 and 2”). In case of a detected up-regulation of target genes >2-fold and *P* < 0.05 (tested by Student’s *t*-test) (Supplementary Figure 1), the TF candidates from the respective group were overexpressed separately (Supplementary Figure 2). Gene IDs in italics were not picked in this co-expression analysis but in a previous one.

To search for potential regulators of gene expression, *C. roseus* transcripts were submitted to TRAPID (Van Bel et al., 2013). TRAPID was used to generate functional annotation for these transcripts based on sequence similarity and the presences of protein domains. In total, TRAPID annotated 1,586 genes with the Gene Ontology term “regulation of gene expression” (GO:0010468). Next, 44 genes with known involvement in the catharanthine and vindoline biosynthesis pathways were used as baits to select candidate regulators of these processes. For each bait gene, the top 500 most co-expressing genes in the KNN network were searched for the 1,586 genes that were predicted as regulators of gene expression by TRAPID. Genes that matched this selection were considered to be candidate regulators of the catharanthine or vindoline pathways, since they are both predicted regulators of gene expression and are co-expressed with known pathway genes. If more than 10 matches could be found for a single bait gene, only the top 10 most co-expressing candidates were kept. With 44 bait genes, this could result in at most 440 candidate genes, but due to overlap between the top 10 s of different baits, a final list of 111 candidates was selected (Figure 2A).

### Generation of DNA Constructs

Coding sequences (CDSs) of TFs were amplified using cDNA from *C. roseus* var. “Little bright eyes” or *C. roseus* var. “Sunstorm apricot” as templates. Amplification was performed using Q5^®^ High-Fidelity DNA Polymerase (New England BioLabs^®^) or iProof^TM^ (Bio-Rad) and gene-specific oligonucleotides containing attB Gateway^®^ recombination sites (Supplementary Table 2). Fragments were gel-purified (GeneJET Gel Extraction Kit, Thermo Fisher) and recombined into pDONR^TM^207 using Gateway^®^ BP clonase^TM^ II enzyme mix (Thermo Fisher), and the resulting clones sequence verified. ENTRY clones containing *CrMYC2a*^*D126N*^, *BIS1*, *BIS2* were cloned previously (Van Moerkercke et al., 2015; Van Moerkercke et al., 2016; Schweizer et al., 2018). ORCA3 protein mutants were constructed by site-directed mutagenesis using iProof^TM^ (Bio-Rad) and pDONR207_CrORCA3 as a template and complementary primers. Chimeric CrORCA3-4 and CrORCA4-3 were constructed by overlap extension PCR using chimeric primers. ENTRY clones containing TFs were recombined using LR Clonase^TM^ II enzyme mix (Thermo Fisher) into pH7WG2D or pK7WG2D for overexpression in flower petals, or p2GW7 for promoter transactivation assays in tobacco protoplasts (Karimi et al., 2002).

The promoter fragment of *STR* had been cloned previously (Vom Endt et al., 2007). The promoter fragments of *GO*, *MAT*, *TAT*, *T3O*, *T3R*, *D4H*, and *DAT* were amplified from *C. roseus* var. “Sunstorm apricot” genomic DNA using specific oligonucleotides (Supplementary Table 3), recombined into pDONR^TM^207 and sequence verified, as described for the TF CDSs. The resulting ENTRY clones containing promoter fragments were recombined into pGWL7 (Karimi et al., 2002) for transient expression assays in tobacco protoplasts.

### Transient Expression Assays in *Nicotiana tabacum* Protoplasts

Promoter transactivation of *C. roseus* gene promoters by *C. roseus* TFs was measured via transient expression assays in *Nicotiana tabacum* “Bright Yellow-2” protoplasts exactly as described previously (Vanden Bossche et al., 2013; Schweizer et al., 2018). Statistical analysis (ANOVA with Tukey’s test for multiple comparisons) was performed using PRISM GraphPad 8 software.

### Plant Material and Flower Petal Transformation

*Catharanthus roseus* var. “Little bright eyes” plants were grown in the greenhouse under 16-h/8-h light/dark conditions. Plants of the same age (6 months to 2 years) were used for flower petal infiltration with *A. tumefaciens* C58C1 harboring overexpression constructs as previously described (Schweizer et al., 2018) with minor adaptations. Briefly, all opened flowers were removed 2 days prior to infiltration. *A. tumefaciens* pre-cultures were inoculated from glycerol stocks 2 days prior to infiltration to inoculate final overnight cultures 1 day prior to infiltration. Cultures were washed and re-suspended as described (Schweizer et al., 2018). The suspensions were diluted and mixed (in case of combined overexpression of up to four TF overexpression constructs) to obtain a final concentration of OD_600_ = 0.4. Infiltration took place as described using four to five flowers, each from different individual plants. Samples were harvested in four biological replicates as described (Schweizer et al., 2018). In case of parallel analysis of gene expression and metabolite profiling, each sample was divided in two immediately before freezing.

### Gene Expression Analysis by qPCR

RNA extraction and reverse transcription were performed as described (Schweizer et al., 2018) with modifications. Up to 500 ng of RNA was used for reverse transcription using either the iScript^TM^ cDNA synthesis kit (Bio-Rad) or the qScript cDNA Synthesis Kit (Quantabio). qPCR was performed using a JANUS pipetting robot (PerkinElmer) and a LightCycler^®^ 480 (Roche) using SYBR Green qPCR master Mix (Agilent) and gene-specific oligonucleotides (Supplementary Table 5) in two technical replicates. The data was normalized to *N2227* and *SAND* as reference genes (Pollier et al., 2014) using qBase (Hellemans et al., 2007). Statistical analyses (ANOVA with Tukey’s test for multiple comparisons or Student’s *t*-test, as indicated) were performed on log2-transformed data using PRISM GraphPad 8 software. Principle component analyses (PCAs) of MIA target gene profiles were performed with ClustVis (Metsalu and Vilo, 2015).

### Metabolite Profiling

For metabolite extraction, around 100 mg of infiltrated flower petal samples were ground in liquid nitrogen, lyophilized and extracted with 1 ml methanol containing 100 μM caffeine as an internal standard. Extraction took place exactly as described previously (Schweizer et al., 2018). For LC-MS, 10 μL of the sample was injected in an Acquity UPLC BEH C18 column (150 × 2.1 mm, 1.7 μm; Waters, Milford, MA, United States) mounted on an Acquity UPLC system (Waters) connected to a Synapt HDMS qTOF-MS (Micromass) with an electrospray ionization source. A gradient was run at flow rate of 350 μL/min using acidified [0.1% (v/v) formic acid] solvents A [water/acetonitrile (99:1, v/v)] and B [acetonitrile/water; 99:1, v/v)]: time 0 min, 5% B; 30 min, 50% B; 33 min, 100% B. The mass spectrometer was set to positive ionization mode with the following parameter values: capillary voltage, 2.5 kV; sampling cone, 40 V; extraction cone, 4 V; source temperature, 120°C; desolvation temperature, 400°C; cone gas flow, 50 L/h; and desolvation gas flow, 750 L/h. Centroid data were recorded between *m*/*z* 100 and *m*/*z* 1,200 at a scan speed of 0.2 s/scan. Peak areas were determined using Progenesis QI software (Waters). PCAs were performed with MetaboAnalyst 4.0^3^ using Pareto-scaled mass spectrometry data with standard settings (Chong et al., 2018). Statistical analyses of identified MIAs (ANOVA with Tukey’s test for multiple comparisons) were performed using PRISM GraphPad 8 software. Identifications of the MIAs were done using FT-MS and MS*^*n*^* data generated as described (Schweizer et al., 2018) and are shown in Supplementary Dataset 2.

### Gene IDs and Data Deposition

Gene IDs (*C. roseus* genome version 2) are listed with the corresponding primer sequences in Supplementary Table 4. In some cases, the cloned sequences differed from publicly available genome and transcriptome data. All sequences cloned for this study have been deposited in the GenBank database; accession numbers are also listed in Supplementary Table 4.

## Results

### TF Candidate Selection and Screening Impact on MIA Biosynthesis

To enable comprehensive mining of *C. roseus* transcriptomes for novel MIA regulators, first a total of 82 RNA-Seq datasets were mapped to the latest *C. roseus* draft genome version (Kellner et al., 2015) (see text footnote 1). In addition to publicly available datasets, RNA-Seq data from hitherto unpublished in-house experiments were included, including data of cell type-enriched transcriptomes (see methods and Supplementary Table 1). Across these datasets, branches of MIA pathway genes (Figure 1B) showed a high level of co-expression. Noteworthy, in particular the *vindoline* branch and catharanthine biosynthesis were not co-expressed with other MIA pathway branches (Figure 1B). A KNN network was created and for each MIA biosynthesis gene the top 500 most co-expressing genes were searched for genes with the GO term “regulation of gene expression” (GO:0010468) (see “Materials and Methods” section for details) yielding a list of 111 candidates (Figure 2A). Manual sequence analysis of the candidates revealed that 15 were likely not unambiguously corresponding to genuine TFs and thus not considered for further analysis. Consistent with previous results, among the co-expressed candidates we found the previously characterized TFs BIS1 and 2 and ORCA2, 4, 5, and 6, but not ORCA3. We further selected those TFs that showed co-expression with (i) multiple pathway genes and (ii) MIA pathway genes for which till now no transcriptional regulators are known, in particular genes belonging to the *stemmadenine* or *vindoline* branches or involved in catharanthine biosynthesis, and that were not predicted to act as repressors. Out of the 40 selected candidates, 28 were successfully cloned comprising all but two candidates that were ranked as candidates of highest priority and subjected to agroinfiltration in *C. roseus* flower petals. Four additional candidates, that were not found through this co-expression analysis but through a preceding, preliminary, analysis, were also tested in this screening (gene IDs in italic font in Figure 2B). The latter preceding co-expression analysis was performed exactly as described above but on 72 RNA-Seq samples because one dataset (Pan et al., 2018) had not yet been available at the start of this project (data not shown). While the candidate lists of the two analyses greatly overlapped those four candidates were exclusively found in the first analysis. Thus altogether 32 candidates were tested by bulk overexpression in *C. roseus* flower petals as described hereafter.

To enhance the throughput, up to four TF candidates were co-overexpressed under the control of the *CaMV35S* promoter (p35S). We specifically combined those candidates that were co-expressed with the same (or overlapping) subsets of MIA pathway genes. This resulted in nine overexpression groups of candidates (Figure 2B). *A. tumefaciens* harboring a plasmid for *p35S:GUS* expression was infiltrated as a control. The overexpression level of the candidate TFs and the expression level of the MIA pathway genes were then measured by qPCR (Supplementary Figure 1). In case of successful overexpression (>5-fold) and observed increase(s) (>2-fold, *P*-value according to Student’s *t*-test < 0.05) in expression level(s) of potential target MIA pathway genes compared to the *p35S:GUS* control, the TF candidates from the respective group were overexpressed separately (Figure 2B; Supplementary Figure 2). In the case of CRO_T132285 and CRO_T127367, for which the increase in target gene expression level was at the threshold level, additional promoter transactivation assays were carried out that did not confirm a (direct) up-regulation of MIA target genes (Supplementary Figure 2F). Altogether the results from the screening suggested that only the ORCA TFs (CRO_T110359, CRO_T110361, and CRO_T110364) and clade IVa bHLH TFs (CRO_T123350, CRO_T105905, and CRO_T107533) reproducibly led to an increase in MIA biosynthesis gene expression levels (Supplementary Figures 1, 2 and below).

### The *Iridoid* Regulatory Module: Identification of an Additional *BIS* Paralog That Clusters With *BIS1* and *2*

**FIGURE 3:**
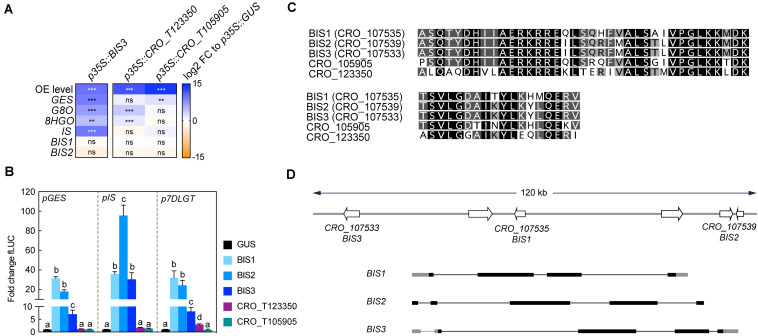
BIS3 up-regulates iridoid pathway genes. **(A)** Overexpression of *BIS3* leads to increased expression levels of iridoid pathway genes similar to those previously observed for *BIS1* and *2*, whereas overexpression of the other clade IVa TFs CRO_T123350 and CRO_T105905 leads to comparably much lower increases. *BIS1* and *2* themselves are not up-regulated by BIS3, suggesting that the iridoid pathway up-regulation is directly caused by BIS3 and that there is no cross up-regulation between the three BIS TFs. Asterisks indicate statistically significant differences in expression (**P < 0.01, ***P < 0.01) as calculated by Student’s *t*-test for each gene compared to GUS control (not included in this graph). **(B)** *N. tabacum* protoplasts were co-transfected with constructs containing the *firefly luciferase* gene (*fLUC*) expressed under control of the indicated promoter fragments and constructs for overexpression of *bHLHs* as indicated or *GUS* as a control. The *y*-axis shows fold change in normalized fLUC activity relative to the control transfection with GUS, set at 1. The error bars depict SEM of four biological replicates. For each promoter, columns labeled with different letters represent statistically significant differences (*P* < 0.05, ANOVA with Tukey’s correction for multiple comparisons). BIS1-3 redundantly highly transactivate iridoid pathway promoters whereas CRO_T123350 and CRO_T105905 have no effect. **(C)** A MUSCLE alignment of the bHLH domain protein sequences reveals a high level of sequence identity between the five clade IVa bHLHs. However, BIS1, 2 and 3 show a slightly higher level of identity than CRO_T123350 and CRO_T105905 potentially explaining the differential level of up-regulation of iridoid pathway genes. Sites that are identical in all five sequences are colored in black; sites identical in four out of five sequences are colored in dark gray and sites identical in 3 out of 5 sequences are colored in light gray. **(D)** The latest version of the *C. roseus* genome revealed that the previously published BIS1 and 2, and BIS3 identified in this study, are all located in close proximity and have similarly organized gene structures suggesting that they probably arose from recent gene duplications. Exons are depicted as bars with untranslated regions (UTRs) in gray and CDS’ in black. Introns are depicted as black lines.

Overexpression of the three newly identified iridoid pathway co-expressed (Figure 2) clade IVa bHLHs (CRO_T123350, CRO_T105905, and CRO_T107533) led to enhanced expression of the iridoid genes, albeit to very different levels (Figure 3A). Only overexpression of CRO_T107533 (referred to as *BIS3* hereafter) led to increases in *iridoid pathway* transcript levels (28- to 480-fold) similar to overexpression of *BIS1* and *2* in our previous study (Van Moerkercke et al., 2016). This high up-regulation of iridoid pathway genes by BIS1, 2, and 3 in comparison to the CRO_T123350 and CRO_T105905 was further confirmed by promoter transactivation assays in transient expression assays in *N. tabacum* (tobacco) protoplasts on (Figure 3B). We did not observe consistent differential or synergistic effects of combinations of the three BIS on iridoid pathway gene promoters (Supplementary Figure 3), suggesting that homo- or heterodimers of BIS1/2/3 have similar transactivation capacities and specificities. A ClustalW alignment of the highly conserved bHLH domains of the five clade IVa factors reveals a higher level of sequence identity between the three BIS (BIS2 and 3 bHLH sequences are identical) compared to CRO_T123350 and CRO_T105905 (Figure 3C). Noteworthy, all three *BIS* TFs cluster within a 120-kb locus in the genome and are probably the result of recent tandem gene duplication events (Figure 3D). This was previously not known but could now be detected because of the recent update (v2) of the *C. roseus* genome (see text footnote 1).

### The ORCA Cluster: Functional Diversification Into Branch-Specific Regulatory Modules

**FIGURE 4:**
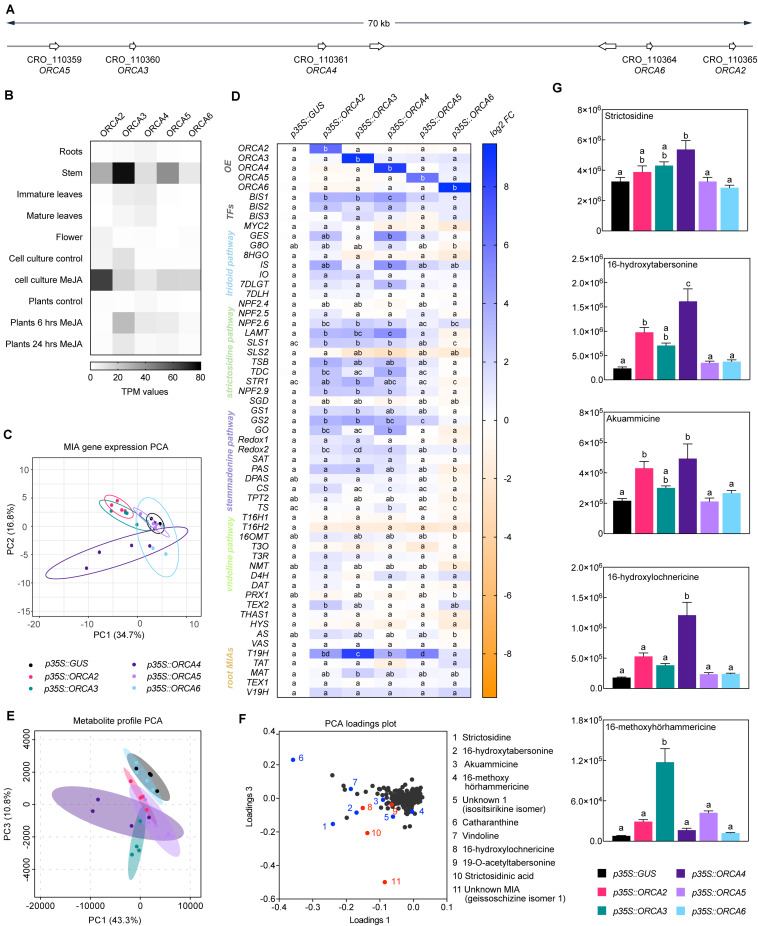
Members of the ORCA TF cluster differentially up-regulate MIA biosynthesis genes. **(A)** Localization of the *ORCA* genes within the cluster. **(B)** Expression pattern of *ORCA* paralogs extracted from publicly available RNA-Seq data from indicated tissues and conditions. TPM, Transcripts Per Kilobase Million. **(C)** A PCA of MIA pathway genes measured by qPCR upon *ORCA* overexpression [shown in panel **(D)**] suggests overlapping but also differential MIA target gene expression patterns in particular between *ORCA2*, *3*, and *4*. PCA was performed with ClustVis (Metsalu and Vilo, 2015). **(D)** Expression of MIA biosynthesis genes measured by qPCR upon *ORCA* overexpression shown as log2 fold changes (FC) compared to the overexpression of *GUS* as a control. For each pathway gene, cells labeled with different letters represent statistically significant differences (*P* < 0.05, ANOVA with Tukey’s correction for multiple comparisons). Note that the overexpression levels of *ORCA3*, *4*, and *6* were higher than those of *ORCA2* and *5*. **(E)** PCA of metabolite profiling data from *ORCA* overexpression samples. **(F)** Loading plot of the PCA shown in panel **(E)**, indicating that the differences found in the PCA are indeed predominantly caused by MIA metabolite levels. Selected known MIAs are labeled in blue; MIAs identified in this study (see Supporting Dataset S1) are labeled in red. **(G)** Levels of selected MIA metabolites expressed in average total ion current (TIC). The levels of all measured MIA metabolites can be found in the supporting information (Supplementary Figure 6; Supplementary Table 6). Columns labeled with different letters represent statistically significant differences (*P* < 0.05, ANOVA with Tukey’s correction for multiple comparisons). Error bars are standard error of the mean (SEM) of four biological replicates.

**FIGURE 5:**
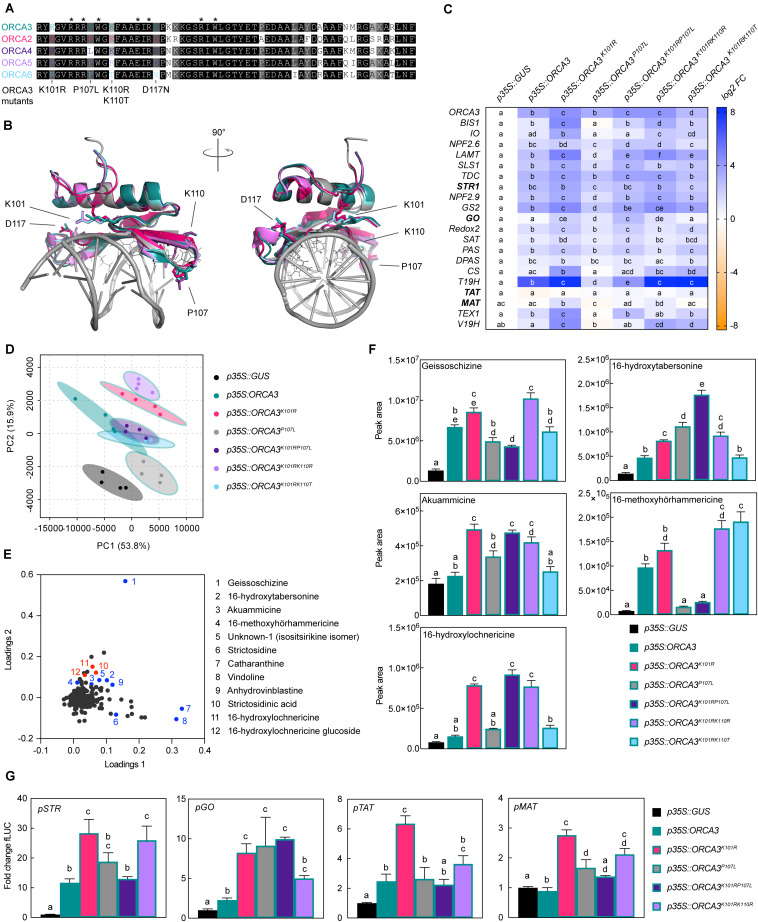
Specific amino acid residues (AARs) in the DNA-binding domain (DBD) of ORCA TFs influence target gene specificity. **(A)** Alignment of the DBDs of ORCAs. AARs labeled with an asterisk were identified previously as directly interacting with the DNA based on the solved crystal structure of the *Arabidopsis* AP2/ERF member AtERF1 DBD (Allen et al., 1998; Shoji et al., 2013). ORCA3 was used as a template to exchange AARs so that they would correspond to AARs in other ORCA TFs. The respective mutated AARs are indicated below the alignment. **(B)** Homology models of ORCA DBDs. The models [made by Phyre2 “intensive mode” (Kelley et al., 2015); colored as in panel **(A)**] are overlaid with the AtERF1 structure bound to DNA (PDB 1GCC) colored in gray. AARs identified as directly interacting with the DNA [asterisks in panel **(A)**] are shown as lines of AtERF1. AARs differing in the ORCA DBDs and selected for site-directed mutagenesis are shown as sticks for all ORCA models, while the AAR numbering refers to ORCA3. The models suggest that the differing AARs are unlikely to interact directly with the DNA, but are in close proximity to the DNA and might thereby modulate DNA binding affinity. **(C)** Overexpression of *ORCA3* mutants in *C. roseus* flower petals and expression level of selected MIA target genes as measured by qPCR. Two AAR changes, K101R and P107L, seem to significantly modulate the MIA target gene profile. While K101R appears to increase the level of up-regulation, up-regulation is greatly reduced and in some cases nearly abolished when overexpressing the *ORCA3*^*P107L*^ mutant. The double mutant *ORCA3*^*K101RP107L*^, corresponding to the native ORCA4 sequence, recovers some activation potential, and reaches similar levels than observed upon overexpression of *ORCA4* (Figure 4D). This suggests that the combination of these AARs may account for the differences between ORCA3 and 4. For each pathway gene, cells labeled with different letters represent statistically significant differences (*P* < 0.05, ANOVA with Tukey’s correction for multiple comparisons). Results from overexpression of all *ORCA3* single AAR mutants (including *ORCA3*^*D117N*^) can be found in Supplementary Figure 6. **(D)** PCA of metabolite profiling data. **(E)** Loading plot indicating that sample separation shown in the PCA plot is largely due to changes in MIA metabolite levels. Selected known MIAs are labeled in blue; MIAs identified in this study are labeled in red. **(F)** Levels of selected MIA metabolites expressed in average total ion current (TIC). The levels of all measured MIA metabolites can be found in the supporting information (Supplementary Figure 8; Supplementary Table 7). Columns labeled with different letters represent statistically significant differences (*P* < 0.05, ANOVA with Tukey’s correction for multiple comparisons). Error bars are standard error of the mean (SEM) of four biological replicates. **(G)** Transactivation assays of *pSTR*, *pGO*, *pTAT*, and *pMAT* performed in tobacco protoplasts. Columns labeled with different letters represent statistically significant differences (*P* < 0.05, ANOVA with Tukey’s correction for multiple comparisons). Error bars are standard error of the mean (SEM) of four biological replicates.

**FIGURE 6:**
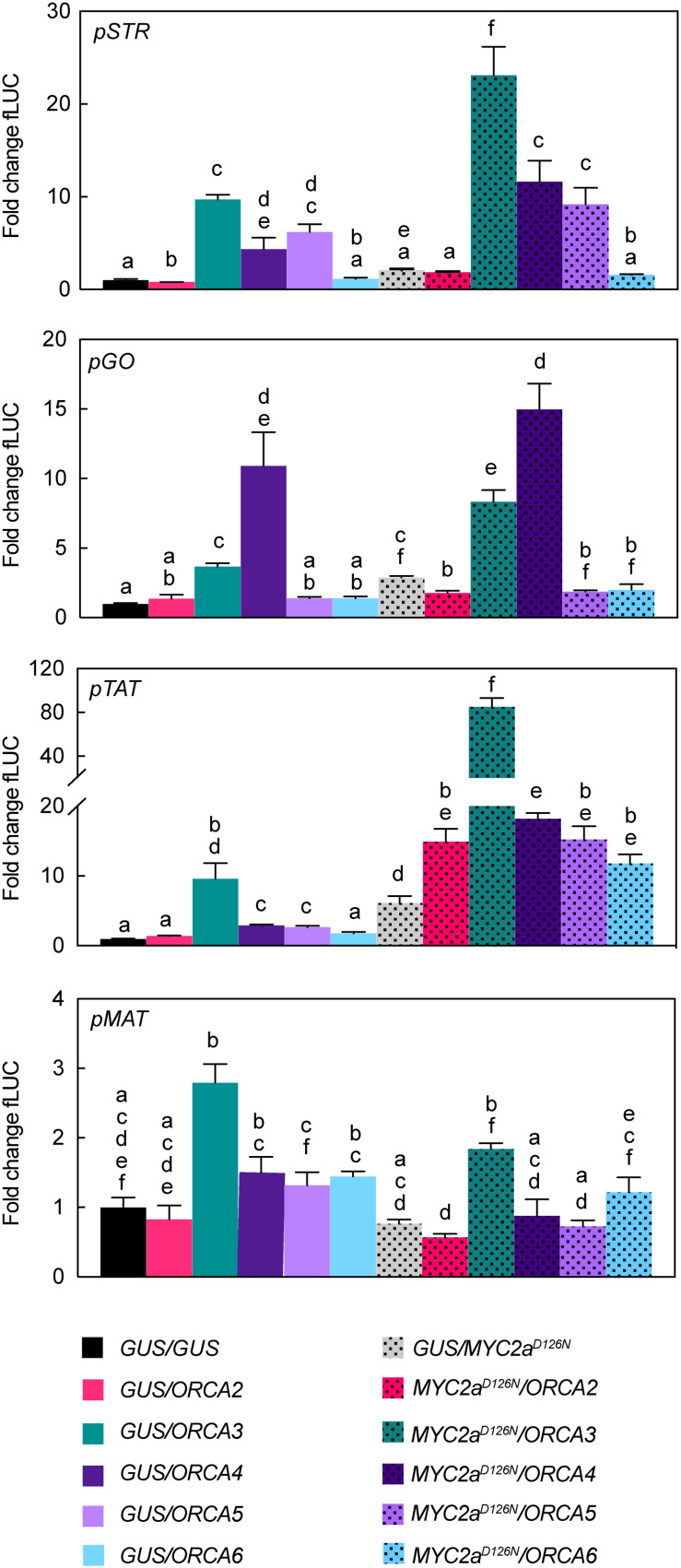
Transactivation of *STR*, *GO*, *TAT*, and *MAT* promoter fragments by ORCAs and CrMYC2a^*D126N*^. *N. tabacum* protoplasts were co-transfected with constructs containing the *fLUC* gene expressed under control of the indicated promoter fragments and constructs for overexpression of *ORCAs*, *CrMYC2a*^*D126N*^, or *GUS* as a control. Note in particular differential transactivation of *pGO* and *pTAT* upon *ORCA3* versus *ORCA4* expression and synergistic effects on *pTAT* upon combined overexpression of *ORCA3* and *CrMYC2a*^*D126N*^ in combination. The *y*-axis shows fold change in normalized fLUC activity relative to the control transfection with GUS, set at 1. The error bars depict SEM of four biological replicates. For each promoter, columns labeled with different letters represent statistically significant differences (*P* < 0.05, ANOVA with Tukey’s correction for multiple comparisons).

In addition to the long-known *ORCA2* and *3*, *ORCA4*, *5*, and *6* have recently been found to be members of a gene cluster together with *ORCA3*, and their target gene profile has partly been elucidated (Paul et al., 2016, 2020). All ORCA TFs together form a cluster spreading over 70 kb, which can be subdivided into an ORCA3, 4, and 5 subcluster (around 25 kb) and an ORCA2 and 6 subcluster (around 10 kb) (Figure 4A). A combination of different computational sequence analyses led us to hypothesize that the five ORCA TFs are possibly not fully functionally redundant. First, key amino acid residues (AARs) within the DNA-binding domain (DBD) differ between the ORCAs and are predicted to confer direct interaction with DNA differ (Figure 5A). Second, it is generally accepted that the activation domains (ADs) of TFs are mostly poorly conserved at sequence level, but it is rather the structural properties defining the level of activation (Staby et al., 2017). The combined information of different prediction programs suggested that the ORCA TFs differ significantly in their structural organization, further hinting toward a certain degree of diversification (Supplementary Figure 4). Third, ORCA3-5 contain a serine-rich C-terminal domain that ORCA2 and 6 lack. Fourth, the five ORCA TFs showed a differential expression profile (Figure 4B and Supplementary Figure 5) and our co-expression analysis revealed that they co-express with different subsets of MIA pathway genes (Figure 2A). Despite the occurrence of clusters of ORCA orthologs in other species, such as tomato and tobacco, it has not been systematically analyzed in any of these species if and to which level they are functionally redundant (Cárdenas et al., 2016; Kajikawa et al., 2017). Therefore, we sought out to decipher their specific regulatory roles by comparing their target gene and metabolite profile upon overexpression in flower petals. A PCA analysis of the MIA gene expression levels revealed that ORCA2-4 showed only partly overlapping target gene profiles (Figure 4C). Particularly the expression of early and intermediate MIA pathway genes increased to similar levels upon overexpression of ORCA2-4 (Figure 4D, for instance *SLS1*, *STR1*, *GS1*, *GS2*, *REDOX1*,*2*). However, in other cases, the fold changes differed considerably. For instance, *GO*, which encodes an enzyme producing the intermediate geissoschizine but also akuammicine (see Figure 1A), as well as the iridoid pathway genes including the regulator BIS1, were considerably more highly up-regulated upon overexpression of *ORCA4*, and to a lesser extent of *ORCA2* compared to overexpression of the other *ORCAs* (Figure 4D). Conversely, the *root MIA* genes *T19H* and *MAT* were more highly up-regulated when *ORCA3* was overexpressed. It should be noted that the overexpression level of the different *ORCA* paralogs differed considerably, with *ORCA3*, *4*, and *6* being highly overexpressed (around 510-, 440-, and 960-fold, respectively) and with *ORCA2* and in particular *ORCA5* somewhat less (93-, and 15-fold, respectively) overexpressed (Figure 4D). Thus, the overall lower MIA biosynthesis gene up-regulation levels observed for *ORCA5* overexpression might be partly due to the lower overexpression level of *ORCA5*. We did not observe any “intra-cluster” up-regulation between the different ORCA TFs. We next performed metabolite profiling on samples from this experiment (Figures 4F,G, Supplementary Figure 6 and Supplementary Table 6). A PCA analysis of the Mass spectrometry (MS) data revealed a (partially) differential profile (Figure 4E), which was caused by differential accumulation of MIA metabolites (Figure 4F). In addition to MIAs identified in a previous study (Schweizer et al., 2018), the loadings plot further showed differentially accumulating compounds that we identified as MIAs (Figure 4F (red), Supporting Dataset S2). In particular, akuammicine, 16-hydroxytabersonine and 16-hydroxylochnericine accumulation increased more when *ORCA4* was overexpressed and that of the root MIA derivative 16-methoxyhörhammericine when *ORCA3* was overexpressed (Figure 4G). Overexpression of *ORCA6* had only mild effects on MIA pathway gene expression and no noticeable effects on MIA abundance. Promoter transactivation assays in tobacco protoplasts with promoter fragments of *STR*, *GO*, *MAT*, and *TAT* expressed with and without the addition of CrMYC2a^*D126N*^ overall confirmed these observations (Figure 6). ORCA4 transactivated *pGO* to a much higher extent than the other ORCA TFs, whereas ORCA3 transactivated *pTAT* higher than other ORCAs, whereas *pSTR* was similarly transactivated by ORCA3, 4, and 5. Together, these data support both redundant and specific functions of the different ORCA TFs in the regulation of MIA biosynthesis.

### Specific AARs Within ORCA DBDs Contribute to Target Gene Specificity

Generally, the DBD is thought to define the specificity to the DNA motif, whereas the AD defines the level of up-regulation by recruiting the transcriptional machinery. In a previous study, the affinities of different orthologs of group IXa AP2/ERFs, i.e., ORCA3 and its orthologs from tobacco and *Arabidopsis thaliana*, to the DNA motif were compared (Shoji et al., 2013). The results suggested that the specific binding affinity to different DNA motifs is indeed mediated by specific AARs in the DBD. However, specific AARs mediating target gene specificity among paralogs from the same species have never been characterized in detail to our knowledge. A protein sequence alignment of the DBDs of the ORCAs shows the expected overall high level of sequence conservation (Figure 5A). Likewise, structural models of the ORCA DBDs are almost identical and highly align with the solved structure (PDB 1gcc) of the Arabidopsis ortholog AtERF1 (Allen et al., 1998; Figure 5B). While the paralog-specific AARs corresponding to K101, P107, K110, and D117 in ORCA3 are not predicted to directly interact with DNA in the shown model, they are nevertheless predicted to be part of the beta-sheets in close proximity to the DNA (Figure 5B). To compare a potential impact of these AARs on MIA target gene specificity, we performed site-directed mutagenesis on ORCA3 to obtain single (K101R, P107L, K110R, K110T, and D117N) and double (K101R/P107L, K101R/K110R_K101R/K110T) mutants, whose sequence would correspond to that of the other paralogs. The mutated versions were then overexpressed alongside native *ORCA3* in flower petals in two independent infiltration series (Figures 5C–F and Supplementary Figure 7, respectively). Overall, our results pointed to two key AARs leading to changes in MIA target gene expression. Compared to overexpression of native *ORCA3*, overexpression of *ORCA3*^*K101R*^ (corresponding to an AAR change occurring in ORCA2, 4, 5, and 6) generally enhanced the expression of MIA pathway genes (Figure 5C, Supplementary Figure 7). By contrast, overexpression of *ORCA3*^*P107L*^ (corresponding to one of the AAR changes in ORCA4) led to reduced or abolished levels of MIA pathway genes that were partly recovered in the double mutant *ORCA3*^*K101RP107L*^ (corresponding to AARs in ORCA4) (Figure 5C). Noteworthy, overexpression of *ORCA3*^*K101RP107L*^ still led to a lower up-regulation of *root MIA* genes than *ORCA3* or *ORCA3*^*K101R*^, suggesting that the combination of these two AARs contributes to the differential target gene profile of ORCA3 and 4. The impact of the other AARs appeared to be less specific. Overexpression of the *ORCA5*-like *ORCA3*^*K101RK110R*^ led to an expression profile similar to that upon overexpression of *ORCA3*^*K101R*^. Also, the much lower level of MIA target gene up-regulation by ORCA6 compared to the other ORCAs was not explained by the selected AARs [compare the overexpression of *ORCA3*^*K101RK110T*^ (Figure 5C) and *ORCA3*^*D117N*^ (Supplementary Figure 7)]. However, we did not produce a triple mutant in which all three AARs would correspond to those of ORCA6. The observed differential target gene profiles upon overexpression of *ORCA3* mutants were also reflected at the metabolite level (Figures 5D–F, Supplementary Figure 8 and Supplementary Table 7). Noteworthy, in particular the levels of 16-hydroxytabersonine, akuammicine, 16-methoxyhörhammericine and 16-hydroxylochnericine (among others) upon overexpression of *ORCA3* versus the ORCA2-like *ORCA3*^*K101R*^ and the ORCA4-like *ORCA3*^*K101RP107L*^ were very similar to those upon overexpression of the actual *ORCA2*, *3*, and *4* (Figure 4G and Supplementary Figure 6). We further performed transactivation assays with ORCA3, ORCA3^*K101R*^, ORCA3^*P107L*^, ORCA3^*K101RP107L*^, and ORCA3^*K101K110R*^ on *pSTR*, *pGO*, *pTAT* and *pMAT* (Figure 5G). Similarly to what was observed in the flower petal overexpression experiments ORCA3^*K101R*^ appeared to have a higher overall transactivation effect than ORCA3 on the four promoters whereas the double mutant ORCA3^*K101RP107L*^ transactivated pGO at significantly higher levels than ORCA3 but not the other promoters.

### Synergistic Up-Regulation in Combination With De-Repressed CrMYC2a^*D126N*^ Is Both ORCA- and Target Gene-Dependent

**FIGURE 7:**
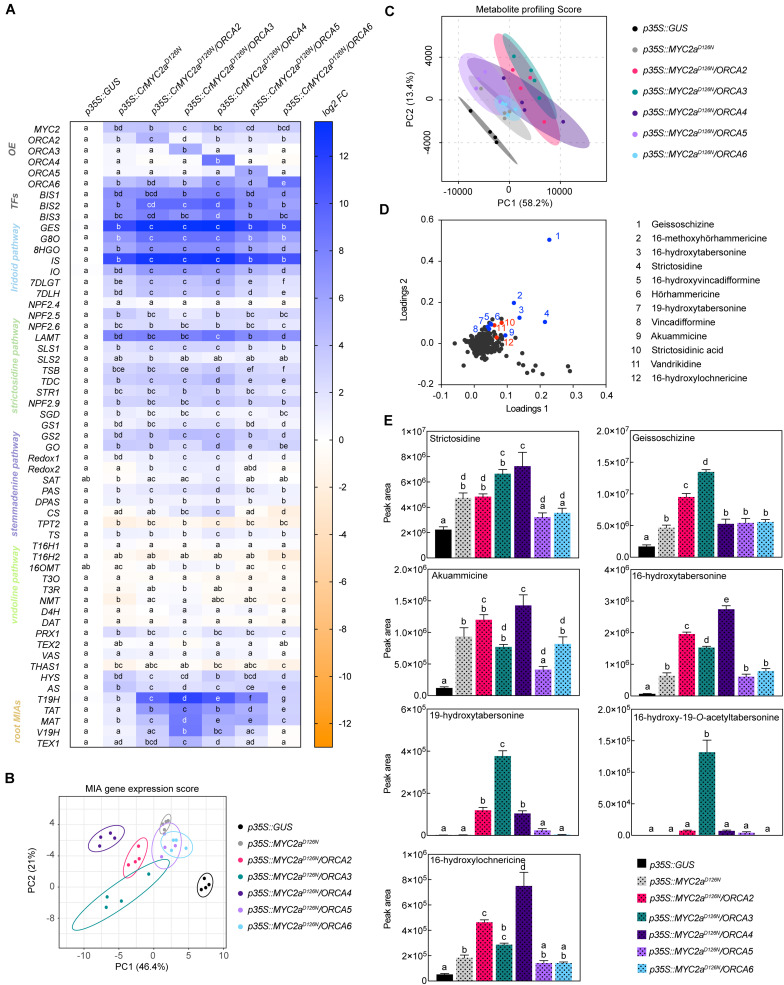
Combinatorial overexpression of *CrMYC2a*^*D216N*^ and *ORCAs*. **(A)** Expression profile of MIA pathway genes and overexpression levels. For each pathway gene, cells labeled with different letters represent statistically significant differences (*P* < 0.05, ANOVA with Tukey’s correction for multiple comparisons). Note that synergistic up-regulation of particularly root MIA genes is most pronounced by combinatorial overexpression of *CrMYC2a*^*D126N*^ and *ORCA3*. **(B)** PCA of the MIA gene expression data is suggesting that in particular the combinatorial overexpression of *CrMYC2a*^*D126N*^ and *ORCA2*, *3*, and *4* lead to differential changes in the MIA gene expression pattern, whereas combinatorial overexpression with *ORCA5* or *6* does not differ substantially from overexpression of *CrMYC2a*^*D126N*^ alone. PCA was performed with ClustVis (Metsalu and Vilo, 2015). **(C)** PCA of metabolite profiling data. **(D)** Loading plot of PCA with selected known MIAs (blue) and MIAs identified in this study (red). **(E)** Levels of selected MIA metabolites expressed in average total ion current (TIC). The levels of all measured MIA metabolites can be found in the supporting information (Supplementary Figure 9; Supplementary Table 8). Columns labeled with different letters represent statistically significant differences (*P* < 0.05, ANOVA with Tukey’s correction for multiple comparisons). Error bars are standard error of the mean (SEM).

Previously, we have shown that combinatorial overexpression of *ORCA3* and the de-repressed *CrMYC2a*^*D126N*^ variant leads to a synergistic up-regulation in particular of the *root MIA genes* compared to independent overexpression (Schweizer et al., 2018). Based on this and our experiments above that suggest that the ORCA TFs to some extent up-regulate different subsets of MIA pathway genes, we next investigated whether the combined overexpression of *CrMYC2a*^*D126N*^ with *ORCA2*, *4*, *5*, or *6* leads to a synergistic up-regulation of target genes similar as with *ORCA3* and if different subsets of MIA pathway genes would be up-regulated (Figure 7A). Indeed, a PCA depicted that in particular the combined overexpression of *CrMYC2a*^*D126N*^ with *ORCA2*, *3*, or *4* led to differences in the overlapping MIA pathway gene expression profiles (Figure 7B). Accordingly, PCA analysis of the MS data (Figure 7C) revealed somewhat differential metabolite profiles for the combination of *CrMYC2a*^*D126N*^ with these three *ORCA* genes rather than with *ORCA5* or *6*, or upon overexpression of *CrMYC2a*^*D126N*^ alone; these changes were due to differential accumulation of MIAs (Figure 7D). With regard to synergistic effects, the combined overexpression of *CrMYC2a*^*D126N*^ with *ORCA2* or *4*, synergistically up-regulated the *root MIA* genes, but to a lower extent than with *ORCA3* (Figure 7A). Accordingly, at the metabolite level, the *root MIAs* 19-hydroxytabersonine and 16-hydroxy-19-O-acetyltabersonine accumulated to much higher levels upon *CrMYC2a^*D126N*^/ORCA3* overexpression than upon overexpression of any of the other combinations (Figure 7E). Notably, no synergistic, if anything an additive up-regulation was observed for *GO*, although it was up-regulated upon overexpression of *CrMYC2a*^*D126N*^ and *ORCA4* alone (Figures 4D, 7A). Likewise, the level of the respective metabolic product akuammicine was only less than 2-fold higher in the samples with combined overexpression of *CrMYC2a*^*D126N*^ with *ORCA4*, compared to when *CrMYC2a*^*D126N*^ was overexpressed alone (Figure 7E). These two observations suggest that the ORCA-CrMYC2a^*D126N*^ synergistic module is partly TF-dependent, i.e., more pronounced for ORCA3 and, moreover, target gene-dependent, i.e., specific to some of the *root MIA* genes. To further confirm these observations promoter transactivation assays on *pSTR*, *pGO*, *pTAT*, and *pMAT* were carried out (Figure 6). Synergistic transactivation occurred particularly in the case of the *root MIA* gene promoter *pTAT* in combination with ORCA3 but could not be confirmed for the other root MIA promoter *pMAT*. However, transactivation levels for *pMAT* were generally low and it is possible that the 2-kB fragment did not contain all necessary *cis* regulatory sites. We were unable to clone the promoter region of *T19H* in several attempts. Transactivation of *pGO* by CrMYC2a^*D126N*^ and ORCA4 was not synergistic, again as observed in overexpression in flower petals.

**FIGURE 8:**
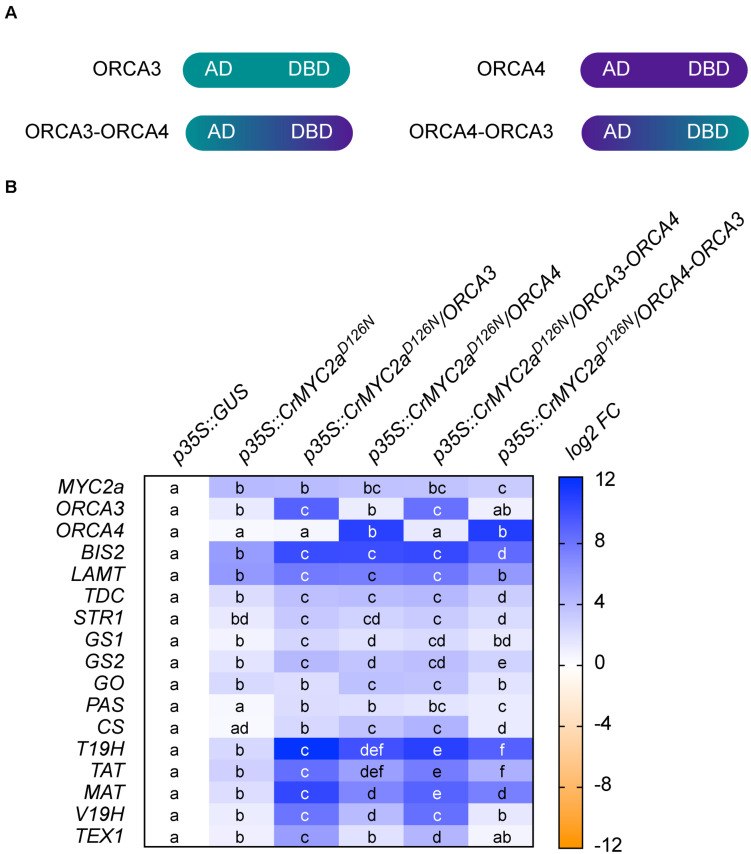
Swapping of DNA-binding domain (DBD) and activation domain (AD) of ORCA3 and 4. In order to get insight into the role of the AD in the synergistic up-regulation of MIA target genes, the respective DBDs and ADs of ORCA3 and 4 were swapped. **(A)** Cartoon showing the domain organization of ORCA3 and 4 and the respective chimeric proteins. **(B)** Combinatorial overexpression of *CrMYC2a*^*D126N*^ with *ORCA3*, *4* or chimeric proteins *ORCA3-ORCA4* or *ORCA4-ORCA3*. These results suggest that the AD of ORCA3 improves the synergistic up-regulation of target genes. Cells labeled with different letters represent statistically significant differences (*P* < 0.05, ANOVA with Tukey’s correction for multiple comparisons).

To gain more insight into how a synergistic up-regulation could be mediated at the molecular level and to possibly shift the subset of target genes of this synergy, we next made chimeric constructs. Here, we focused on *ORCA3* and *4*, because their level of overexpression was most similar and the level of up-regulation of target genes was the strongest for these two paralogs. In these chimeric constructs, the N-terminal AD of ORCA3 was fused to the C-terminal DBD of ORCA4 (ORCA3-ORCA4) and *vice versa* (ORCA4-ORCA3) (Figure 8A). Overexpression of these “*ORCA*-swaps” in combination with *CrMYC2a*^*D126N*^ led to shifts in the up-regulation of root MIA genes (Figure 8B). Overexpression of *ORCA3-ORCA4* led to a 2 to 4 fold higher up-regulation of *root MIA genes* than overexpression of native *ORCA4*, whereas overexpression of *ORCA4-ORCA3* led to around 4-fold lower up-regulation than overexpression of native *ORCA3*. These results indicate that the AD of ORCA3 might at least in part be necessary for the synergistic up-regulation. On the other hand, overexpression of *CrMYC2a*^*D126N*^/*ORCA3-ORCA4* did not lead to a higher up-regulation of *GO* than overexpression of *CrMYC2a*^*D126N*^/*ORCA4*, further supporting the previous observation that the synergistic up-regulation is specific to *root MIA* genes, i.e., target gene specific.

### Putative Cell-Specific Regulation of the *Vindoline* Branch

Data from the cell type-enriched transcriptomes pointed toward a number of epidermis-specific TFs that might be candidate regulators of the epidermis-specific expression of some MIA pathway genes (*strictosidine* and *stemmadenine* branches and parts of the *vindoline* branch and catharanthine biosynthesis). Among these potential candidates, two MYB TFs were highly expressed in the stem epidermis (Supplementary Figure 10B). Both MYB TFs, hereafter named MYB96 and MYB96b, appeared to be potential orthologs of AtMYB96 and 94 (Supplementary Figure 10A), which amongst others positively regulate cuticular wax biosynthesis genes in *Arabidopsis* (Seo et al., 2011; Lee and Suh, 2015; Lee et al., 2016). In a preliminary screen, both MYB TFs were found to transactivate the *T3R* promoter in tobacco protoplasts, albeit not additively (Supplementary Figure 10C). Overexpression of *MYB96* in flower petals also led to an up-regulation of expression of *T3R* and of a putative ortholog of the *Arabidopsis* cuticular wax biosynthesis gene *CER1*, but this was not the case for *MYB96b* (Supplementary Figure 10D). Nonetheless, the combined overexpression of *MYB96* and *MYB96b* led to a very small increase in catharanthine levels, which was not observed upon overexpression of *MYB96* alone (Supplementary Figures 10G,H). Notably however, because we neither detected any up-regulation of other *vindoline pathway* genes by *MYB96* (Supplementary Figure 10D) nor did MYB96 transactivate promoters of other vindoline pathway genes in tobacco protoplasts (Supplementary Figure 10C), MYB96 appears to be specific to *T3R*. In an attempt to enhance the vindoline content we further overexpressed *MYB96* in combination with the above-described *MYC2a*^*D126N*^-*ORCA4* module, which led to a higher availability of the vindoline precursor 16-hydroxytabersonine (Supplementary Figure 11). We observed that overexpression of *MYB96*, in addition to the observed up-regulation of *T3R* and *CER1*, also led to a slight but statistically significant up-regulation (around 2-fold) of *stemmadenine pathway* genes and to a significant up-regulation of the catharanthine exporter gene *TPT2* (at least 4-fold) (Supplementary Figure 11). However, at the metabolite level, the overexpression of *MYB96* solely or in combination with *ORCA4* and/or *CrMYC2a*^*D126N*^ did not lead to increased vindoline or catharanthine content (Supplementary Figure 11D and Supplementary Table 10). Furthermore, it should be noted that overexpression of *CrMYC2a*^*D126*^ appeared to lead to a down-regulation of most vindoline pathway genes except *T3R* (Supplementary Figure 11A), further suggesting that a synergistic/additive action between these TFs seems unlikely.

## Discussion

While *C. roseus* is traditionally known as a medicinal plant, it is also increasingly being established as a model species for plant specialized metabolism in general. Thanks to the extensive work in the past decades by several labs, including ours, our knowledge about the network of MIA pathway branches represents one of the most detailed known biosynthesis networks of specialized metabolites, both at the enzyme and regulator level. In this study, we first aimed to identify new regulators for the different MIA branches, using the currently available pathway information. Thereby, we further underscore the importance of specific ORCA and bHLH TFs as modular regulatory hubs for different MIA pathway branches. While these TF families have been already generally implicated in the regulation of specialized metabolism, our study additionally provides evidence for the functional specialization of TF paralogs due to specific AAR changes in the DBD. These results are predominantly based on overexpression in flower petals. Despite being a powerful experimental system, it comes with the limitation that levels of overexpression of different TFs may differ and thus skew the results. By additionally performing promoter transactivation assays in tobacco protoplasts we were however able to generally confirm our results for a selection of available promoter fragments. In the future additional experiments such as individual knocking down of specific ORCAs by Virus Induced Gene Silencing (VIGS) (Liscombe and O’connor, 2011) or knocking out by genome editing could be performed to further clarify the specific roles of the different ORCAs.

### Functional Redundancy and Diversification Among BIS and ORCA TF Clusters

Clusters of TF paralogs appear among various species and families; the orthologs of *ORCA* genes occur for instance also as gene clusters in tobacco, tomato and potato (Shoji et al., 2010; Cárdenas et al., 2016; Kajikawa et al., 2017; Shoji and Yuan, 2021) and so do the *Arabidopsis* BIS orthologs *bHLH18*, *19*, and *20* (Cui et al., 2018). Our findings indicate that redundancy and sub-functionalization may occur at the same time for different target genes. In case of the ORCA cluster, we observed that they activate those pathway genes necessary to synthesize common precursors, i.e., “early pathway genes” in a redundant manner. Conversely, later MIA pathway branches are then to some extent controlled by individual members. ORCA3, for instance, is more specific to some of the root MIAs and ORCA4 to the akuammicine branch. ORCA6 seems to have a rather limited impact on known MIA pathway genes, with the exception of a moderate up-regulation of the root MIA genes. The latter results are consistent with a recent study (Singh et al., 2020). These observations are similar to what has been described for the *Medicago truncatula* BIS orthologs, which redundantly activate early triterpene saponin biosynthesis genes but differentially activate downstream branches in the triterpene biosynthesis pathways and/or in different organs (Mertens et al., 2016; Ribeiro et al., 2020). The members of the ORCA orthologous ERF gene clusters in tobacco and tomato also show differences with regard to their sequences and their tissue and environmental specific expression profiles (Shoji et al., 2010; Cárdenas et al., 2016; Kajikawa et al., 2017; Shoji and Yuan, 2021). It has been suggested that members of these ERF gene clusters acquired a level of sub-functionalization similar to what is found for the ORCAs (Shoji and Yuan, 2021). For instance, in the case of the tobacco ERF genes it was very recently shown that two members have more impact on nicotine biosynthesis than the other members further indicating sub-functionalization (Hayashi et al., 2020). Although more research is needed to unravel the specific roles of individual members of TF clusters in more species it is tempting to speculate that TF gene duplications resulting in clusters and subsequent sub-functionalization might be a common strategy for plants to achieve a more specific regulation of specialized metabolic pathway branches.

### Specific AARs in the DBDs of ORCA TFs Modulate Target Gene Specificity

Despite increasing knowledge about the DNA binding motifs of different TF families, much less is known about the specific binding affinities of TFs belonging to the same clade. A previous study compared the differential DNA binding affinities of ORCA3 and its orthologs from different species and defined some AARs conferring DNA binding specificity (Shoji et al., 2013). Here, we specifically compare all members of clade XIa AP2/ERFs within the same species under the same experimental conditions and determine AARs conferring specificity that had not been identified previously. It will be interesting to further investigate whether similar AAR changes also confer target gene specialization in the other species with several paralogs and, moreover, determine paralog-specific DNA binding site sequences. Ultimately, further knowledge about such key AARs may offer more precise possibilities for metabolic engineering. For instance, the native version of a given TF might not be the most desirable one for flux into a specific pathway branch but an engineered version in which a key amino acid is substituted might do so. This smallest change for metabolic engineering purposes might be referred to as metabolic editing (Swinnen et al., 2019). Similarly, once the respective DNA binding motifs of a TF are known, specific target genes could be brought under control of that TF by promoter base editing (Swinnen et al., 2016).

### MYC2-a Long-Known Hub With Remaining Mechanistic Mysteries

As described here and elsewhere, many MIA pathway genes are up-regulated by JA, which can be mimicked by overexpression of the de-repressed *CrMYC2a*^*D126N*^ (Schweizer et al., 2018). In addition, we have previously shown that the combinatorial overexpression with *ORCA3* leads to an up-regulation of the *root MIA pathway* genes and other target genes in a synergistic manner (Schweizer et al., 2018). A similar synergistic up-regulation of target genes has previously been observed between the respective tomato and tobacco MYC2 and AP2/ERF orthologs, suggesting an evolutionary conservation of this combinatorial TF module (De Boer et al., 2011; Cárdenas et al., 2016). Here, we observe that in *C. roseus* the synergistic transactivation is to some extent paralog specific, as the level of synergy is higher in combination with ORCA3 than with ORCA4 for instance. Although we cannot rule out that this is (in part) invoked by different protein stabilities, our results further suggest that this is at least in part due to the AD as we could observe that the AD of ORCA3 significantly enhanced the level of target gene up-regulation when fused to the DBD of ORCA4, whereas the reverse swap rather led to a reduced up-regulation. Although ADs are generally little conserved at the sequence level, it has been shown that specific structural features or subdomains are essential for the TF activity, for instance for the recruitment of the transcriptional machinery (Staby et al., 2017). Noteworthy, *in silico* analyses of the ORCA ADs predict structural differences, which further supports that they are diverse. It remains to be uncovered if and which of these structural differences confer the ORCA3-specific synergistic up-regulation of target genes and if this is for instance mediated by direct physical interaction between the TFs or by other mechanisms. Likewise, more investigation is needed to clarify how the synergistic up-regulation is restricted to specific target genes, i.e., the *root MIA* genes, whereas the up-regulation of other target genes, for instance the BIS TFs, does not appear synergistic.

### Outlook: Touching the Boundaries of Co-expression Analysis as a Tool to Find Regulator Candidates

Co-expression analysis, which is based on the assumption that the expression profiles of functionally related genes correlate, remains one of the most popular and successful strategies to identify both metabolic pathway genes and regulatory TFs (Movahedi et al., 2012; Goossens, 2015; Wisecaver et al., 2017). However, it has also been noted previously that a successful outcome is greatly dependent on the combination of datasets included in the analysis, the specific methodology used and others (Goossens, 2015; Uygun et al., 2016). In the case of our study, there are a number of conceivable reasons, technical or mechanistic, why we were not able to unambiguously uncover regulators for all MIA branches yet, e.g., the *vindoline pathway*. First, the number of available expression datasets for *C. roseus* is still limited to around 80. Hence, if the pathway branch of interest does not show a (highly) differential expression profile among these datasets, the correlation with expression profiles of regulator candidates might be low. More specifically, the induction of regulatory TFs identified in past studies on *C. roseus* and of their target MIA pathway genes upon JA exposure is highly correlated because of the renowned amplification loops in the JA signaling cascade (De Geyter et al., 2012), which facilitated their discoveries. By contrast, the *vindoline pathway* is not induced upon JA exposure and, apart from a higher expression in green tissues, it is not differentially expressed otherwise. Hence, this pathway branch is either not differentially expressed in response to a specific environmental condition, or a discriminating RNA-Seq dataset for this hypothetical condition is not yet available. Further, transcriptional regulation may involve independently acting TFs individually mediating the specific responses to environment, development, cell type and others. The expression of such a condition-related TF might thus only be strongly correlated to its MIA target genes in a subselection of datasets and not across all datasets. Mining of selected dataset combinations or application of a bi-clustering approach to identify significant co-expression in a subset of samples, could improve this and could be recommended for future co-expression analyses, but would possibly also yield a much longer list of candidates. Next, at the experimental level, it is possible that the candidate list indeed contains positive regulators but that (i) their cloning failed, (ii) they were not sufficiently overexpressed, (iii) their target gene induction required the combined action of TFs (analogous to the ORCA3-CrMYC2a^*D126N*^ module) but the respective TFs did not happen to be in the same co-overexpression group, (iv) they were not active in flower petals due to epigenetic or post-translational mechanisms.

Our study, together with the vast work previously published by different research groups extensively illustrates that MYC2 TFs globally up-regulate MIA biosynthesis together with the branch-specific ORCA and BIS TFs with the exception of the *vindoline* branch. This raises questions about the biological function of vindoline and/or the intermediates in this pathway branch. While MIA biosynthesis has been generally connected to defense against for instance caterpillars to some extent (Dugé De Bernonville et al., 2017), the chemical ecology of the different specific MIAs is largely unknown. The decoupled expression profile of the *vindoline pathway* from other MIA branches and the absence of up-regulation by CrMYC2a^*D126N*^ (rather a potential down-regulation) concomitant with a light-induced regulation raise the possibility of other or additional biological functions of vindoline. Nevertheless, the biosynthesis (and/or putative transport) of precursors must be assured, a process that appears not to be light regulated. It is clear that additional studies, likely not based on transcriptome mining solely, are therefore necessary to further investigate how these different signals are integrated to regulate flux through the distinct MIA pathway branches.

## Data Availability Statement

The datasets presented in this study can be found in online repositories. The names of the repository/repositories and accession number(s) can be found in the article/Supplementary Material.

## Author Contributions

MC and AG designed the research and wrote the manuscript with consent from all other authors. MC performed the research and data analysis with contributions from DM, FS, LD, RD, JG, TM-C, FM-H, and MS. DV and KV performed RNA-Seq data and co-expression analysis and inferred candidate regulators. JP collected and analyzed metabolite data and performed compound identification. All authors contributed to the article and approved the submitted version.

## Conflict of Interest

The authors declare that the research was conducted in the absence of any commercial or financial relationships that could be construed as a potential conflict of interest.

## Footnotes

1 http://medicinalplantgenomics.msu.edu/index.shtml

2 https://eurofinsgenomics.eu

3 https://www.metaboanalyst.ca/

## Supplementary Material

Supplementary Figure 1 MIA gene expression levels upon bulk overexpression of TF candidates.

Supplementary Figure 2 Separate overexpression of positive TF candidates.

Supplementary Figure 3 Promoter transactivation of iridoid pathway genes by combinations of BIS.

Supplementary Figure 4 Comparison of structural features and motifs of ORCA activation domains (ADs).

Supplementary Figure 5 Expression profiles of MIA biosynthesis genes and regulators in analyzed RNA-seq data.

Supplementary Figure 6 MIA levels upon overexpression of *ORCAs*.

Supplementary Figure 7 MIA gene expression levels upon overexpression of *ORCA3* single mutants.

Supplementary Figure 8 MIA levels upon overexpression of *ORCA3* mutants.

Supplementary Figure 9 MIA levels upon combinatorial overexpression of *CrMYC2a*^*D126N*^ and *ORCAs*.

Supplementary Figure 10 Orthologs of cuticular wax regulators up-regulate the vindoline pathway gene *T3R*.

Supplementary Figure 11 Combinatorial overexpression of *ORCA4, CrMYB96, CrMYC2a^*D126N*^*.

Supplementary Table 1 List of RNA-Seq datasets used.

Supplementary Table 2 Oligonucleotide primers used for cloning of coding sequences.

Supplementary Table 3 Oligonucleotide primers used for cloning of promoter fragments.

Supplementary Table 4 List of all cloned TFs and promoter fragments and GeneBank IDs.

Supplementary Table 5 Oligonucleotide primers used for qPCR.

Supplementary Table 6 MIA levels upon overexpression of *ORCAs*.

Supplementary Table 7 MIA levels upon overexpression of *ORCA3* mutants.

Supplementary Table 8 MIA levels upon combinatorial overexpression of *CrMYC2a*^*D126N*^ and *ORCAs*.

Supplementary Table 9 MIA levels upon overexpression of *MYB96/b*.

Supplementary Table 10 MIA levels upon overexpression of *MYB96/b*, *CrMYC2a*^*D126N*^, and *ORCA4*.

Supporting Dataset I *C. roseus* expression atlas.

Supporting Dataset II MIAs identified in this study.
