# Supplementary material for: Subfunctionalization of Paralog Transcription Factors Contributes to Regulation of Alkaloid Pathway Branch Choice in Catharanthus roseus
Source: Front Plant Sci. 2021 May 25;12:687406. doi: 10.3389/fpls.2021.687406 (PMC8186833; doi:10.3389/fpls.2021.687406)
Supplement: Supplementary Figure 1 — MIA gene expression levels upon bulk overexpression of TF candidates. [file Image_1.pdf]

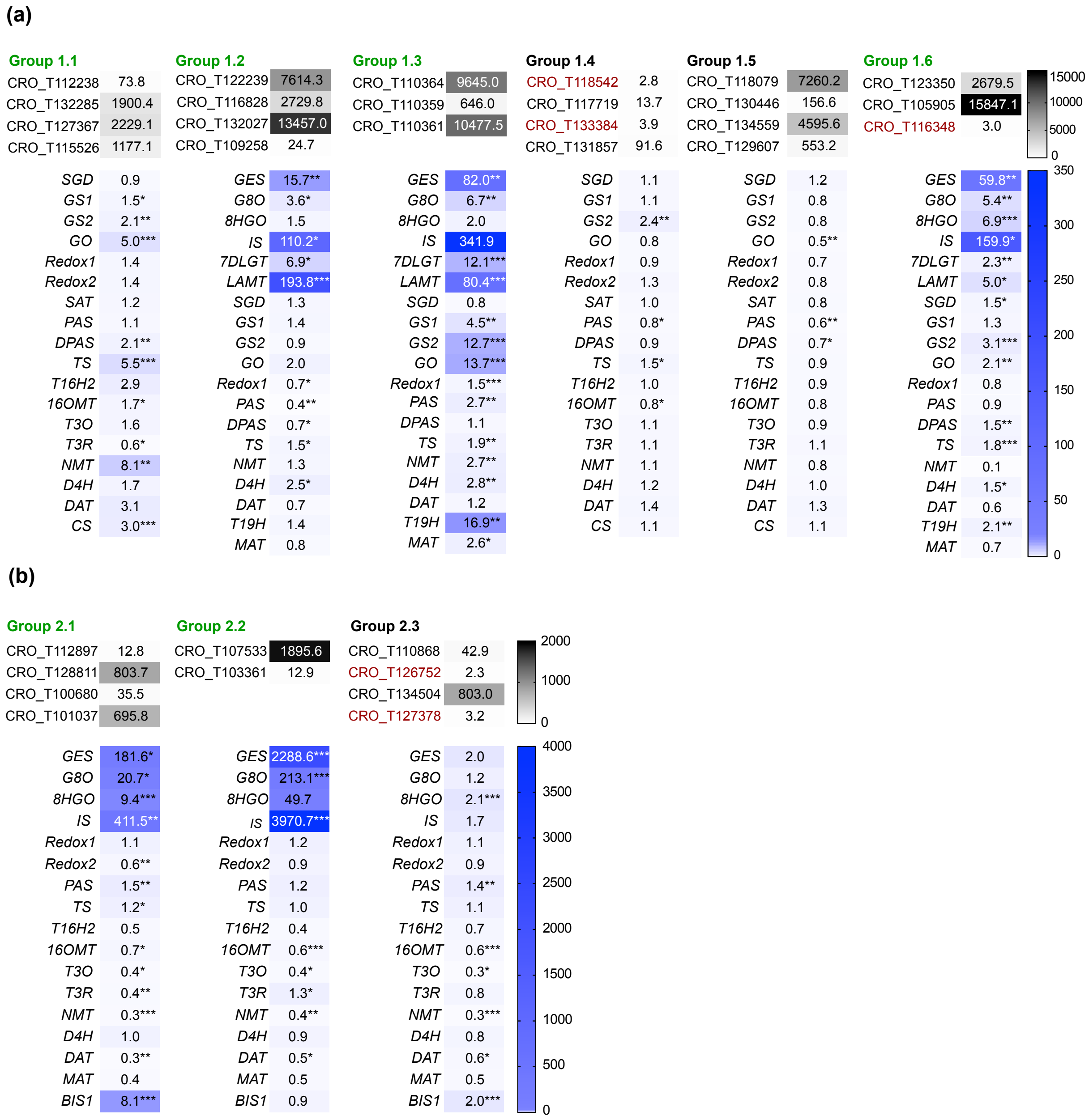

SUPPLEMENTARY FIGURE S1 MIA gene expression levels upon bulk overexpression of TF candidates. Up to four TF candidates were bulk overexpressed via flower petal infiltration with *A. tumefaciens* in two rounds (a and b), and the expression level of MIA genes subsequently measured by qPCR. Gene expression levels are expressed as fold changes relative to that of the *p35S::GUS* control. *P<0.05, **P<0.01, ***P<0.001 calculated by two-tailed Student's *t*-test of four biological replicates. Groups marked in green were chosen for subsequent separate infiltration of TF candidates. Gene IDs labeled in red were only mildly overexpressed (<5-fold) and not taken along for further analyses.

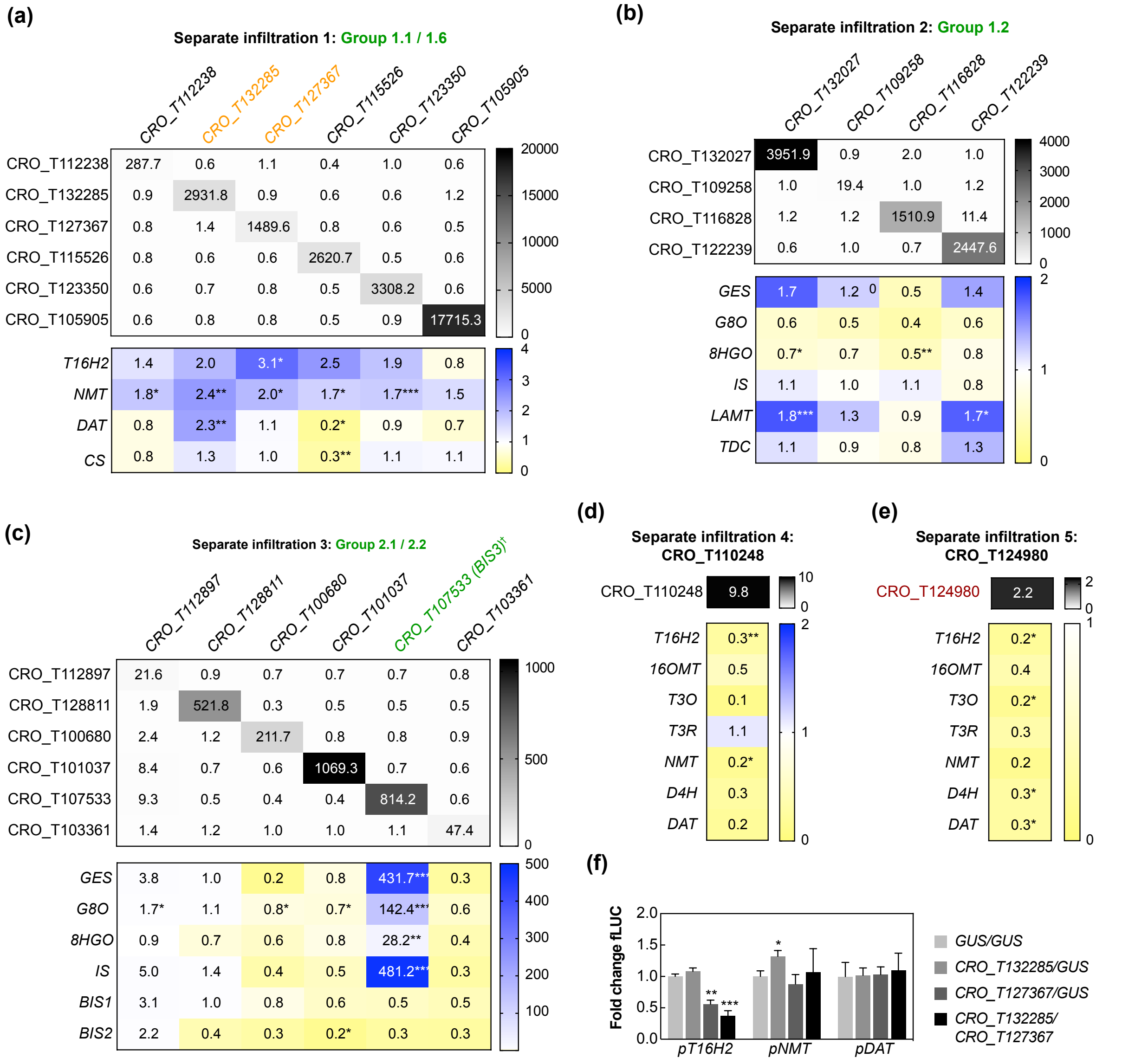

SUPPLEMENTARY FIGURE S2 Separate overexpression of positive TF candidates. TF candidates selected from results from bulk overexpression (Supplementary Figure S1) were infiltrated separately in independent infiltration series. Gene expression levels measured by qPCR are expressed as fold changes relative to the expression in the *p35S::GUS* control. *P<0.05, **P<0.01, ***P<0.001 calculated by two-tailed Student's *t*-test, four biological replicates. Gene IDs labeled in red were only mildly overexpressed (<5-fold).

(a) Genes from screening group 1.1 and 1.6 that were well overexpressed (see Supplementary Figure S1) were overexpressed separately. Overexpression of CRO_T132285 and CRO_T127367 (orange colored) led to a statistically significant >2-fold up-regulation of some vindoline genes. CRO_T123350 and CRO_T105905 can be classified as bHLH Clade IVa TFs due

to sequence homology, and were therefore additionally tested for up-regulation of iridoid pathway genes (Fig. 2).

(b) Separate overexpression of genes from screening group 1.2. The previously observed up-regulation of early MIA pathway genes was not confirmed.

(c) Separate overexpression of TF candidates from bulk overexpression groups 2.1 and 2.2. † CRO_T107533 (re-named *BIS3*) was identified as a *BIS* paralog (see Fig. 3).

(d) and **(e)** These TF candidates had been previously selected as potential MIA pathway regulators by independent co-expression analyses and overexpressed separately.

(f) In order to clarify their up-regulation effect on MIA genes upon overexpression in flower petals (see a), candidates CRO_T132285 and CRO_T127367 were tested in promoter transactivation assays in *N. tabacum* protoplasts. The absence of a consistent activation of these promoter fragments does not support a direct involvement in vindoline pathway regulation. *$P<0.05$, **$P<0.01$, ***$P<0.001$ was calculated by two-tailed Student's *t*-test. Error bars depict SEM of four biological replicates.

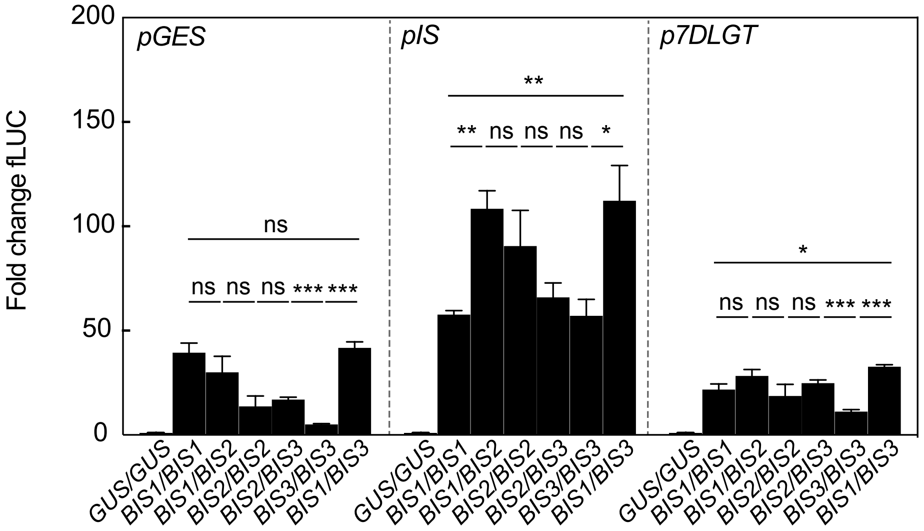

SUPPLEMENTARY FIGURE S3 Promoter transactivation of iridoid pathway genes by combinations of BIS'.

It was previously shown that BIS1 and BIS2 physically interact and to a certain extent synergistically up-regulate iridoid pathway target gene (Van Moerkercke et al. 2016). Here, transactivation assays of selected iridoid pathway promoter fragments (*pGES*, *pIS* and *p7DLGT*) were tested combinations of BIS'. An increase in transactivation by different BIS TFs was limited to transactivation of *pIS* and *p7DLGT* by BIS1 and 3 only. *N. tabacum* protoplasts were co-transfected with constructs containing the *fLUC* gene to be expressed under control of the indicated promoter fragments and constructs for overexpression of *BIS'* or *GUS* as a control. For individual overexpression of BIS1, 2 or 3 protoplasts were transfected with the double amounts of BIS1, 2 or 3 expression plasmids in order to compare effects of potential homo-dimers versus hetero-dimers. The y-axis shows fold change in normalized fLUC activity relative to the control transfection with GUS, set at 1. The error bars depict SEM of four biological replicates. Asterisks indicate statistically significant differences in transactivation (*P < 0.05, **P < 0.01, ***P < 0.01) as calculated by Student's *t*-test.

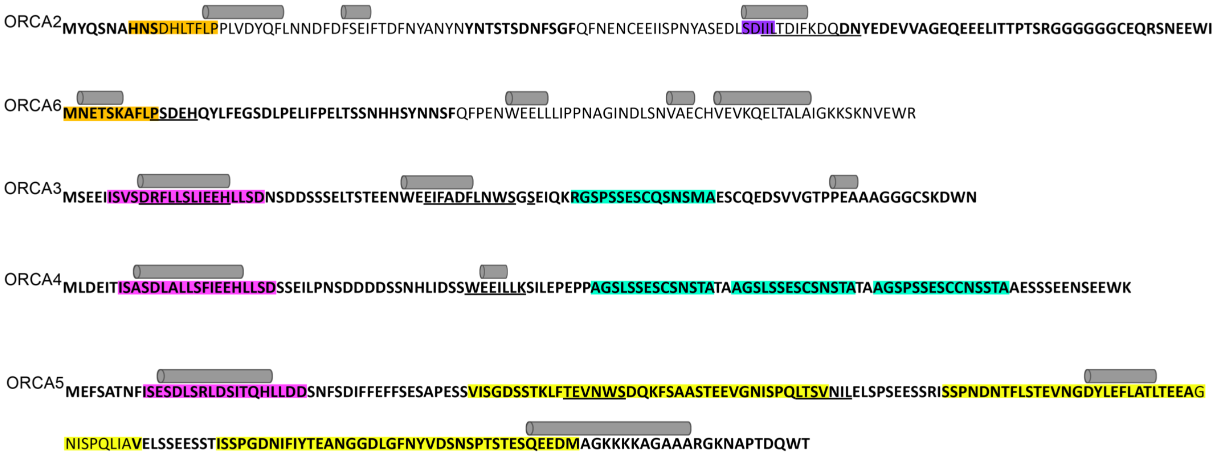

SUPPLEMENTARY FIGURE S4 Comparison of structural features and motifs of ORCA activation domains (ADs). While the DNA binding domain (DBD) is assumed to determine binding activity to specific DNA motifs, the AD is thought to confer the level of activation for instance by recruiting the transcriptional machinery and/or by interaction with other TFs or co-regulators. The observed differences in MIA target gene activation are only partly explained by differences in specific amino acid residues (AARs) in the DBD. In particular, a general lower activity (such as observed for the ORCA6 paralog) or the observed differences in synergistic up-regulation (i.e. higher for ORCA3 than for the other ORCAs) might be also caused by differences in the ADs. ADs commonly show a high level of disorder so that structural information by protein crystallization is scarce, and a very low level of AAR sequence conservation compared to the DBD, however structural features and motifs can be revealed using a combination of *in silico* methods (O'Shea et al., 2017). To compare ORCA AD sequences despite their low homology, we used different prediction and motif search programs. As expected, PONDR® VSL2 predicts long stretches of the ORCA ADs to be disordered (bold font); in case of ORCA3-5 practically the entire AD is predicted to be disordered (Xue et al., 2010). Within disordered regions, molecular recognition features (MoRFs) can occur. These short regions can undergo disorder-to-order transitions and bind protein partners, and can be predicted by MoRFpred (underlined sequence) (Disfani et al., 2012). The α-helices (gray cylinders above the sequence) predicted by PSIPRED 4.0 often coincide with MoRFs, further indicating that these short regions may indeed form secondary structures (Buchan and Jones, 2019). Moreover, we performed a MEME analysis to uncover motifs with sequence similarity (depicted by colors) (Bailey et al., 2015). In summary, the ADs of ORCA3, 4 and 5 share for instance in particular a motif at the N-terminus (pink), but they do not share any of the

predicted motifs or structural features with those of ORCA2 and 6. The observed differences in MIA gene up-regulation between the ORCA3 and 4 domain swaps (Fig. 7) may be due to specific motifs but this requires further future experimentation.

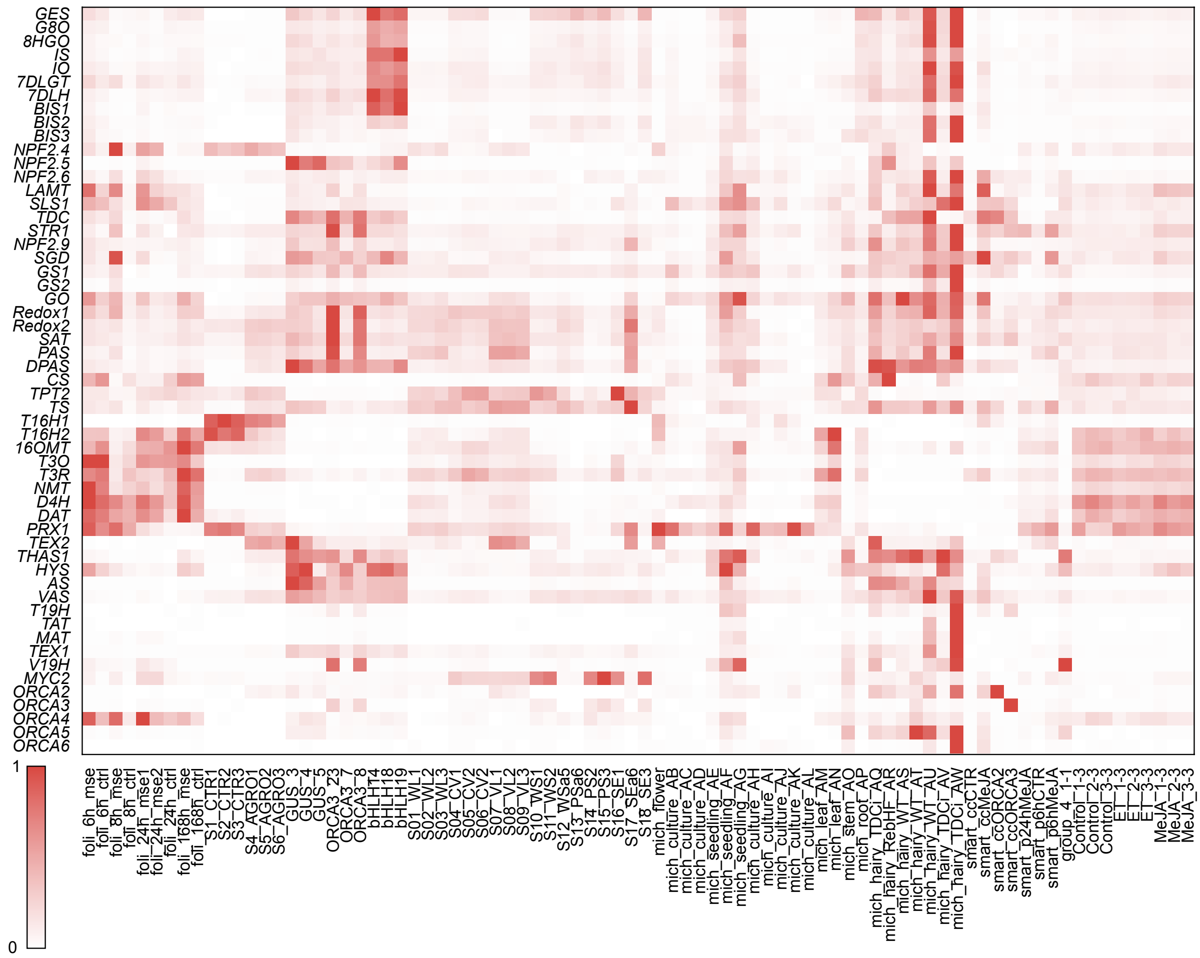

SUPPLEMENTARY FIGURE S5 Expression profiles of MIA biosynthesis genes and regulators in analyzed RNA-seq data.

Expression levels of MIA pathway genes and BIS and ORCA transcription factors are shown for all 81 RNA-seq samples included in the candidate selection analysis. TPM values were extracted from the expression atlas (Supporting dataset 1) and expression levels scaled from 0 (lowest TPM value) to 1 (highest TPM value) for each gene across all RNA-seq samples.

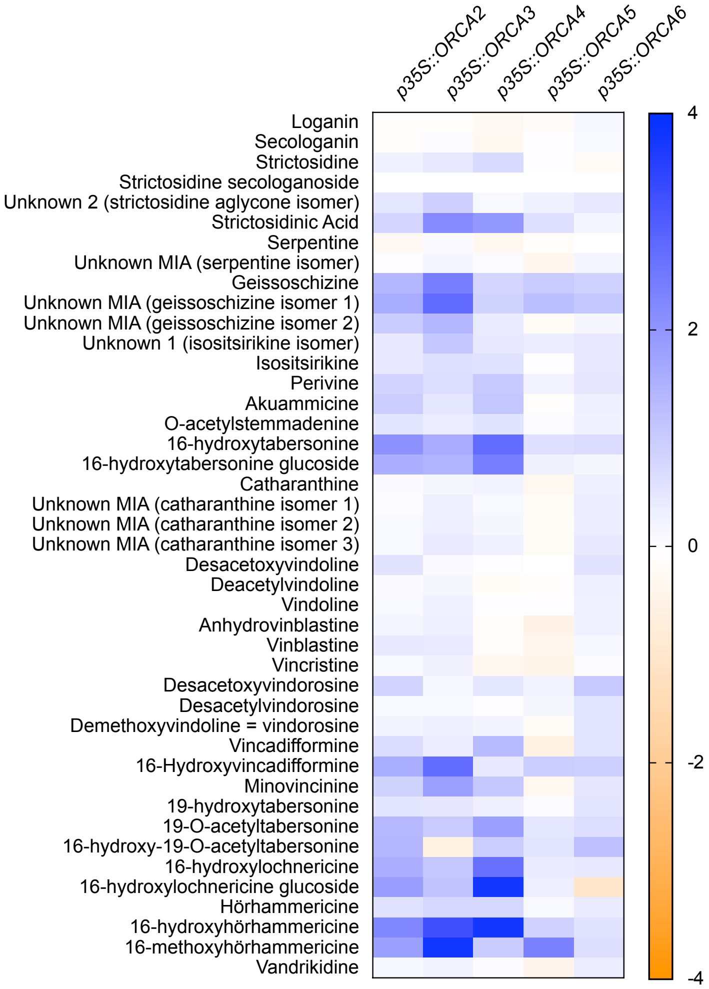

SUPPLEMENTARY FIGURE S6 MIA levels upon overexpression of *ORCAs*. Levels are expressed as log2 fold changes (FC) compared to the *p35S::GUS* control. Total ion current (TIC) values from this experiment, including statistical analysis, can be found in Supplementary Table S6.

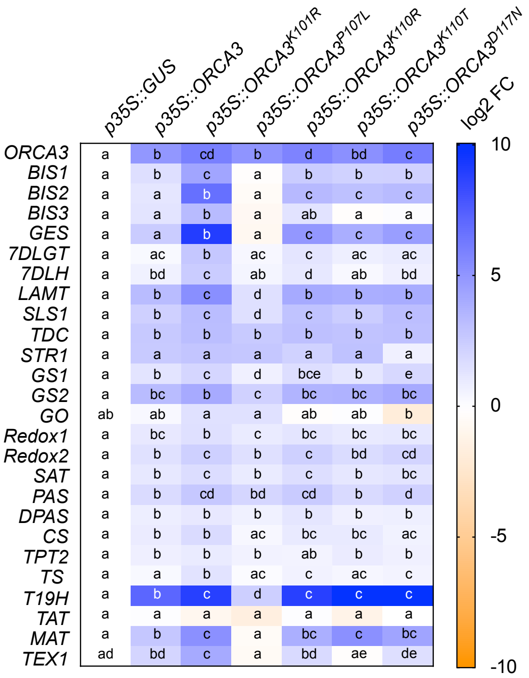

SUPPLEMENTARY FIGURE S7 MIA gene expression levels upon overexpression of *ORCA3* single mutants. The indicated AARs of ORCA3 were mutated to correspond to those of other ORCA TFs (see Fig. 5a). Gene expression levels measured by qPCR are expressed as average log2 FC relative to the *p35S::GUS* control. For each gene, different letters represent statistically significant differences ($P<0.05$, ANOVA with Tukey's correction for multiple comparisons).

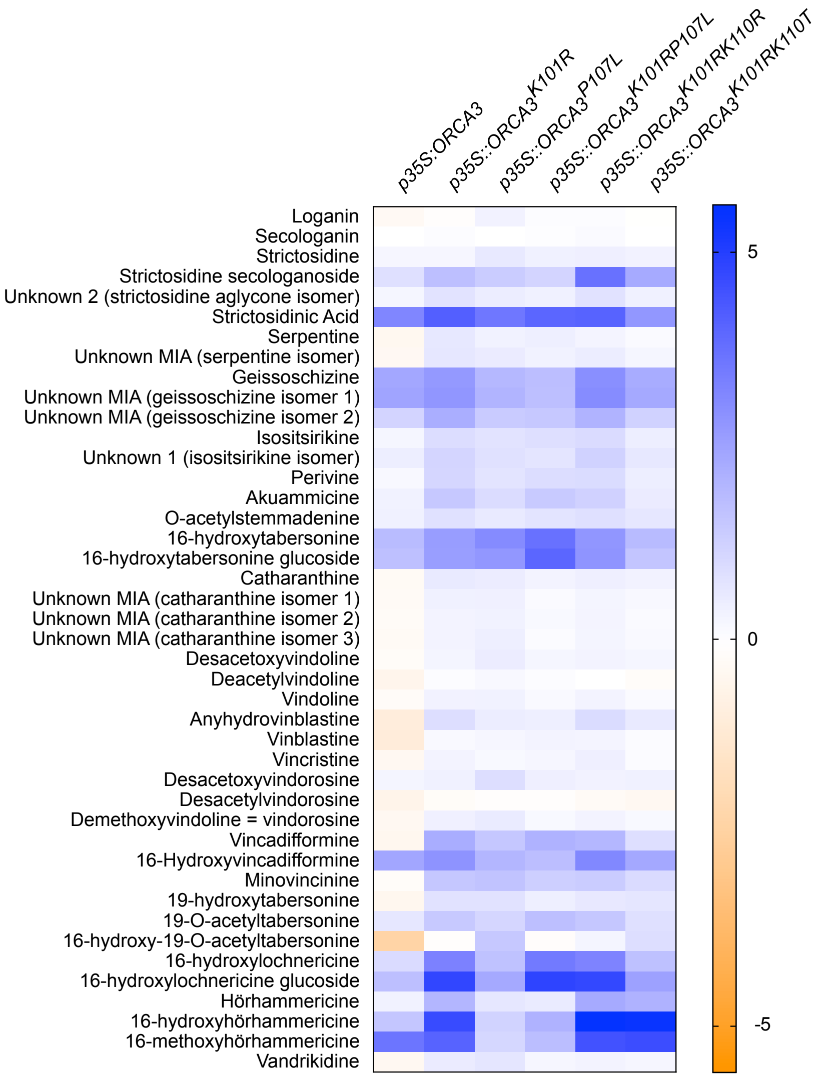

SUPPLEMENTARY FIGURE S8 MIA levels upon overexpression of *ORCA3* mutants. Levels are expressed as log2 FC compared to the *p35S::GUS* control. Total ion current (TIC) values from this experiment including statistical analysis can be found in Supplementary Table S7.

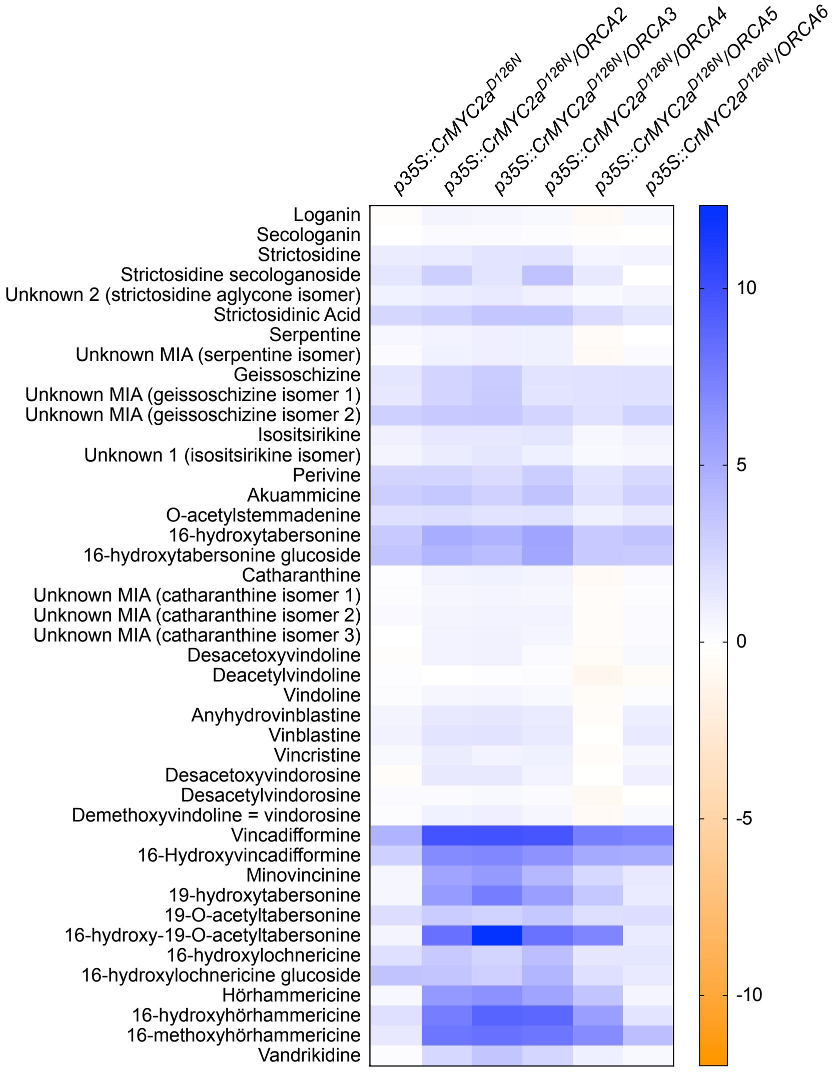

SUPPLEMENTARY FIGURE S9 MIA levels upon combinatorial overexpression of *CrMYC2a*^{D126N} and *ORCAs*. Levels are expressed as log2 FC compared to the *p35S::GUS* control. Total ion current (TIC) values from this experiment including statistical analysis can be found in Supplementary Table S8.

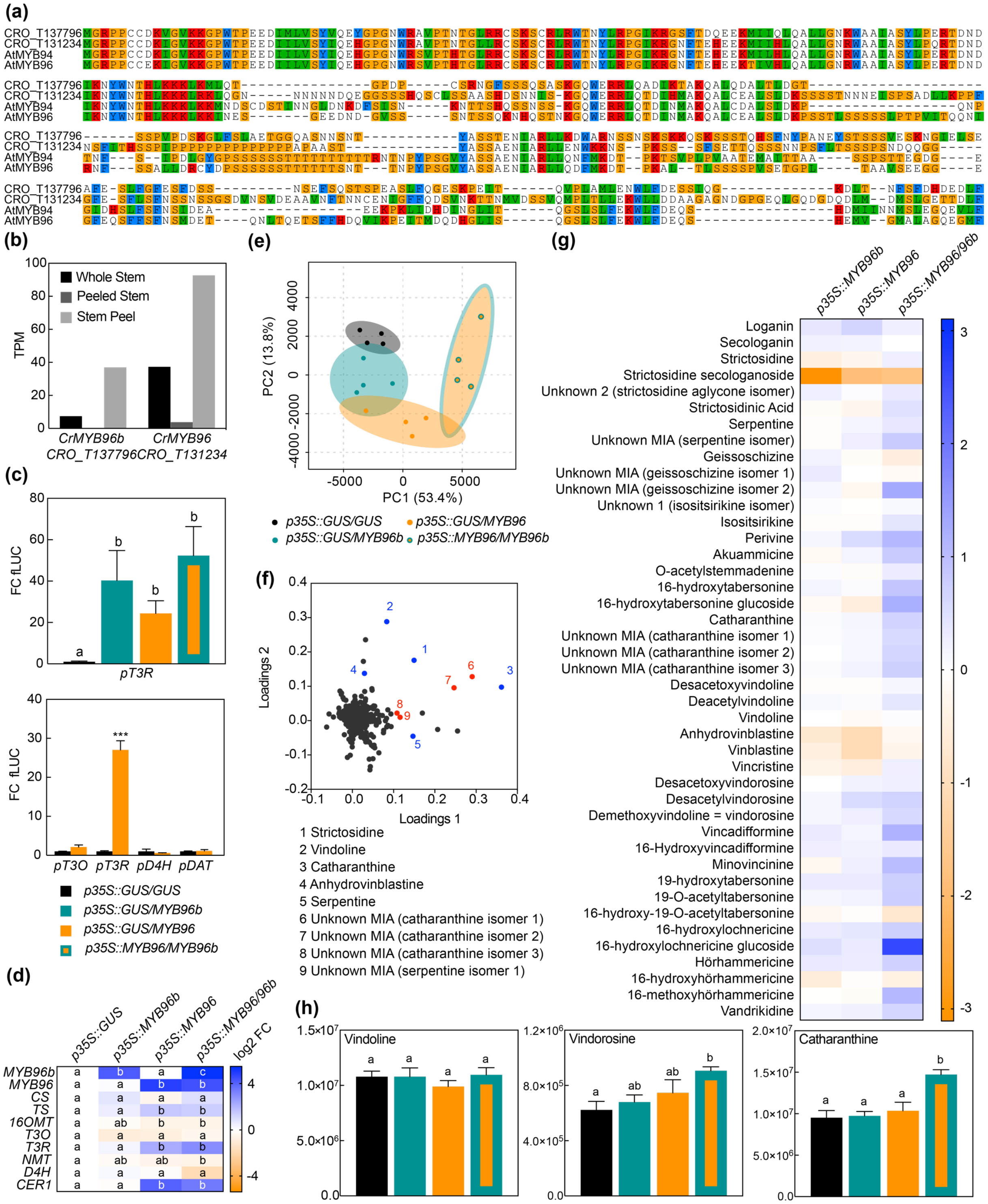

SUPPLEMENTARY FIGURE S10 Orthologs of cuticular wax biosynthesis regulators up-regulate the vindoline pathway gene *T3R*. The *A. thaliana* TFs MYB94 and 96 have been implicated in up-regulation of cuticular wax biosynthesis. When screening epidermis-enriched transcriptome data for potential epidermis-specific MIA biosynthesis regulators, the *C. roseus*

orthologs CRO_T137796 and CRO_T131234 appeared and were renamed CrMYB96b and CrMYB96, respectively.

(a) MUSCLE protein sequence alignment of *A. thaliana* MYB94 and 96 and *C. roseus* orthologs.

(b) Transcripts Per Kilobase Million (TPM) values for *CrMYB96* and *96b* extracted from transcriptome data of macro-dissected stems of *C. roseus* (see Methods), showing that these transcripts are more abundant in the epidermis-enriched peel.

(c) Promoter transactivation assays in *N. tabacum* protoplasts by measuring luciferase activity upon co-transfection with constructs containing promoter fragments fused to *fLUC* and constructs for overexpression of Cr*MYB96/b* or *GUS* as a control. The y-axis shows fold change in normalized fLUC activity relative to the GUS control. The error bars depict SEM of four biological replicates. Upper panel: columns labelled with different letters represent statistically significant differences ($P<0.05$, ANOVA with Tukey's correction for multiple comparisons). Lower panel: MYB96 was tested for transactivation of other vindoline pathway promoters; for every promoter statistical significance was determined by the Student's *t*-test (*$P < 0.05$, **$P < 0.01$, ***$P < 0.001$) compared to GUS control.

(d) – (h) Transient overexpression of *CrMYB96* and *96b* in *C. roseus* flower petals.

(d) Overexpression level and MIA biosynthesis gene expression levels were measured by qPCR and are expressed as average log2 FC relative to the *p35S::GUS* control. For each gene, different letters represent statistically significant differences ($P<0.05$, ANOVA with Tukey's correction for multiple comparisons). In this experiment, overexpression of *CrMYB96* (or combination with *CrMYB96b*) leads to higher up-regulation of *T3R* than overexpression of *CrMYB96b* alone; therefore CrMYB96 was used in further experiments. Moreover, an ortholog of the cuticular wax gene *CER1* was also up-regulated, suggesting that the role in cuticular wax biosynthesis regulation is evolutionary conserved.

(e) PCA plot of metabolite profile data.

(f) Loading plot of PCA showing selected MIAs identified in previous studies (blue) and in this study (red).

(g) MIA levels are expressed as log2 FC compared to the *p35S::GUS* control. Total ion current (TIC) values from this experiment including statistical analysis can be found in Supplementary Table S9.

(h) TIC values for vindoline, vindorosine and catharanthine. Columns labelled with different letters represent statistically significant differences ($P<0.05$, ANOVA with Tukey's correction for multiple comparisons).

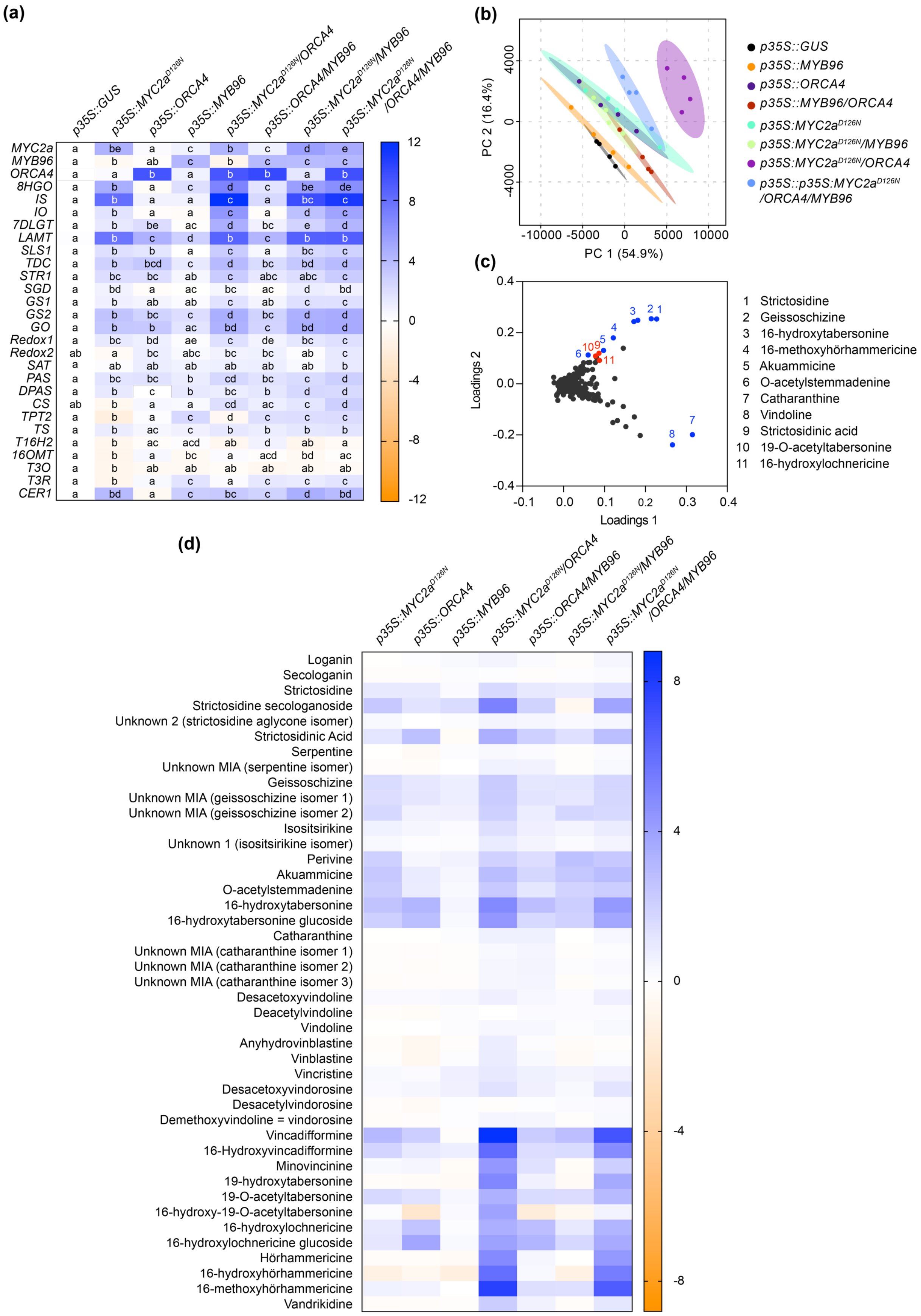

(a)
p35S::GUS
p35S::MYC2a^D126N
p35S::ORCA4
p35S::MYB96
p35S::MYC2a^D126N/ORCA4
p35S::ORCA4/MYB96
p35S::MYC2a^D126N/MYB96
p35S::MYC2a^D126N /ORCA4/MYB96
MYC2a a be a c b c d e
MYB96 a b ab c b c c c
ORCA4 a a b a b b a b
8HGO a b a c d c be de
IS a b a a c a bc c
IO a b a a c a d c
7DLGT a b be ac d bc e d
LAMT a b c d b c b b
SLS1 a b b a c a bc c
TDC a b bcd c d bc bd d
STR1 a bc bc ab c abc abc c
SGD a bd a ac bc ac d bd
GS1 a b ab ab c ab c c
GS2 a b bc c d bc d d
GO a b b ac bd c bd d
Redox1 a bc bd ae c de bc c
Redox2 ab a bc abc bc bc ab c
SAT a ab ab ab ab ab ab b
PAS a bc bc b cd bc c d
DPAS a b c b bc b d d
CS ab b a a cd ac c d
TPT2 a b a c d c c c
TS a b ac c b b b b
T16H2 a b ac acd ab d ab a
16OMT a b a bc a acd bd ac
T3O a b ab ab ab ab ab ab
T3R a b a c a c c c
CER1 a bd a c bc c d bd
12
8
4
0
-4
-8
-12
(b)
PC 2 (16.4%)
PC 1 (54.9%)
4000
0
-4000
-10000 -5000 0 5000 10000
p35S::GUS
p35S::MYB96
p35S::ORCA4
p35S::MYB96/ORCA4
p35S:MYC2a^D126N
p35S:MYC2a^D126N/MYB96
p35S:MYC2a^D126N/ORCA4
p35S::p35S:MYC2a^D126N /ORCA4/MYB96
(c)
Loadings 2
Loadings 1
0.4
0.2
0.0
-0.2
-0.4
-0.1 0.0 0.1 0.2 0.3 0.4
1 Strictosidine
2 Geissoschizine
3 16-hydroxytabersonine
4 16-methoxyhörhammericine
5 Akuammicine
6 O-acetylstemmadenine
7 Catharanthine
8 Vindoline
9 Strictosidinic acid
10 19-O-acetyltabersonine
11 16-hydroxylochnericine
(d)
p35S::MYC2a^D126N
p35S::ORCA4
p35S::MYB96
p35S::MYC2a^D126N/ORCA4
p35S::ORCA4/MYB96
p35S::MYC2a^D126N/MYB96
p35S::MYC2a^D126N /ORCA4/MYB96
Loganin
Secologanin
Strictosidine
Strictosidine secologanoside
Unknown 2 (strictosidine aglycone isomer)
Strictosidinic Acid
Serpentine
Unknown MIA (serpentine isomer)
Geissoschizine
Unknown MIA (geissoschizine isomer 1)
Unknown MIA (geissoschizine isomer 2)
Isositsirikine
Unknown 1 (isositsirikine isomer)
Perivine
Akuammicine
O-acetylstemmadenine
16-hydroxytabersonine
16-hydroxytabersonine glucoside
Catharanthine
Unknown MIA (catharanthine isomer 1)
Unknown MIA (catharanthine isomer 2)
Unknown MIA (catharanthine isomer 3)
Desacetoxyvindoline
Deacetylvindoline
Vindoline
Anyhydrovinblastine
Vinblastine
Vincristine
Desacetoxyvindorosine
Desacetylvindorosine
Demethoxyvindoline = vindorosine
Vincadifformine
16-Hydroxyvincadifformine
Minovincinine
19-hydroxytabersonine
19-O-acetyltabersonine
16-hydroxy-19-O-acetyltabersonine
16-hydroxylochnericine
16-hydroxylochnericine glucoside
Hörhammericine
16-hydroxyhörhammericine
16-methoxyhörhammericine
Vandrikidine
8
4
0
-4
-8

SUPPLEMENTARY FIGURE S11 Combinatorial overexpression of *ORCA4*, *CrMYB96* and *CrMYC2a*^{D126N}.

(a) Gene expression levels were measured by qPCR and are expressed as average log2 FC relative to the *p35S::GUS* control. For each gene, different letters represent statistically significant differences (P<0.05, ANOVA with Tukey's correction for multiple comparisons).

(b) PCA metabolite profile data.

(c) Loading plot of PCA showing selected MIAs previously identified (blue) and in this study (red).

(d) MIA levels are expressed as log2 FC compared to the *p35S::GUS* control. Consistent with other experiments, the MIA profile significantly changes upon overexpression of *ORCA4* or *CrMYC2a*^{D126N} alone or in combination, as opposed to overexpression of *MYB96*. Total ion current (TIC) values from this experiment including statistical analysis can be found in Supplementary Table S10.

SUPPLEMENTARY TABLE S1 List of RNA-Seq datasets used.

Dataset ID (Reference)	Sample ID	Sample name (as in Supporting Dataset 1)	Short sample description
SRA030483 (Gongora-Castillo et al., 2012)	SRR122239	mich_flower	flowers
	SRR122240	mich_culture_AB	Suspension culture (yeast extract (YE) 0.3 mg/mL for 12 hours)
	SRR122241	mich_culture_AC	Suspension culture (YE 0.3 mg/mL for 24 hours)
	SRR122242	mich_culture_AD	Suspension culture control
	SRR122243	mich_seedling_AE	Sterile seedlings
	SRR122244	mich_seedling_AF	Sterile seedlings (Methyl jasmonate(MeJA) 6uM 12 days)
	SRR122245	mich_seedling_AG	Sterile seedlings
	SRR122246	mich_culture_AH	Suspension culture (YE 0.3 mg/mL for 6 hours)
	SRR122247	mich_culture_AI	Suspension culture (MeJA 100uM for 6 hours)
	SRR122248	mich_culture_AJ	Suspension culture (MeJA 100uM for 12 hours)
	SRR122249	mich_culture_AK	Suspension culture (MeJA 100uM for 24 hours)
	SRR122250	mich_culture_AL	Suspension culture
	SRR122251	mich_leaf_AM	Mature leaves
	SRR122252	mich_leaf_AN	Immature leaves
	SRR122253	mich_stem_AO	Stem
	SRR122254	mich_root_AP	Root
	SRR122255	mich_hairy_TDCi_AQ	Hairy roots (TDCi)
	SRR122256	mich_hairy_RebHF_AR	Hairy roots (RebH_F)
	SRR122257	mich_hairy_WT_AS	Wild type (wt) hairy roots
	SRR122258	mich_hairy_WT_AT	Wt hairy roots (MeJA 250uM for o hours)
	SRR122259	mich_hairy_WT_AU	Wt hairy roots (MeJA 250uM for 24 hours)
	SRR122260	mich_hairy_TDCi_AV	TDCi hairy roots (MeJA 250uM for 0 hours)
	SRR122261	mich_hairy_TDCi_AW	TDCi hairy roots (MeJA 250uM for 24 hours)
SRA064724 (Van Moerkercke et al., 2013)	SRS385814	smart_ccCTR	Cell suspension culture (CC) control
	SRS385815	smart_ccMeJA	CC exposed to MeJA
SRP026417 (Van Moerkercke et al., 2015)	SRR924147	smart_ccORCA2	CC overexpressing *ORCA2*
	SRR924148	smart_ccORCA3	CC overexpressing *ORCA3*
SRA064076 (Van Moerkercke et al., 2013)	SRS383709	smart_p24hMeJA	Plants 24 hours MeJA
	SRS383671	smart_p6hCTR	Control plants 6 hours
	SRS383701	smart_p6hMeJA	Plants 6 hours MeJA
ERA669805 (Dugé de Bernonville et al., 2017)	ERR1512369	foli_6h_mse	Plants exposed to *Manduca sexta* (*Ms*) 6 hours
	ERR1512370	foli_6h_ctrl	*Ms* control plants 6 hours
	ERR1512371	foli_8h_mse	*Ms* plants 8 hours
	ERR1512372	foli_8h_ctrl	*Ms* control plants 8 hours
	ERR1512373	foli_24h_mse1	*Ms* plants 24 hours
	ERR1512374	foli_24h_mse2	*Ms* plants 24 hours
	ERR1512375	foli_24h_ctrl	*Ms* control plants 24 hours

	ERR1512376	foli_168h_mse	*Ms* plants 168 hours, new leaves
	ERR1512377	foli_168h_ctrl	*Ms* control plants 168 hours, new leaves
SRP095740 (Pan et al., 2018)	SRR5133632	ET_1-3	Seedlings exposed to ethylene (ET)
	SRR5133633	ET_2-3	
	SRR5133635	ET_3-3	
	SRR5133629	MeJA_2-3	Seedlings exposed to MeJA
	SRR5133628	MeJA_1-3	
	SRR5133636	MeJA_3-3	
	SRR5133634	Control_3-3	Control seedlings
	SRR5133631	Control_2-3	
	SRR5133630	Control_1-3	
	SRR5944780	group_4_1-1	Seedlings
SRP055543 (Van Moerkercke et al., 2015)	SRS858191	GUS_3	Control hairy root lines overexpressing *GUS*
	SRS858189	GUS_4	
	SRS858216	GUS_5	
SRP055543 (Van Moerkercke et al., 2015)	SRS858143	bHLH_14	Hairy root lines overexpressing *BIS1*
	SRS858141	bHLH_18	
	SRS858111	bHLH_19	
PRJEB40213 (This study)	ERR4567494	ORCA3_23	Hairy root lines overexpressing *ORCA3*
	ERR4567495	ORCA3_7	
	ERR4567496	ORCA3_8	
PRJEB40216 (This study)	ERR4567574	S01_WL1	Whole leaves
	ERR4567575	S02_WL2	
	ERR4567576	S03_WL3	
	ERR4567577	S04_CV1	Central vein
	ERR4567578	S05_CV2	
	ERR4567579	S06_CV2	
	ERR4567580	S07_VL1	Leaf without central vein
	ERR4567581	S08_VL2	
	ERR4567582	S09_VL3	
	ERR4567583	S10_WS1	Whole stem
	ERR4567584	S11_WS2	
	ERR4567585	S12_WSa5	
	ERR4567586	S13_PSa6	Peeled stem
	ERR4567587	S14_PS2	
	ERR4567588	S15_PS3	
	ERR4567589	S16_SE1	Stem epidermis
	ERR4567590	S17_SEa6	
	ERR4567591	S18_SE3	
PRJEB40214 (This study)	ERR4567511	S1_CTR1	Mock infiltrated flower petals
	ERR4567512	S2_CTR2	
	ERR4567513	S3_CTR3	
	ERR4567514	S4_AGRO1	*A. tumefaciens* C58C1 infiltrated flower petals

	ERR4567515	S5_AGRO2
	ERR4567516	S6_AGRO3

SUPPLEMENTARY TABLE S2 Oligonucleotide primers used for cloning of coding sequences.

CRO_ID/ Gene or TF family	Sequence fw/ rev
**CRO_T110359**	GGGGACAAGTTTGTACAAAAAAGCAGGCTTAATGGAGTTTTCAGCTACTAATTTTATC
**ORCA5**	GGGGACCACTTTGTACAAGAAAGCTGGGTATTACAATATTGTCTCCTGTTTCATCT
**CRO_T110361**	GGGGACAAGTTTGTACAAAAAAGCAGGCTTAATGCTTGATGAAATTACTATTTCAGCC
**ORCA4**	GGGGACCACTTTGTACAAGAAAGCTGGGTATTAATTTCTTCTCTTCTTTCGTCCCT
**CRO_T110364**	GGGGACAAGTTTGTACAAAAAAGCAGGCTTAATGAATGAAACTTCAAAAGCCTTCCT
**ORCA6**	GGGGACCACTTTGTACAAGAAAGCTGGGTACTATTGTTTACGGCCAAGATGATT
**CRO_T112238**	GGGGACAAGTTTGTACAAAAAAGCAGGCTTAATGGAGTGCTTCAAAAATCATCTTCC
**GATA**	GGGGACCACTTTGTACAAGAAAGCTGGGTACTAAGAATTAGATGGTGGGTGGGA
**CRO_T132285**	GGGGACAAGTTTGTACAAAAAAGCAGGCTTAATGGAGATGGAGAAATCTTGTGA
**BBX**	GGGGACCACTTTGTACAAGAAAGCTGGGTATCACTGAACGTTGGACAAACA
**CRO_T112897**	GGGGACAAGTTTGTACAAAAAAGCAGGCTTAATGTCTTCTTGTTTAACTGGAGGTG
**CCT**	GGGGACCACTTTGTACAAGAAAGCTGGGTACTATCCTTGCTCGCTCTCTGG
**CRO_T127367**	GGGGACAAGTTTGTACAAAAAAGCAGGCTTAATGGTACAATCAAAGAAGTTCAGAGG
**AP2/ERF**	GGGGACCACTTTGTACAAGAAAGCTGGGTATCAATTTCTGTTCAAGAGTTCTTCA
**CRO_T130446**	GGGGACAAGTTTGTACAAAAAAGCAGGCTTAATGGTATCTTCTGATAATAATAAAAAG
**BBX**	GGGGACCACTTTGTACAAGAAAGCTGGGTATCATTTATTTTGCAGGGGGAAAACA
**CRO_T118079**	GGGGACAAGTTTGTACAAAAAAGCAGGCTTAATGTCTCACATAGCAGTGGAAAGA
**bHLH**	GGGGACCACTTTGTACAAGAAAGCTGGGTACTACGGTTCTATTTGTTTGGAGCA
**CRO_T128811**	GGGGACAAGTTTGTACAAAAAAGCAGGCTTAATGGCTGCCAAGGTCAGG
**C2H2 ZF**	GGGGACCACTTTGTACAAGAAAGCTGGGTATTATCTCGACCGGCCTAATCTC
**CRO_T107533**	GGGGACAAGTTTGTACAAAAAAGCAGGCTTAATGGATATGATGACGGATAATTCAGGA
**bHLH**	GGGGACCACTTTGTACAAGAAAGCTGGGTACTACCCAAGAAGTGACCGAAGA
**CRO_T132027**	GGGGACAAGTTTGTACAAAAAAGCAGGCTTAATGTATTGTAATTTTTGGTCAAGTAGT
**bHLH**	GGGGACCACTTTGTACAAGAAAGCTGGGTATCATAGATTGAATATCAAGCCCATCA
**CRO_T109258**	GGGGACAAGTTTGTACAAAAAAGCAGGCTTAATGGAAAGCTTAGATATTCATGAAGAA
**bHLH**	GGGGACCACTTTGTACAAGAAAGCTGGGTATTACATACTCATGGGACTGTCTGG
**CRO_T118542**	GGGGACAAGTTTGTACAAAAAAGCAGGCTTAATGGCTGCCCAAGTTTTAGA
**ARR**	GGGGACCACTTTGTACAAGAAAGCTGGGTATTATGAGAATATCTGATTGTATCTTT
**CRO_T103361**	GGGGACAAGTTTGTACAAAAAAGCAGGCTTAATGATGAAAATGAAGTTACTCAATCGT
**TPR**	GGGGACCACTTTGTACAAGAAAGCTGGGTACTATTGCAGATTATCTACAGTCAAGAG
**CRO_T117719**	GGGGACAAGTTTGTACAAAAAAGCAGGCTTAATGGCTGCTGAATCATCAAGC
**AP2/ERF**	GGGGACCACTTTGTACAAGAAAGCTGGGTATCAGCTGACCCATTTTACTGTGT
**CRO_T133384**	GGGGACAAGTTTGTACAAAAAAGCAGGCTTAATGTCACTGAGGAGAAATTTTCTTT
**TLP**	GGGGACCACTTTGTACAAGAAAGCTGGGTATCACTCGCAGGCCAGTTTT
**CRO_T122239**	GGGGACAAGTTTGTACAAAAAAGCAGGCTTAATGCAAAACCCTTCTCATAAACAT
**NYF-YA**	GGGGACCACTTTGTACAAGAAAGCTGGGTATCATTTTGGTGCACCTCCCT
**CRO_T116828**	GGGGACAAGTTTGTACAAAAAAGCAGGCTTAATGGGTAGTACTAGATTCATGAACA
**WRKY**	GGGGACCACTTTGTACAAGAAAGCTGGGTATCATCCGGTGGTGCCACA

CRO_ID/<br>Gene or TF family	Sequence fw/<br>rev
**CRO_T123350**	GGGGACAAGTTTGTACAAAAAAGCAGGCTTAATGGATCTTTTAGGAGCACATGAGT
**bHLH**	GGGGACCACTTTGTACAAGAAAGCTGGGTATTATTGCATGAGCTTTTGGATGGC
**CRO_T105905**	GGGGACAAGTTTGTACAAAAAAGCAGGCTTAATGGAAGCTCCATCAACTTCTTG
**bHLH**	GGGGACCACTTTGTACAAGAAAGCTGGGTACTATCTGTTTTTCTTTAGTGCTGA
**CRO_T115526**	GGGGACAAGTTTGTACAAAAAAGCAGGCTTAATGGAGAAGAGTAGTACTAGTACT
**ZF**	GGGGACCACTTTGTACAAGAAAGCTGGGTACTAGAGATGAAGGTCTAAACTCACA
**CRO_T129607**	GGGGACAAGTTTGTACAAAAAAGCAGGCTTAATGGGGAAGAAAATGAAGCTCC
**OFP**	GGGGACCACTTTGTACAAGAAAGCTGGGTACTAATTTCTCAGACACGAAGAAGAAGA
**CRO_T131857**	GGGGACAAGTTTGTACAAAAAAGCAGGCTTAATGTACGAAAACGGTGATTTTGA
**AP2/ERF**	GGGGACCACTTTGTACAAGAAAGCTGGGTATCACCATTCACTAAACAATCTCTGT
**CRO_T134559**	GGGGACAAGTTTGTACAAAAAAGCAGGCTTAATGAAGAAAATGATGAGAAGTCAACT
**Homeodomain**	GGGGACCACTTTGTACAAGAAAGCTGGGTATTAGCTAATTTGACTACAAAGTTGATT
**CRO_T116348**	GGGGACAAGTTTGTACAAAAAAGCAGGCTTAATGGATAATTGTGAAATTAGCGAAAGA
**HF**	GGGGACCACTTTGTACAAGAAAGCTGGGTATTATTTGCACGATTGAAAGAGAAAAA
**CRO_T100680**	GGGGACAAGTTTGTACAAAAAAGCAGGCTTAATGAAAGAGAGACAACGGTGGC
**MYB**	GGGGACCACTTTGTACAAGAAAGCTGGGTACTAATTAGGCTCATCCATTCGAGG
**CRO_T101037**	GGGGACAAGTTTGTACAAAAAAGCAGGCTTAATGGTCAAATTTTCCAAGTTT
**TCP**	GGGGACCACTTTGTACAAGAAAGCTGGGTACTAATTTTGAAAATGGATTTGATTTTG
**CRO_T110868**	GGGGACAAGTTTGTACAAAAAAGCAGGCTTAATGGACTCTAAGAAGCTTGTTAATGG
**AP2/B3**	GGGGACCACTTTGTACAAGAAAGCTGGGTACTACCTACAAGTGACACCCTTGC
**CRO_T126752**	GGGGACAAGTTTGTACAAAAAAGCAGGCTTAATGATCTCAAAAGGGTCGTCGT
**ANAC**	GGGGACCACTTTGTACAAGAAAGCTGGGTATTACTGAGCACAAATTAAGGTTCCA
**CRO_T134504**	GGGGACAAGTTTGTACAAAAAAGCAGGCTTAATGGGTAGAAAGTGCTCACATTG
**MYB**	GGGGACCACTTTGTACAAGAAAGCTGGGTATCAGATGACGCTGATAATTGGTC
**CRO_T127378**	GGGGACAAGTTTGTACAAAAAAGCAGGCTTAATGGCTGCAGAGTTGCAATT
**ANAC**	GGGGACCACTTTGTACAAGAAAGCTGGGTATTAAAATGGCTTCTGCAAGAACA
**CRO_T110248**	GGGGACAAGTTTGTACAAAAAAGCAGGCTCGATGTTATCGAGAGTTAACAGCATGG
**bHLH**	GGGGACCACTTTGTACAAGAAAGCTGGGTTTAAACCATCCCTTGGAAGCC
**CRO_T124980**	GGGGACAAGTTTGTACAAAAAAGCAGGCTCGATGGCTTTAGAAGCCCTTTCTTCC
**bHLH**	GGGGACCACTTTGTACAAGAAAGCTGGGTTCAACAAGTACGGGGTGGC
**CRO_T110360**	GGGGACAAGTTTGTACAAAAAAGCAGGCTTAATGTCCGAAGAAATCATTTCCGT
**ORCA3**	GGGGACCACTTTGTACAAGAAAGCTGGGTATTAATATCGTCTCTTCTTCCTTCCTCC
**CRO_T110360**	GATTGGAACCGGTATAGAGGCGTTAGACGGCGG
**ORCA3^{K101R}**	CCGCCGTCTAACGCCTCTATACCGGTTCCAATC
**CRO_T110360**	GTTAGACGGCGGCTTTGGGGGAAGTTCG
**ORCA3^{P107L}**	CGAACTTCCCCCAAAGCCGCCGTCTAAC
**CRO_T110360**	GCCGTGGGGGAGATTCGCGGCGGAG
**ORCA3^{K110R}**	CTCCGCCGCGAATCTCCCCCACGGC
**CRO_T110360**	GCCGTGGGGGACTTTCGCGGCGGAG
**ORCA3^{K110T}**	CTCCGCCGCGAAAGTCCCCCACGGC
**CRO_T110360**	CGGCGGAGATAAGGAATCCGAAAAAGAAAG
**ORCA3^{D117N}**	CTTTCTTTTTCGGATTCCTTATCTCCGCCG
**CRO_T110360/1**	GAGGAGGTTGTTCGAAGGATTGGAACCGGTACAGAGGCGTTAGACGG
**ORCA3/4 swap**	CCGTCTAACGCCTCTGTACCGGTTCCAATCCTTCGAACAACCTCCTC

CRO_ID/ Gene or TF family	Sequence fw/ rev
**CRO_T110360/1**	CGCCGTCTAACGCCCTTATACCGCTTCCATTCCTCAGAATTTTCTTCACTAC
**ORCA4/3 swap**	GTAGTGAAGAAAATTCTGAGGAATGGAAGCGGTATAAGGGCGTTAGACGGCG
**CRO_T131234**	GGGGACAAGTTTGTACAAAAAAGCAGGCTCCATGGGAAGACCACCTTGCTGTGATA
**MYB96**	GGGGACCACTTTGTACAAGAAAGCTGGGTGTCMGAACAAATCAGTAGTTTCCCCT
**CRO_T137796**	GGGGACAAGTTTGTACAAAAAAGCAGGCTTAATGGGGAGACCTCCTTGCT
**MYB96b**	GGGGACCACTTTGTACAAGAAAGCTGGGTACTAAAACAAATCTTCATCATGATCAAA
**CRO_T110365**	GGGGACAAGTTTGTACAAAAAAGCAGGCTccATGTATCAATCAAATGCCCATAATTCCG
**ORCA2**	GGGGACCACTTTGTACAAGAAAGCTGGGTgTCMTTGAGGACGAAGATGACACGATGAA

SUPPLEMENTARY TABLE S3 Oligonucleotide primers used for cloning of promoter fragments.

CRO_ID/ Gene or TF family	Sequence fw/ rev	Length -ATG (0)
**pT3O**	GGGGACAAGTTTGTACAAAAAAGCAGGCTTAGAAACGATAGATTTCTTTTGATGCCT	-1982
**CRO_T113994**	GGGGACCACTTTGTACAAGAAAGCTGGGTAAATCAAAGCTTACTAATTATGAATCTC	0
**pT3R**	GGGGACAAGTTTGTACAAAAAAGCAGGCTTAAGGCTTCCTTTCTTTTGTTGTTTC	-1992
**CRO_T124298**	GGGGACCACTTTGTACAAGAAAGCTGGGTATAAGGAAAAGACAAGCAAAGCCA	0
**pD4H**	GGGGACAAGTTTGTACAAAAAAGCAGGCTTAGATCTTATCTCCTAAACCCTAAACC	-1241
**CRO_T127167**	GGGGACCACTTTGTACAAGAAAGCTGGGTATAGGATTACAGACAGCGGAC	-79
**pDAT**	GGGGACAAGTTTGTACAAAAAAGCAGGCTTAGCATCCAACCATAAAACATTACCCT	-934
**CRO_T120021**	GGGGACCACTTTGTACAAGAAAGCTGGGTAATTCAGACCCCAACACACAAC	-17
**pGO**	GGGGACAAGTTTGTACAAAAAAGCAGGCTTATAATTTTTTATATGCTCGAGTACACT	-2000
**CRO_T127440**	GGGGACCACTTTGTACAAGAAAGCTGGGTATGTGTTTTGTTCAAGAGAGGAGAC	0
**pTAT**	GGGGACAAGTTTGTACAAAAAAGCAGGCTTATGATAAATAATTCTTTCGCTTTTGCA	-2000
**CRO_T120026**	GGGGACCACTTTGTACAAGAAAGCTGGGTATTTGCTCATGCCATTATTATGAACTCA	0
**pMAT**	GGGGACAAGTTTGTACAAAAAAGCAGGCTTAAAAGTAATTGTTATCAATTTATTGA	-2000
**CRO_T120028**	GGGGACCACTTTGTACAAGAAAGCTGGGTATTTGCTCAATATGCTGCTTTCCA	0

SUPPLEMENTARY TABLE S4 List of Genebank IDs of all cloned TFs and promoter fragments.

Genebank accession	CRO_ID	Name
MT414967	CRO_T127167	pD4H
MT414968	CRO_T120021	pDAT
MT414969	CRO_T127440	pGO
MT414970	CRO_T120028	pMAT
MT414971	CRO_T113994	pT3O
MT414972	CRO_T124298	pT3R
MT414973	CRO_T120026	pTAT
MT414974	CRO_T100680	MYB type TF
MT414975	CRO_T101037	TCP type TF
MT414976	CRO_T103361	TPR type TF
MT414977	CRO_T105905	bHLH type TF

MT414978	CRO_T107533	BIS3
MT414979	CRO_T109258	bHLH type TF
MT414980	CRO_T110248	bHLH type TF
MT414981	CRO_T110359	ORCA5
MT414982	CRO_T110361	ORCA4
MT414983	CRO_T110364	ORCA6
MT414984	CRO_T110868	AP2/B3 type TF
MT414985	CRO_T112238	GATA type TF
MT414986	CRO_T112897	CCT type TF
MT414987	CRO_T115526	ZF type TF
MT414988	CRO_T116348	HF type TF
MT414989	CRO_T116828	WRKY type TF
MT414990	CRO_T117719	AP2/ERF type TF
MT414991	CRO_T118079	bHLH type TF
MT414992	CRO_T118542	ARR type TF
MT414993	CRO_T122239	NYF-YA type TF
MT414994	CRO_T123350	bHLH type TF
MT414995	CRO_T124980	bHLH type TF
MT414996	CRO_T126752	ANAC type TF
MT414997	CRO_T127367	AP2/ERF type TF
MT414998	CRO_T127378	ANAC type TF
MT414999	CRO_T128811	C2H2 ZF type TF
MT415000	CRO_T129607	OFP type TF
MT415001	CRO_T130446	BBX type TF
MT415002	CRO_T131234	MYB96
MT415003	CRO_T131857	AP2/ERF type TF
MT415004	CRO_T132027	bHLH type TF
MT415005	CRO_T132285	BBX type TF
MT415006	CRO_T133384	TLP type TF
MT415007	CRO_T134504	MYB type TF
MT415008	CRO_T134559	Homeodomain type TF
MT415009	CRO_T137796	MYB96b
AJ238740	CRO_T110365	ORCA2
AJ251249	CRO_T110360	ORCA3

SUPPLEMENTARY TABLE S5 Oligonucleotide primers used for qPCR

Gene name or TF family name	Gene ID	fw	rev
**GES**	CRO_T119458	GGGCAAGGTGTCACTGAAGA	CCCAAATCATCCCAAAGGCG
**G8O**	CRO_T133061	TGCTTTGGCATTCAAACCCG	GCAAATTCTTCGGCCAGCAC
**8HGO**	CRO_T107879	GCATTCCTGGACACGAAGGA	GCATTCCCCACATTCTCCCA
**IS**	CRO_T130026	AGTAGTAGGAGTCACCGGCA	CTTGCCACGCCGTATACCTT
**IO**	CRO_T138994	TCAAGTCCAAATACGGGCCG	CGGCGGAAACTGCATTTTGA
**7DLGT**	CRO_T106494	GCAGAGGGAGTTCTCAAGGC	TGGCCAATGCACATTCTTGG

Gene name or TF family name	Gene ID	fw	rev
**7DLH**	CRO_T131714	GGTCTTGAATCACCCTGCCA	TGGGACACCAGCCACAATAC
**LAMT**	CRO_T103723	TGGAAGCCCACCCAATGAAA	ACTGCCTTGGCTGCATCAAT
**SLS1**	CRO_T113655	CCCTGCAGGAACACAAGTGA	GCATTGGCAACTCCATCAGC
**SLS2**	CRO_T109472	CCACTGGAGTTTTGCTCACA	TTATTCCTGCCAAAGGCTTC
**TSB**	CRO_T127328	TGTGTCGGTGGTGGTTCAAA	CCAAAACCAGCAGCTTCCAC
**TDC**	CRO_T125328	GGTCGCTGAAACTTTGGCTC	ATTTTGCCCATTGCGACGTC
**STR1**	CRO_T125329	CGTCCAAGATGGCCGAGTTA	CTGGATCGGTGCTGTTCTCA
**SGD**	CRO_T111319*	GGATGGAGCCTCTCAATGAA	CACCCGTTGTTAATGGCTCT
**GS1**	CRO_T113154	TCAAGGCGTAAGGAGAAGGA	AGTACCTGCCAGAGCCTTCA
**GS2**	CRO_T113153	GGTCTTGGTTCCATTGCTGT	TGCTCTTCAATGGCTTCCTT
**GO**	CRO_T127440	TCCTTTGATGCACGTGAAAA	TTACTGACCGATCTGCAACG
**Redox1**	CRO_T129272	GAAGTGACGGAAGTGGGGAACAAA	TCGCATTCGCCACATGAGTCAA
**Redox2**	CRO_T132421	TCGCTTGGGGAAGTAATGCTGT	TGAGACTTGCTCCTTGCTCGTA
**SAT**	CRO_T109653	GGATGGGGAAAGCCTGTTTCTGTT	CTTCAGCCATGCTGATCCATGCTT
**PAS**	CRO_T113148	CTTCACTCCCATGTCCAATCT	CGATAGGATAAGCCCTCGTAATC
**DPAS**	CRO_T129267	GAAATAGCGGCATCGACAAAC	GCTGGGAGTGGTGCTAATAA
**TS**	CRO_T110304	TGCTCCTGGTGGAAATGATAACCC	AATCAGCAACCTCGAGCAACCA
**T16H1**	CRO_T110599	AGGCTTCATCCACCAGTTCC	CCTTCCGATAGCCCATGCAT
**T16H2**	CRO_T110598	GATCAACTCACAGTGGCAGTC	GACTTGAGGACTTGTGATTGGC
**16OMT**	CRO_T110596	CTCTTGTGCCCCCAGTTCAC	CGACTTGATGGGGTAAGGGA
**T3O**	CRO_T113994	CCCATGTGATGAGCAAAGCG	AACAATGGAGCAGGAGGGTG
**T3R**	CRO_T124298	GAAGGGCTACAGGGGAACAC	CACCCACAATTTCATGCCCG
**T19H**	CRO_T119486	TGAGTTGCCAAATGGAGTCA	CAAACGAGAGAGGGTTTTGG
**TAT**	CRO_T120026	CCACAGACTTGGTCCTTCCC	ATGACTGAGCTGCACGATCA
**MAT**	CRO_T120028	ATCGAAGGCCATTGAGTTTG	GCTGCTGATTTCCCTGCTAC
**TEX1**	CRO_T122015	AGGAAGGCGTATTTGTCCGG	TCTTCTGGCTTCATTCCGCC
**TEX2**	CRO_T120417	CCGTTGGCGCAACTTTTGTA	ACCCAAAATCTCCGCCATGT
**THAS1**	CRO_T113666	TGTGGCAAATGTGAAATGTGT	ATTTGAACATGCCCCGTAAT
**AS**	CRO_T116107	GAATCGGCCAACTTTCAGCC	AGTAGTTTGTGGCCTGTCCG
**HYS**	CRO_T140758	AAGTTGGTGTAGGGGGCTTC	CAAAATGGCCATCTGTTGATT
**VAS**	CRO_T112618	GTGTTTGTCCAGGGTTTGCC	GCAGCAAAGCGAGTTTCACA
**NMT**	CRO_T111273	TTCGTGAGATGGTTCGGGTG	CGGCGCCGTCACATATTTTT
**CS**	CRO_T139139	TGGGGCTGGCTTTTGTCTAGAATC	TAAGCTGCGGGTAAAAGGTGCTCT
**TPT2**	CRO_T137586	CCAATGTCACCGGTGCATTC	TCCACCTGTTTTCCGTCCAG
**D4H**	CRO_T127167	ACAGCTGATCACGAACGACA	TCTTGGCGAAACCCCTTCTT
**DAT**	CRO_T120021	GGTTTCAATTTATTTCTCACGTAC	AACTATCAGAAAGGTAAGCATCGA
**PRX1**	CRO_T141131	TAACGGGGAATCAAGGTGAA	AATTTCAGCAGCCTCTTCCA
**V19H**	CRO_T135744	GCTCAGAAACATGGGCCTCT	TTGGCCATTTCGGGTGATGA
**BIS1**	CRO_T107535	ATGGAATCAGTGGTGCTAGTGA	TTCAATTTCAGGGAGCTGTGAC
**BIS2**	CRO_T107539	TGTGCAGTTCTTGGTCAAGACT	AGTGGAATTTGGATCCAAGTTTG
**MYC2a**	CRO_T124533	AGTGGTGAAGGAGGCAGAGA	ATGGCTCTTCCCTTCCATTT
**N2227**	CRO_T133527/8*	GGTTGCTCTTCATTACGGATTT	TGCAGCATAGTAATGGTTTTGC
**SAND**	CRO_T130722	CAGTTCCACAATGCTTTCTGAC	GGGACTGATCAATCGAAGTAGC

Gene name or TF family name	Gene ID	fw	rev
**NPF2.4**	CRO_T131105	ACAAATATTGGATGAGCCCAAG	CCCTGTTTTTATTCTTGGAAGC
**NPF2.5**	CRO_T131100	AAAGAATGGGAATTGGAATGG	TTCGTGGCTCAAAGCCTAAT
**NPF2.6**	CRO_T131101	CGATTATCGATCAATTGCAAGG	CTTCTGAGGTACCCAAGATTACG
**NPF2.9**	CRO_T105710	CCTCCTCCATTTTCATTTTCTG	GATGCGGTTGGATTTGTGAGTC
**ORCA2**	CRO_T110365	GTTGCGGGAGAACAAGAAGA	AACGCCACGGTACCTAATCC
**ORCA3**	CRO_T110360	CGGAAAGCTGTCAGGAGGAT	CGTCTAACGCCCTTATACCGG
**ORCA3 alt**	CRO_T110360	CGGAAAGCTGTCAGGAGGAT	TCGTATGTACCCAACCAAATCC
**ORCA4**	CRO_T110361	TCAGCCTCCGATCTAGCTCT	GATTCGAGCTGCTGTCGTCA
**ORCA5**	CRO_T110359	CCACGGAGGCTAATAGGGGA	TTCTTCTTCCCGGCCATGTC
**ORCA6**	CRO_T110364	TTATTATTAATTCCCCCAAATGCAGGT	TGCTAATGCAGTCAACTCTTGT
**CER1**	CRO_T110442	GCTCGCTCCTTCATCTCTCC	CCTGCTACTCTGCTTGCACT
**BIS3**	CRO_T107533	TCCTCATGATTAACAATGATGATGAA	TTGACCAACAGTTGCTGCAC
**GATA**	CRO_T112238	GAGGAGGGGGATATGGCTCT	GGGGGTGTAGTTTTGGTGGT
**BBX**	CRO_T132285	CACATTCTGCTAATCGCGGC	ACAACGAGGTTCCTTGCTGT
**CCT**	CRO_T112897	ACTCTCCGACTTCAGAGGCT	TTCCCCCGCTTTCTGAGAAC
**AP2/ERF**	CRO_T127367	GGGCCTCGTTCTGATTCCAA	GGTTGCTGCGACGTTTGATT
**BBX**	CRO_T130446	TATGGGCGCGGTATTCAACA	CATGCACAGTGTCCTGCAAC
**bHLH**	CRO_T118079	GGCTTGAATGTCAGCTGAGC	TGGAGCAAACCACTTGTTCTG
**C2H2 ZF**	CRO_T128811	TGGCAACATCACTTGGCCTC	GCGGTGGACATTCATATGGC
**bHLH**	CRO_T132027	AAGGAAGAAGGCGTCGTCAA	AGAGCAGCCATTTCTTGCCT
**bHLH**	CRO_T109258	ACGGACACAATGGTAAAGCT	GGCTACCTGAGAAAGCAGTGA
**ARR**	CRO_T118542	TGGGTTTGCTGGATGAGGAA	AGCCAGTCATTCCAGGCATG
**TPR**	CRO_T103361	CGGTCAGCAGAAGAAGCAGA	TCAACCCACTTCTCCAGCAC
**AP2/ERF**	CRO_T117719	ATGAGCGAATCGACGAGGAC	CGGACGAGTCGTAGCAACTT
**TLP**	CRO_T133384	CTTACCCCGCTGTTTCTGGT	TCACTGATGCCACTGTCACC
**NYF-YA**	CRO_T122239	TACGAGCATCTTCAGTGGCG	TCCAACATGCGCGTCCATAT
**WRKY**	CRO_T116828	CAAGGGAGAGATGGGTGCTC	TGGATAGGGGGAGCCTTTGA
**bHLH**	CRO_T123350	GTCTACCATGGTTCCTGGCC	TGCGTTCTTGGAGCTGTTCT
**bHLH**	CRO_T105905	TGCAGCAAAAATGGCACCAA	GACTGAGCTGCTCCCTTCTC
**ZF**	CRO_T115526	CCAAAACCACCAACACCACC	CGGAATTCTCCACAACCCCA
**OFP**	CRO_T129607	TGGAAGCCCCAAAACCCTTT	TCCGGAAGAGGGTCAATCGA
**AP2/ERF**	CRO_T131857	GAGATCCAGCGAAGAACGGA	CCGATAAGCTGCTCGATCGT
**Homeodomain**	CRO_T134559	AGGATGGTGGAATTGGCTGG	GCCACTTCCACCAATGCATC
**HF**	CRO_T116348	TCCACGAGAACAAACGCCTC	CAGAACTAGAGTGGGCAGCC
**MYB**	CRO_T100680	CAACCTGACCGAAGCAGTCT	TGCAGCATTCCAGAAGCTCA
**TCP**	CRO_T101037	CCGCATTGTTCGATCCACAG	CTCACCCGTCGATCTCTTGG
**AP2/B3**	CRO_T110868	CGCCCCTTTTTGTATCGACG	CCTTCCAGCAATCGCCTTCT
**ANAC**	CRO_T126752	CGTGACCCTTTCTTTTGGCG	TGTTGGGGAGACTGCCTTTC
**MYB**	CRO_T134504	CACAAGAACGCCAACCCAAG	TGGAACGCCTCTTGAGCTTT
**ANAC**	CRO_T127378	GGAAATGTGCGTCTCAACCG	CGCCATACCAGGAAGATCCC
**bHLH**	CRO_T110248	CGGGTTTGGAGAGGGTTCAG	CCTCAGTGCAGCCCTCTTTT
**bHLH**	CRO_T124980	TTCAGCGGATGACATAGCGG	TAGCCCTCCTCCTCCTCCTA
**MYB96**	CRO_T131234	AATGATCAGCAAGGAGGCGG	AATTTACAGCTGCTTCATCCACA

Gene name or TF family name	Gene ID	fw	rev
**MYB96b**	CRO_T137796	TGCATCTAAAGGCCAGTGGG	CCAGTGTCAAGGCATCCTGT
**GUN4**	CRO_T139058	GCCATCCTGCTTTTGAAGGC	GCAGCAGATAAACCCCCACT
**CHLI1**	CRO_T103461	ATTTCACATCCTGCCCGGTT	TGTGCATGCATTCCAAACCG
**GLU1**	CRO_T100999	ATTTTGCCCTGAGGATGCCA	CACAGCAAAACGTTCCCCAG
**PLT6**	CRO_T121364	GCACAAGGGGCTTCAATTGG	TAGCTCCCGCCAATGACAAG

*ID not correct in Reference Genome, manually corrected based on other transcriptomes.

SUPPLEMENTARY TABLES S6-S10 MIA levels of different flower petal infiltration experiments as indicated. Values labelled with different letters indicate statistically significant differences (P < 0.05 calculated by ANOVA with Tukey's correction for multiple comparisons.

SUPPLEMENTARY TABLE S6 MIA levels upon overexpression of *ORCAs*.

	p35S::GUS ± SEM		*p35S::ORCA2* ± SEM		p35S::ORCA3 ± SEM		*p35S::ORCA4* ± SEM		*p35S::ORCA5* ± SEM		*p35S::ORCA6* ± SEM	
Loganin	17625.38 ± 2167.60	a	16810.58 ± 1795.08	a	17077.22 ± 2058.43	a	15206.09 ± 1843.48	a	15801.42 ± 518.64	a	18963.89 ± 922.95	a
Secologanin	171180.14 ± 14499.32	a	168473.28 ± 16717.87	a	179176.07 ± 8601.95	a	142409.56 ± 9877.43	a	171930.23 ± 10586.70	a	184295.65 ± 5698.73	a
Strictosidine	3258939.47 ± 269524.31	a	3893459.39 ± 390430.99	ab	4310460.85 ± 243889.15	ab	5367515.97 ± 589468.00	b	3268640.37 ± 262797.49	a	2851583.99 ± 167540.26	a
Strictosidine secologanoside	n.d.	a	143.26 ± 100.39	a (n.d in 50%)	n.d.	a	1180.63 ± 504.45	b	n.d.	a	27.83 ± 27.83	a (n.d. in 75%)
Unknown 2 (strictosidine aglycone isomer)	61474.47 ± 2087.38	a	87104.91 ± 8849.05	ab	115825.82 ± 10617.37	b	68817.73 ± 10988.09	a	73601.77 ± 6369.61	a	84033.37 ± 5165.24	ab
Strictosidinic Acid	281585.82 ± 21757.76	a	496624.55 ± 69389.41	a	1276110.50 ± 133520.65	b	1151321.40 ± 242493.25	b	422227.44 ± 35179.04	a	318509.81 ± 17158.80	a
Serpentine	2324902.62 ± 197161.98	a	1982141.11 ± 208396.21	a	2475673.54 ± 195546.70	a	2077741.85 ± 534684.92	a	2339104.21 ± 545604.89	a	2298310.64 ± 117079.01	a
Unknown MIA (serpentine isomer)	647187.00 ± 17487.69	a	661450.03 ± 64300.37	a	738198.07 ± 50320.83	a	735765.70 ± 177241.83	a	571635.81 ± 130560.67	a	740626.38 ± 57117.53	a
Geissoschizine	484273.85 ± 56276.89	a	1255979.65 ± 126167.58	b	2543671.29 ± 208974.63	c	852073.67 ± 142919.64	ab	965583.71 ± 93937.07	ab	868932.16 ± 44977.13	ab
Unknown MIA (geissoschizine isomer 1)	1004629.00 ± 107253.68	a	3021401.96 ± 297899.88	b	6758134.73 ± 574717.66	c	1881703.04 ± 360679.97	ab	2393413.70 ± 242716.16	ab	2077270.86 ± 116204.00	ab
Unknown MIA (geissoschizine isomer 2)	59467.49 ± 6427.65	a	120020.73 ± 15061.36	bc	155534.40 ± 11283.00	c	83274.46 ± 19253.96	ab	52809.09 ± 3870.09	a	65797.87 ± 7522.57	a
Unknown 1 (isositsirikine isomer)	313491.34 ± 13274.63	a	425925.05 ± 43974.76	a	680951.70 ± 64148.28	b	438919.85 ± 68368.05	a	401676.62 ± 36671.16	a	426616.49 ± 16766.70	a
Isositsirikine	278013.65 ± 16846.72	a	373626.86 ± 36350.64	a	422133.20 ± 30465.74	a	423987.85 ± 67857.06	a	280666.38 ± 25145.25	a	371290.32 ± 17316.57	a
Perivine	106630.91 ± 4362.20	a	191979.35 ± 19670.21	ab	168940.09 ± 17215.88	ab	231511.27 ± 46723.09	b	125292.97 ± 11991.40	a	147817.61 ± 10341.48	ab
Akuammicine	217149.15 ± 14251.41	a	431327.39 ± 44961.30	b	301704.50 ± 12925.86	ab	494667.49 ± 96705.10	b	212423.58 ± 21861.19	a	267564.80 ± 16382.93	a
O-acetylstemmadenine	285384.26 ± 33981.10	a	396035.90 ± 32906.25	ab	354546.47 ± 12288.51	ab	408649.32 ± 33199.65	b	291303.13 ± 16727.28	a	331319.86 ± 17340.03	ab
16-hydroxytabersonine	236505.11 ± 29528.00	a	980025.80 ± 100088.65	b	709016.29 ± 49945.59	ab	1617549.55 ± 256250.18	c	351625.97 ± 35739.56	a	378565.15 ± 35153.62	a
16-hydroxytabersonine glucoside	21106.33 ± 3070.29	a	59935.16 ± 5721.10	b	56817.49 ± 7688.34	ab	114752.23 ± 18055.98	c	24312.50 ± 2235.73	a	22916.85 ± 2618.79	a
Catharanthine	9502483.18 ± 527452.99	a	10185635.15 ± 954787.04	a	10539400.36 ± 436607.75	a	11204859.70 ± 1504637.03	a	8055529.92 ± 1120877.53	a	11840581.16 ± 854946.78	a
Unknown MIA (catharanthine isomer 1)	5817169.65 ± 261033.71	a	6061133.04 ± 614549.65	a	7119639.59 ± 500279.02	a	6458861.81 ± 954773.77	a	5475778.74 ± 656023.90	a	7510768.69 ± 615668.52	a

Unknown MIA (catharanthine isomer 2)	3651172.35 ± 205625.52	a	3981720.74 ± 405962.48	a	4591785.48 ± 318612.76	a	4171041.45 ± 662515.76	a	3416365.32 ± 389145.48	a	4618904.46 ± 376395.55	a
Unknown MIA (catharanthine isomer 3)	597799.07 ± 11818.06	a	655548.05 ± 68935.66	a	794130.57 ± 68009.74	a	746452.70 ± 111931.08	a	552713.43 ± 62861.96	a	800013.94 ± 52882.85	a
Desacetoxyvindoline	617731.34 ± 87917.14	ab	880009.31 ± 70912.99	b	647521.57 ± 74506.27	ab	606161.19 ± 48638.01	ab	597575.29 ± 16299.77	a	871300.66 ± 43245.92	ab
Deacetylvindoline	299605.96 ± 10440.08	a	320925.21 ± 27968.21	a	331481.32 ± 17608.41	a	278500.01 ± 19193.34	a	285962.34 ± 22800.68	a	367904.07 ± 17380.97	a
Vindoline	9351770.86 ± 777129.99	a	10071645.17 ± 746508.98	a	11196624.74 ± 1053443.15	a	9325684.41 ± 538140.76	a	9458499.26 ± 869042.83	a	11205775.39 ± 641733.96	a
Anhydrovinblastine	315768.67 ± 17360.80	a	382207.33 ± 79352.70	a	391216.31 ± 31360.38	a	313096.30 ± 68086.04	a	243123.80 ± 41568.43	a	381113.21 ± 33507.31	a
Vinblastine	66451.18 ± 9908.89	a	92626.23 ± 18912.39	a	86120.06 ± 8138.94	a	70784.57 ± 22432.29	a	54781.73 ± 11424.54	a	78097.39 ± 20789.89	a
Vincristine	137836.62 ± 11676.38	a	150443.44 ± 15443.39	a	159327.51 ± 6368.79	a	120598.52 ± 26465.96	a	106538.64 ± 14690.10	a	143140.55 ± 15745.80	a
Desacetoxyvindorosine	6304.61 ± 1171.30	a	10647.66 ± 892.74	ab	6832.81 ± 1165.50	a	8631.06 ± 1343.06	ab	7049.38 ± 693.49	a	12712.49 ± 1340.31	b
Desacetylvindorosine	17748.33 ± 1093.51	a	19353.01 ± 1775.73	a	19273.81 ± 1722.43	a	18358.39 ± 2715.84	a	20048.62 ± 2283.52	a	25428.98 ± 1315.57	a
Demethoxyvindoline = vindorosine	433708.03 ± 18099.78	a	504526.26 ± 50526.80	a	534409.28 ± 39973.28	a	521432.17 ± 94984.45	a	416491.63 ± 65335.37	a	619774.63 ± 48058.47	a
Vincadifformine	3510.84 ± 197.70	a	5777.01 ± 899.43	ab	4524.39 ± 531.70	ab	10041.78 ± 2745.91	b	2710.44 ± 771.11	a	5298.57 ± 1163.96	ab
16-Hydroxyvincadifformine	2628.81 ± 276.42	a	7880.72 ± 1207.24	a	17880.15 ± 2712.05	b	4279.05 ± 1487.13	a	5257.53 ± 876.93	a	5117.18 ± 697.47	a
Minovincinine	1537.25 ± 245.75	a	2710.81 ± 292.72	a	5332.97 ± 868.25	b	3413.38 ± 802.19	ab	1274.11 ± 176.90	a	2060.31 ± 180.76	a
19-hydroxytabersonine	40201.69 ± 3409.83	a	57404.81 ± 7499.03	a	56188.74 ± 6908.92	a	49478.63 ± 6201.54	a	41563.00 ± 2818.75	a	56217.78 ± 3284.85	a
19-O-acetyltabersonine	83672.73 ± 7433.12	a	215233.42 ± 16510.63	b	167834.01 ± 12321.07	c	300473.30 ± 35168.08	d	116404.38 ± 9199.83	ac	129030.59 ± 6893.00	ac
16-hydroxy-19-O-acetyltabersonine	41.43 ± 16.00	a	152.11 ± 39.56	a	19.37 ± 12.29	a	55.73 ± 36.25	a	91.94 ± 38.29	a	146.98 ± 53.37	a
16-hydroxylochnericine	181233.31 ± 7502.30	a	530591.48 ± 56142.18	a	384911.78 ± 29794.10	a	1208983.16 ± 212690.52	b	237525.87 ± 26963.13	a	243321.33 ± 11289.52	a
16-hydroxylochnericine glucoside	16762.93 ± 3093.86	a	58792.03 ± 5408.12	a	35975.42 ± 4500.69	a	225106.48 ± 35746.05	b	20360.30 ± 2786.09	a	8507.11 ± 1746.77	a
Hörhammericine	30101.62 ± 1432.96	a	44844.42 ± 4560.43	a	52316.87 ± 5020.81	a	54981.20 ± 11522.39	a	32818.98 ± 2988.17	a	38820.64 ± 1952.01	a
16-hydroxyhörhammericine	71.52 ± 22.88	a	363.28 ± 123.21	ab	870.56 ± 296.20	ab	998.66 ± 364.73	b	115.80 ± 14.86	ab	99.56 ± 27.42	a
16-methoxyhörhammericine	8104.56 ± 582.99	a	29086.91 ± 2857.31	a	117435.00 ± 20099.66	b	16640.37 ± 2720.54	a	42251.69 ± 2803.09	a	12349.81 ± 533.10	a
Vandrikidine	139663.86 ± 4134.40	a	155217.11 ± 16344.87	a	161911.98 ± 10945.14	a	148383.13 ± 26667.18	a	110917.30 ± 14011.74	a	178893.60 ± 13500.38	a

SUPPLEMENTARY TABLE S7 MIA levels upon overexpression of *ORCA3* mutants.

	p35S::GUS **± SEM**		***p35S:ORCA3*** **± SEM**		***p35S::ORCA3***^{K101R} **± SEM**		**p35S::ORCA3**^{P107L} **± SEM**		**p35S::ORCA3**^{K101RP107L} **± SEM**		**p35S::ORCA3**^{K101RK110R} **± SEM**		**p35S::ORCA3**^{K101RK110T} **± SEM**	
Loganin	15870.77 ± 1458.96	ab	12864.96 ± 1192.62	a	14891.98 ± 883.49	ab	19717.96 ± 1211.98	ab	16149.62 ± 843.79	b	16490.56 ± 1042.61	ab	15609.33 ± 1390.84	ab
Secologanin	69445.44 ± 3537.49	a	68481.76 ± 3403.10	a	71583.40 ± 4175.36	a	68643.41 ± 3326.18	a	69928.17 ± 796.63	a	74543.71 ± 3737.52	a	69267.73 ± 2913.20	a
Strictosidine	2491704.50 ± 220688.23	a	2846468.72 ± 148459.61	ab	2905514.79 ± 296430.80	ab	3757294.17 ± 431858.18	b	3182636.10 ± 107286.62	ab	3261658.62 ± 143333.09	ab	3068013.90 ± 170395.59	ab
Strictosidine secologanoside	9.69 ± 9.69	a	39.89 ± 28.45	a	70.19 ± 46.78	a	85.82 ± 41.42	a	65.64 ± 23.14	a	410.58 ± 167.91	b	47.60 ± 47.60	a
Unknown 2 (strictosidine aglycone isomer)	102405.25 ± 13429.58	a	118696.97 ± 8931.98	a	170719.70 ± 11907.70	b	139602.81 ± 14258.81	ab	127566.68 ± 4744.53	ab	172485.40 ± 11443.52	b	129926.43 ± 10934.42	ab
Strictosidinic Acid	38752.78 ± 5734.89	a	346478.86 ± 25775.03	b	693798.12 ± 92274.52	c	451479.73 ± 33725.08	bd	603865.82 ± 41936.16	cd	641522.64 ± 55062.92	cd	257901.12 ± 13609.91	b
Serpentine	2212345.63 ± 374693.27	ab	1675881.74 ± 219310.15	a	3556228.66 ± 780828.03	b	2753236.80 ± 338911.27	ab	2869463.86 ± 189678.55	ab	2597008.84 ± 156549.08	ab	2314128.20 ± 293452.54	ab
Unknown MIA (serpentine isomer)	747125.34 ± 110960.56	ab	597302.38 ± 75465.39	a	1190529.90 ± 198473.19	b	1063275.12 ± 127753.32	ab	917204.08 ± 53889.42	ab	1009392.84 ± 68169.01	ab	870255.96 ± 98521.43	ab
Geissoschizine	1340801.46 ± 201855.89	a	6714390.87 ± 269268.96	be	8590381.24 ± 481830.05	ce	4961214.37 ± 440360.75	bd	4333031.51 ± 140386.18	d	10251251.59 ± 675868.73	c	6172585.80 ± 569228.45	bd
Unknown MIA (geissoschizine isomer 1)	855433.79 ± 131489.70	a	4446439.46 ± 196721.59	bc	5777104.99 ± 342575.90	ce	3398546.65 ± 311671.66	bd	2752421.45 ± 88262.99	d	6892975.47 ± 445481.03	e	4200659.74 ± 411108.28	b
Unknown MIA (geissoschizine isomer 2)	60771.73 ± 13974.72	a	126852.38 ± 9214.37	b	259793.66 ± 14916.38	c	154433.19 ± 19183.68	b	159054.48 ± 6013.53	b	235498.41 ± 8204.45	c	134505.10 ± 17189.43	b
Isositsirikine	225522.02 ± 29611.46	a	259818.09 ± 17884.46	ac	421237.32 ± 34205.50	b	380875.60 ± 44377.34	bc	405217.16 ± 15165.71	b	437540.27 ± 28321.47	b	304004.78 ± 20724.26	a
Unknown 1 (isositsirikine isomer)	372859.26 ± 44916.38	a	502353.99 ± 34064.79	b	811497.66 ± 77791.17	cd	652790.17 ± 73439.55	bcd	586669.12 ± 23184.49	ab	850643.18 ± 55711.20	d	572485.94 ± 46757.00	abc
Perivine	107276.55 ± 14116.95	ac	118061.78 ± 10883.12	ac	226905.69 ± 13662.81	b	176003.82 ± 13725.48	bc	197001.11 ± 8529.24	bc	202893.73 ± 14417.91	b	144921.25 ± 11817.69	c
Akuammicine	182818.89 ± 31162.33	a	226540.97 ± 21275.02	ab	495310.09 ± 29322.20	c	338896.06 ± 32715.98	bd	477632.31 ± 12708.58	c	421485.92 ± 30392.35	cd	253736.88 ± 27036.65	ab
O-acetylstemmadenine	155778.26 ± 16492.71	a	197183.36 ± 7772.73	ab	266716.01 ± 17228.75	b	225984.02 ± 14768.30	ab	255672.33 ± 7109.68	b	272375.70 ± 23637.06	b	242422.61 ± 20731.94	b
16-hydroxytabersonine	139850.08 ± 26678.16	a	473944.09 ± 39684.32	b	820746.25 ± 19538.23	c	1121219.85 ± 80115.30	d	1765345.30 ± 93684.63	e	926990.57 ± 69380.26	cd	482094.73 ± 48926.74	b
16-hydroxytabersonine glucoside	12181.32 ± 2315.99	a	38736.11 ± 4627.69	b	71702.11 ± 7653.54	c	79750.27 ± 4025.20	c	188769.17 ± 9185.22	d	84504.62 ± 5867.19	c	34703.94 ± 2010.77	ab
Catharanthine	7827404.24 ± 1157412.25	ab	6461724.23 ± 603424.79	a	11445467.34 ± 1314038.95	b	10871245.25 ± 1429655.87	ab	9269471.35 ± 404395.32	ab	10169822.82 ± 740937.60	ab	9544521.27 ± 868385.03	ab
Unknown MIA (catharanthine isomer 1)	4675344.47 ± 508500.49	ab	3924921.19 ± 289243.66	b	5972668.89 ± 638619.11	a	6050521.54 ± 495682.65	a	4915187.19 ± 204238.95	ab	5554826.41 ± 392778.00	ab	5159076.44 ± 283324.84	ab

Unknown MIA (catharanthine isomer 2)	3434250.89 ± 418890.54	a	2923654.64 ± 247024.68	a	4195981.83 ± 406126.08	a	4296896.47 ± 381370.45	a	3682735.71 ± 141818.72	a	4061934.51 ± 241342.34	a	3613899.63 ± 222877.46	a
Unknown MIA (catharanthine isomer 3)	779360.78 ± 88000.19	ab	648889.45 ± 56062.97	a	951707.83 ± 90936.85	ab	1032899.35 ± 71090.50	b	812171.66 ± 34075.56	ab	909199.58 ± 56347.59	ab	836293.76 ± 55638.03	ab
Desacetoxyvindoline	873307.73 ± 99443.72	ab	772870.24 ± 49877.56	a	1031084.28 ± 66680.13	ab	1232873.85 ± 142466.49	b	1006326.28 ± 22033.45	ab	1060440.89 ± 46672.71	ab	1021962.40 ± 77824.72	ab
Deacetylvindoline	282304.45 ± 36056.18	a	199391.47 ± 19902.41	a	290925.18 ± 31137.29	a	313968.47 ± 38603.71	a	283389.48 ± 6843.87	a	275346.99 ± 10809.87	a	251926.67 ± 14220.10	a
Vindoline	11412219.14 ± 1402156.05	a	9818761.90 ± 690921.49	a	14008785.58 ± 1136842.77	a	14299655.06 ± 1534231.47	a	12189220.94 ± 267256.00	a	13787601.18 ± 529149.77	a	12030213.36 ± 801941.91	a
Anhydrovinblastine	305391.19 ± 33897.09	ab	162595.95 ± 25510.34	a	599733.78 ± 119951.26	b	446950.70 ± 99067.32	ab	407433.11 ± 35961.87	ab	595111.47 ± 66088.50	b	460606.97 ± 72688.60	ab
Vinblastine	245242.70 ± 27857.46	ab	123628.04 ± 12910.92	a	275905.49 ± 45990.33	ab	289032.11 ± 42902.01	ab	298203.22 ± 22738.05	b	294603.35 ± 27493.79	b	270721.22 ± 54741.81	ab
Vincristine	129534.57 ± 14596.21	ab	102900.24 ± 10925.07	a	159258.96 ± 14970.01	ab	144193.73 ± 19649.85	ab	147159.05 ± 7818.60	ab	170893.80 ± 10760.00	b	136680.70 ± 12330.71	ab
Desacetoxyvindorosine	11547.06 ± 1402.21	a	13557.14 ± 658.16	a	14863.69 ± 1833.83	ab	21372.32 ± 2339.35	b	15057.58 ± 928.01	ab	14106.74 ± 864.10	a	14629.20 ± 1225.25	a
Desacetylvindorosine	17564.11 ± 2199.54	a	11931.95 ± 1376.64	a	15365.79 ± 1212.46	a	16420.60 ± 1459.65	a	16468.11 ± 302.60	a	14751.65 ± 587.90	a	13622.95 ± 584.91	a
Demethoxyvindoline = vindorosine	591352.75 ± 92196.44	ab	459187.72 ± 46216.12	a	753659.92 ± 73507.83	ab	838896.04 ± 87617.59	b	630500.96 ± 22195.72	ab	704585.27 ± 35178.09	ab	639932.25 ± 72269.23	ab
Vincadifformine	909.06 ± 218.94	a	782.79 ± 235.22	ac	3824.19 ± 227.83	b	2455.34 ± 453.71	bc	3630.81 ± 558.72	bd	3294.33 ± 468.95	bd	1719.91 ± 458.28	ac
16-Hydroxyvincadifformine	3740.93 ± 557.65	a	19104.21 ± 1571.42	bc	26752.85 ± 1721.56	cd	14668.58 ± 1947.46	b	12185.37 ± 916.35	b	32663.51 ± 2301.73	d	19068.63 ± 2712.30	bc
Minovincinine	549.12 ± 131.16	a	464.20 ± 49.99	a	1432.51 ± 142.17	bc	1541.26 ± 79.89	b	1242.01 ± 100.75	bc	1296.04 ± 84.49	bc	1012.06 ± 138.30	c
19-hydroxytabersonine	5754.82 ± 1216.82	ab	4104.39 ± 556.15	a	9421.75 ± 1183.57	b	9258.87 ± 770.72	b	7286.51 ± 721.66	ab	8179.48 ± 688.02	ab	8717.72 ± 1125.51	b
19-O-acetyltabersonine	64850.36 ± 7959.88	a	99771.33 ± 5864.43	ace	175342.85 ± 5437.02	bcd	138808.79 ± 10829.95	ce	209987.89 ± 6073.83	d	180903.49 ± 12961.44	bd	114192.10 ± 7599.20	e
16-hydroxy-19-O-acetyltabersonine	67.81 ± 32.99	a	14.01 ± 7.35	a	35.72 ± 21.12	a	211.52 ± 34.48	b	59.06 ± 26.21	a	43.88 ± 25.56	a	108.49 ± 45.56	ab
16-hydroxylochnericine	80527.82 ± 9328.14	a	156266.70 ± 12522.67	ab	787393.21 ± 14979.19	c	248122.97 ± 5842.84	ab	915934.04 ± 57154.44	c	771615.35 ± 73858.75	c	260917.60 ± 31555.44	b
16-hydroxylochnericine glucoside	12595.60 ± 2218.97	a	40952.93 ± 5451.75	a	339628.02 ± 19586.33	b	61257.70 ± 8489.74	a	349901.85 ± 22402.43	b	329299.04 ± 18942.78	b	69562.16 ± 5871.19	a
Hörhammericine	6585.21 ± 1179.33	a	8275.07 ± 1190.44	a	25026.61 ± 2402.81	b	9944.88 ± 742.43	a	9224.02 ± 712.22	a	31158.05 ± 3187.54	b	27232.76 ± 3771.75	b
16-hydroxyhörhammericine	143.34 ± 17.26	a	418.33 ± 44.04	a	3662.33 ± 447.86	b	463.16 ± 238.07	a	602.59 ± 66.16	a	6963.80 ± 789.78	c	6348.52 ± 927.50	c
16-methoxyhörhammericine	7915.35 ± 1058.97	a	97351.04 ± 7232.23	b	132958.96 ± 13980.19	bd	16566.13 ± 1273.76	a	26288.89 ± 1448.74	a	177294.42 ± 16041.10	cd	191384.58 ± 19700.40	c
Vandrikidine	102280.39 ± 18475.32	ab	79259.74 ± 10682.92	a	144809.09 ± 20418.22	ab	156512.31 ± 16583.05	b	113597.73 ± 8984.76	ab	120366.81 ± 9606.25	ab	114911.85 ± 12721.81	ab

SUPPLEMENTARY TABLE S8 MIA levels upon combinatorial overexpression of *CrMYC2a*^{D126N} and *ORCAs*.

	p35S::GUS **± SEM**		***p35S::CrMYC2a***^{D126N} **± SEM**		***p35S::CrMYC2a***^{D126N}**/ *ORCA2*** **± SEM**		***p35S::CrMYC2a***^{D126N}**/*ORCA3*** **± SEM**		***p35S::CrMYC2a***^{D126N}**/ *ORCA4*** **± SEM**		***p35S::CrMYC2a***^{D126N}**/ *ORCA5*** **± SEM**		***p35S::CrMYC2a***^{D126N}**/ *ORCA6*** **± SEM**	
Loganin	8626.83 ± 1039.10	ac	7584.28 ± 799.17	ac	12854.25 ± 791.31	b	11760.92 ± 454.11	ab	11271.50 ± 2170.71	ab	6117.48 ± 793.40	c	10601.46 ± 740.24	abc
Secologanin	78983.28 ± 4724.84	a	77621.66 ± 5088.83	a	89818.44 ± 4344.08	a	90499.88 ± 2547.67	a	85320.19 ± 4098.18	a	73663.00 ± 6000.27	a	75629.66 ± 2121.52	a
Strictosidine	2237933.30 ± 227357.62	a	4749196.11 ± 385463.70	bd	4841076.75 ± 220047.52	bd	6653028.42 ± 345263.64	bc	7252442.05 ± 1092499.36	c	3232112.01 ± 333184.30	ad	3566567.54 ± 371696.25	ad
Strictosidine secologanoside	64.91 ± 38.14	a	519.48 ± 219.62	ab	754.22 ± 290.80	ab	656.86 ± 229.69	ab	2739.79 ± 1262.53	b	81.39 ± 81.39	a	123.62 ± 61.47	a
Unknown 2 (strictosidine aglycone isomer)	93556.02 ± 10753.51	a	157727.33 ± 14473.97	abc	191299.49 ± 11714.30	b	216536.96 ± 15089.17	b	168298.82 ± 21825.60	bc	117386.26 ± 15017.37	c	138837.70 ± 11799.74	c
Strictosidinic Acid	98768.62 ± 20453.62	a	498913.63 ± 56348.38	b	672303.51 ± 59814.35	b	980259.26 ± 70420.12	c	1002079.78 ± 112055.50	d	423561.61 ± 54153.46	b	267124.42 ± 34853.44	ab
Serpentine	1630727.39 ± 393739.56	a	2138763.52 ± 441653.75	a	2444748.14 ± 458244.53	a	2856104.91 ± 557111.68	a	2876271.98 ± 666233.06	a	1290570.91 ± 480503.73	a	1463399.19 ± 126682.43	a
Unknown MIA (serpentine isomer)	552494.43 ± 106460.31	ab	647786.31 ± 117665.42	ab	900296.47 ± 131434.68	a	995167.63 ± 137181.30	ab	958369.30 ± 186734.28	ab	391519.79 ± 112458.42	b	612073.16 ± 43479.30	ab
Geissoschizine	1684870.16 ± 260410.12	a	4656122.57 ± 431585.56	bc	9501071.02 ± 560489.48	c	13470663.44 ± 374367.60	d	5290417.91 ± 711635.26	d	5429048.70 ± 697783.78	bc	5580237.01 ± 372427.90	b
Unknown MIA (geissoschizine isomer 1)	1085340.19 ± 168209.90	a	2760815.32 ± 249803.13	b	6089576.41 ± 349748.99	c	8926383.61 ± 231247.90	d	3321401.52 ± 495045.64	b	3549753.38 ± 476536.66	b	3568594.57 ± 237245.21	b
Unknown MIA (geissoschizine isomer 2)	42488.73 ± 10638.50	a	276082.65 ± 26894.19	bcd	360865.35 ± 39508.28	b	377381.19 ± 15620.14	b	233250.30 ± 34081.94	cd	135202.28 ± 15750.90	ac	247215.81 ± 8607.02	d
Isositsirikine	228517.06 ± 28439.04	a	403108.56 ± 42075.05	ab	579377.20 ± 37935.19	bc	575872.36 ± 37374.66	bc	651175.38 ± 74752.76	c	300657.94 ± 41243.21	a	361984.26 ± 25136.82	a
Unknown 1 (isositsirikine isomer)	374539.78 ± 58952.37	a	562563.80 ± 69036.18	ab	781996.45 ± 67027.10	bc	1005580.86 ± 97544.60	c	731807.25 ± 122872.58	abc	470878.67 ± 78838.86	a	517133.91 ± 20228.66	a
Perivine	85247.92 ± 13341.86	a	480932.83 ± 63318.36	bc	480935.77 ± 42850.18	bc	372181.94 ± 33514.28	b	656746.96 ± 76721.27	c	255415.21 ± 40294.36	ab	405010.52 ± 52917.02	b
Akuammicine	122022.54 ± 16925.89	a	937559.91 ± 137598.51	b	1201065.95 ± 81081.86	bc	773372.22 ± 37980.45	bd	1428134.23 ± 167892.85	c	412781.48 ± 50887.77	ad	818158.19 ± 113077.32	bd
O-acetylstemmadenine	129894.59 ± 16235.56	a	444404.01 ± 30554.41	bc	501219.93 ± 29178.61	b	402740.03 ± 15511.38	bc	428924.91 ± 60737.86	bc	226261.13 ± 26047.47	ad	319438.58 ± 39559.44	cd
16-hydroxytabersonine	69239.25 ± 8369.88	a	643021.06 ± 88175.95	b	1962267.77 ± 59154.15	c	1531087.36 ± 43751.08	c	2746220.62 ± 109314.44	d	614433.51 ± 78918.72	b	792531.63 ± 75621.81	b
16-hydroxytabersonine glucoside	7291.49 ± 963.53	a	81335.81 ± 7017.24	bd	148596.15 ± 10954.46	cd	109070.65 ± 4671.16	d	273654.33 ± 16100.37	e	65262.88 ± 9274.26	b	62013.47 ± 6610.35	b
Catharanthine	6264287.65 ± 858352.19	ab	6304859.74 ± 895297.28	ab	9679809.00 ± 758529.24	a	10121644.32 ± 757858.39	a	9348679.55 ± 1237803.54	a	4383851.24 ± 886201.96	b	6988862.90 ± 499854.43	ab
Unknown MIA (catharanthine isomer 1)	4337315.88 ± 600986.07	a	4502610.17 ± 534354.00	a	6042148.63 ± 667862.27	a	6285607.40 ± 559530.27	a	6068825.67 ± 933979.83	a	3497648.91 ± 615798.46	a	4418897.74 ± 257472.39	a
Unknown MIA (catharanthine isomer 2)	3071725.14 ± 484810.49	a	3632875.97 ± 459108.94	a	4584953.73 ± 395268.04	a	4738077.44 ± 372650.22	a	4815156.61 ± 673990.72	a	2625682.63 ± 524244.26	a	3478928.06 ± 163659.70	a

Unknown MIA (catharanthine isomer 3)	572257.89 ± 75777.88	abc	565097.84 ± 73502.17	ac	892989.29 ± 76012.39	abc	915786.34 ± 72507.02	b	827401.26 ± 107631.52	ab	466230.00 ± 73245.22	c	638512.98 ± 36420.57	abc
Desacetoxyvindoline	689357.99 ± 57824.76	acd	634965.06 ± 66318.19	a	1061816.11 ± 21547.91	bd	1120700.68 ± 21165.97	b	812195.56 ± 82269.64	c	535624.59 ± 33122.94	a	880164.06 ± 31677.99	cd
Deacetylvindoline	280988.98 ± 33955.18	a	278110.15 ± 23312.99	a	278318.03 ± 30193.71	a	282441.47 ± 14883.32	a	300738.03 ± 42002.99	a	150090.30 ± 24836.22	b	214372.52 ± 13842.51	ab
Vindoline	9776321.34 ± 993997.98	ab	10440781.30 ± 1364195.83	ab	13776099.96 ± 968623.88	a	14402922.48 ± 750354.76	a	12529975.05 ± 1555210.34	a	7104481.84 ± 961226.59	b	10650093.45 ± 527464.84	ab
Anhydrovinblastine	141512.73 ± 28527.72	ab	212350.25 ± 29297.84	ab	369626.58 ± 84081.20	a	363866.10 ± 44151.80	a	320329.61 ± 77974.70	ab	120954.11 ± 31181.84	b	273287.35 ± 41493.00	ab
Vinblastine	57351.68 ± 11382.30	a	88021.62 ± 10303.26	ab	179765.81 ± 34719.63	b	174082.78 ± 15575.99	b	140687.98 ± 39458.90	ab	54637.09 ± 11729.77	a	130833.87 ± 20103.27	ab
Vincristine	41640.02 ± 5880.28	ac	52310.38 ± 6827.84	abc	88201.58 ± 10369.81	b	64349.26 ± 8266.85	abc	72704.41 ± 13275.37	ab	33714.36 ± 4243.72	c	57528.26 ± 4473.48	abc
Desacetoxyvindorosine	7585.41 ± 923.01	ac	6360.19 ± 777.65	a	18971.11 ± 2928.11	b	18705.45 ± 1504.89	b	11873.17 ± 1388.42	ac	7259.40 ± 885.11	ac	13714.07 ± 659.78	bc
Desacetylvindorosine	15199.29 ± 1781.37	ab	17362.99 ± 1992.51	a	16882.31 ± 1003.73	a	18259.69 ± 689.93	a	17619.11 ± 1832.13	a	10076.15 ± 1556.73	b	14086.73 ± 605.21	ab
Demethoxyvindoline = vindorosine	386198.50 ± 53653.51	ac	408797.04 ± 65847.67	ac	642038.32 ± 47690.78	ab	694586.44 ± 47929.86	b	542592.84 ± 72814.95	ab	277838.57 ± 45551.37	c	481896.69 ± 29774.34	ac
Vincadifformine	247.03 ± 49.40	a	5889.37 ± 1437.46	a	212848.32 ± 10063.38	b	226325.24 ± 15431.19	b	199138.82 ± 27974.21	b	45207.07 ± 9905.86	a	32964.75 ± 3702.96	a
16-Hydroxyvincadifformine	3500.91 ± 876.26	a	22256.18 ± 3298.01	ae	340014.47 ± 13910.91	b	379815.11 ± 22893.51	b	254677.20 ± 27322.21	c	108279.18 ± 17988.03	d	95435.29 ± 13339.80	de
Minovincinine	627.33 ± 167.81	a	807.99 ± 152.29	a	24469.84 ± 5947.05	b	34428.72 ± 3973.01	c	10253.38 ± 906.42	a	2875.48 ± 648.71	a	1380.17 ± 107.00	a
19-hydroxytabersonine	2099.44 ± 401.36	a	2883.45 ± 389.09	a	119314.69 ± 13517.13	b	377209.41 ± 24740.83	b	104313.19 ± 12347.27	a	23898.89 ± 8481.32	a	4323.11 ± 401.31	a
19-O-acetyltabersonine	55398.90 ± 4662.24	a	220765.33 ± 19790.28	b	452668.22 ± 9803.61	cd	348068.34 ± 12519.35	bc	554879.55 ± 80713.83	d	205184.02 ± 17613.09	b	227463.02 ± 29554.85	b
16-hydroxy-19-O-acetyltabersonine	28.50 ± 8.90	a	19.30 ± 11.68	a	7504.69 ± 815.30	a	131981.66 ± 18961.02	b	7292.73 ± 1029.33	a	4189.18 ± 1508.80	a	49.28 ± 25.15	a
16-hydroxylochnericine	52510.55 ± 6388.69	a	183475.69 ± 21187.12	b	463941.53 ± 18700.75	c	287130.22 ± 11521.42	bc	751298.45 ± 106106.81	d	141498.88 ± 19829.92	ab	141265.42 ± 9050.20	ab
16-hydroxylochnericine glucoside	8980.47 ± 1612.25	a	106775.57 ± 21589.15	bc	95786.88 ± 12130.77	bc	58830.14 ± 1666.06	ac	181341.65 ± 25487.06	d	31050.31 ± 4324.36	a	20284.19 ± 1913.02	a
Hörhammericine	4724.77 ± 996.81	a	5992.97 ± 915.68	a	278470.00 ± 38042.54	bc	374346.28 ± 39153.63	b	185240.87 ± 36359.61	c	63542.74 ± 28403.72	a	6372.98 ± 543.77	a
16-hydroxyhörhammericine	86.72 ± 48.60	a	193.00 ± 18.89	a	10580.30 ± 1703.02	b	26707.28 ± 1398.64	c	24552.49 ± 3524.69	c	3244.90 ± 986.72	ab	174.94 ± 36.97	a
16-methoxyhörhammericine	6127.80 ± 1025.41	a	14570.47 ± 1812.75	a	1455135.43 ± 58409.57	b	1768828.83 ± 60800.49	b	1476769.83 ± 190025.82	b	628559.98 ± 120788.73	c	87278.84 ± 15027.98	a
Vandrikidine	58516.82 ± 8298.02	a	61809.06 ± 8650.40	a	286373.73 ± 13438.47	b	627657.05 ± 36265.94	c	311488.93 ± 31407.43	b	114670.79 ± 20833.02	a	72160.38 ± 6822.54	a

SUPPLEMENTARY TABLE S9 MIA levels upon overexpression of *MYB96/b*.

	p35S::GUS ± SEM		*p35S::MYB96b* ± SEM		*p35S::MYB96* ± SEM		*p35S::MYB96/96b* ± SEM	
Loganin	18077.20 ± 1311.99	a	23202.36 ± 1551.57	ab	28192.15 ± 2957.71	b	21051.04 ± 1060.61	ab
Secologanin	166639.21 ± 11085.70	a	189229.95 ± 10593.10	a	187008.77 ± 15666.49	a	165322.53 ± 9280.56	a
Strictosidine	3249908.79 ± 32900.11	ac	2412417.01 ± 62155.70	b	2734654.41 ± 250541.41	a	3890892.24 ± 217153.69	c
Strictosidine secologanoside	3873.44 ± 358.60	a	110.62 ± 110.62	b	1006.49 ± 912.46	b	280.40 ± 280.40	b
Unknown 2 (strictosidine aglycone isomer)	95098.64 ± 9985.24	a	110953.76 ± 5712.12	a	95959.85 ± 9104.73	a	116455.19 ± 6188.85	a
Strictosidinic Acid	319097.32 ± 30435.43	ab	295479.71 ± 16263.51	a	272663.15 ± 37572.91	a	429114.31 ± 12376.90	b
Serpentine	2934652.60 ± 454437.97	a	2777297.91 ± 167880.75	a	3253367.89 ± 513643.97	a	3628148.31 ± 209836.09	a
Unknown MIA (serpentine isomer)	699111.61 ± 54890.41	a	667555.96 ± 41418.47	a	852078.76 ± 117001.48	a	1203419.96 ± 67104.93	b
Geissoschizine	1958540.56 ± 300324.84	a	2108506.58 ± 124252.41	a	1774373.95 ± 219955.12	a	1384011.68 ± 59339.72	a
Unknown MIA (geissoschizine isomer 1)	3517665.90 ± 495131.29	a	4181376.41 ± 278217.33	a	3440154.48 ± 390808.37	a	3122352.57 ± 112133.71	a
Unknown MIA (geissoschizine isomer 2)	94789.54 ± 12983.37	a	99653.36 ± 6191.92	a	82520.02 ± 12669.74	a	224065.05 ± 15165.13	b
Unknown 1 (isositsirikine isomer)	633621.94 ± 71704.80	a	633009.96 ± 27457.56	a	623066.31 ± 64597.28	a	658132.61 ± 43777.80	a
Isositsirikine	416370.09 ± 33899.53	a	394503.59 ± 19858.68	a	407085.93 ± 49096.73	a	540091.67 ± 29580.98	a
Perivine	143216.57 ± 6677.20	a	156338.91 ± 8522.00	ab	208846.44 ± 16544.12	b	301029.88 ± 17796.69	c
Akuammicine	243714.10 ± 14999.68	a	218826.92 ± 13563.25	a	275274.17 ± 32682.01	a	418342.16 ± 13918.86	b
O-acetylstemmadenine	256203.17 ± 9275.95	a	259941.53 ± 13593.20	a	239481.60 ± 12421.14	a	310548.87 ± 9331.95	b
16-hydroxytabersonine	204610.96 ± 6092.51	a	228663.65 ± 16080.62	a	213691.18 ± 22233.43	a	373824.82 ± 13240.55	b
16-hydroxytabersonine glucoside	12224.51 ± 3589.46	a	10193.54 ± 1107.57	a	7815.83 ± 768.46	a	25295.28 ± 2143.12	b
Catharanthine	9523886.58 ± 869603.54	a	9739259.01 ± 532742.66	a	10348636.85 ± 1029051.96	a	14733651.38 ± 570844.09	b
Unknown MIA (catharanthine isomer 1)	7153472.93 ± 456075.95	a	7302683.07 ± 500435.51	a	7760883.18 ± 763604.39	a	10508131.55 ± 751941.65	b
Unknown MIA (catharanthine isomer 2)	4440041.00 ± 323906.98	a	4409152.83 ± 302083.35	a	4809815.51 ± 470118.48	a	6787739.83 ± 427093.95	b
Unknown MIA (catharanthine isomer 3)	707826.50 ± 62826.83	a	722843.61 ± 48621.69	a	805858.79 ± 99615.49	a	1162660.09 ± 59521.33	b
Desacetoxyvindoline	761815.28 ± 44265.57	a	731145.46 ± 35310.95	a	728638.68 ± 43966.21	a	827125.88 ± 23511.19	a
Deacetylvindoline	349264.72 ± 26595.05	a	365332.19 ± 15360.14	a	363585.21 ± 37027.74	a	448500.41 ± 22624.85	a
Vindoline	10803870.27 ± 502242.35	a	10794229.84 ± 786105.05	a	9906909.68 ± 530268.23	a	10963867.33 ± 654783.22	a
Anhydrovinblastine	533421.25 ± 43732.49	a	336824.47 ± 41900.30	a	280294.74 ± 71595.24	a	482590.01 ± 78798.09	a
Vinblastine	119772.46 ± 10921.38	a	87389.33 ± 13087.25	a	61875.87 ± 16058.29	a	100642.06 ± 20718.21	a
Vincristine	112736.71 ± 8163.45	a	89011.88 ± 7574.91	a	83684.92 ± 15750.46	a	136641.02 ± 17197.14	a
Desacetoxyvindorosine	9109.75 ± 541.10	a	9099.32 ± 761.80	a	10478.56 ± 1119.57	a	10672.73 ± 149.04	a
Desacetylvindorosine	22891.62 ± 1651.31	a	24970.16 ± 1896.91	ab	33263.04 ± 3260.62	bc	36335.65 ± 1254.43	c
Demethoxyvindoline = vindorosine	623608.99 ± 60975.46	a	679723.22 ± 51393.01	ab	747367.33 ± 93818.53	ab	907585.89 ± 27750.51	b
Vincadifformine	3970.77 ± 906.62	a	4587.13 ± 557.40	a	4197.33 ± 611.45	a	8178.08 ± 466.37	b
16-Hydroxyvincadifformine	9319.24 ± 1475.38	a	10636.60 ± 716.42	a	10536.19 ± 1930.16	a	11137.91 ± 685.78	a
Minovincinine	2140.65 ± 168.68	a	1876.23 ± 244.38	a	2591.28 ± 419.49	a	4290.63 ± 301.01	b
19-hydroxytabersonine	43433.28 ± 2564.97	a	53601.84 ± 5291.97	a	53920.66 ± 4842.04	a	71636.23 ± 1804.03	b
19-O-acetyltabersonine	83064.17 ± 4142.76	a	91102.16 ± 5708.77	a	93903.91 ± 8907.29	a	138903.70 ± 7777.72	b

16-hydroxy-19-O-acetyltabersonine	287.33 ± 111.64	a	258.19 ± 96.52	a	380.19 ± 158.87	a	154.46 ± 41.51	a
16-hydroxylochnericine	239563.89 ± 24918.66	a	284786.38 ± 14148.97	ab	313035.09 ± 37059.05	ab	392134.46 ± 24480.09	b
16-hydroxylochnericine glucoside	11464.25 ± 8059.56	a	8321.25 ± 1827.04	a	7232.79 ± 1781.23	a	35186.17 ± 5258.00	b
Hörhammericine	37968.54 ± 2655.73	a	47364.79 ± 2702.62	ab	46619.19 ± 5077.90	a	60762.98 ± 2182.34	b
16-hydroxyhörhammericine	142.24 ± 25.16	a	70.10 ± 24.37	a	133.20 ± 16.90	a	124.92 ± 46.23	a
16-methoxyhörhammericine	9054.03 ± 114.64	a	8936.00 ± 402.63	a	8733.89 ± 1095.19	a	19182.45 ± 1781.51	b
Vandrikidine	154225.34 ± 14757.24	a	170333.52 ± 12008.57	a	178765.73 ± 21251.04	ab	232296.58 ± 5051.69	b

SUPPLEMENTARY TABLE S10 MIA levels upon overexpression of *MYB96/b, CrMYC2a*^{D126N} and *ORCA4*.

	p35S::GUS **± SEM**		***p35S::CrMYC2a***^{D126N} **± SEM**		***p35S::ORCA4*** **± SEM**		***p35S::MYB96*** **± SEM**		***p35S::CrMYC2a***^{D126N}***/ORCA4*** **± SEM**		***p35S::ORCA4/MYB96*** **± SEM**		***p35S::CrMYC2a***^{D126N}***/MYB96*** **± SEM**		***p35S::CrMYC2a***^{D126N}***/ORCA4/MYB96*** **± SEM**	
Loganin	9332.21 ± 670.24	a	9145.02 ± 1093.29	a	9749.58 ± 1349.63	a	11278.43 ± 1875.02	a	12390.85 ± 627.01	a	10389.86 ± 1093.90	a	8657.14 ± 629.17	a	11832.32 ± 496.03	a
Secologanin	80576.30 ± 1464.34	a	76038.85 ± 2902.62	a	76294.12 ± 2023.22	a	86004.92 ± 6046.99	a	82294.31 ± 2112.81	a	76911.70 ± 3463.54	a	81200.08 ± 5770.51	a	88012.94 ± 1482.64	a
Strictosidine	1878964.65 ± 32854.65	a	3681759.40 ± 390608.12	b	3589440.75 ± 367966.61	b	2118946.68 ± 340878.91	ac	5704397.26 ± 272573.10	d	3681482.17 ± 216910.28	b	3241250.30 ± 230225.64	bc	4298522.19 ± 82989.58	b
Strictosidine secologanoside	21.18 ± 21.18	a	478.54 ± 141.26	ac	155.79 ± 65.71	a	131.64 ± 89.13	a	3221.50 ± 467.63	b	266.15 ± 114.89	a	167.66 ± 166.01	a	1304.77 ± 296.92	c
Unknown 2 (strictosidine aglycone isomer)	80887.16 ± 2351.80	a	98933.16 ± 10800.57	a	80384.16 ± 6774.79	a	85617.32 ± 8941.26	a	109306.10 ± 2558.56	a	103188.24 ± 3899.31	a	92772.02 ± 5563.26	a	101132.05 ± 2185.94	a
Strictosidinic Acid	74892.04 ± 18046.67	a	151651.41 ± 18447.75	ad	448524.22 ± 53908.12	b	59766.04 ± 7006.73	a	730982.85 ± 41114.00	c	283137.79 ± 6982.35	d	178325.46 ± 20434.58	ad	480095.82 ± 38582.79	b
Serpentine	1773919.33 ± 179007.37	a	1797351.47 ± 355495.59	a	1411218.50 ± 242832.40	a	1945287.83 ± 312072.04	a	2116516.31 ± 107442.18	a	1944529.14 ± 212919.58	a	1680917.92 ± 254719.41	a	1922687.75 ± 168717.65	a
Unknown MIA (serpentine isomer)	543850.31 ± 37936.69	a	512655.45 ± 96116.74	a	479470.62 ± 79208.50	a	558725.21 ± 89192.89	a	781436.21 ± 29652.46	a	772071.87 ± 79957.80	a	521913.71 ± 62477.14	a	651391.30 ± 40765.34	a
Geissoschizine	935438.08 ± 50202.60	a	2704771.21 ± 400087.77	be	1912838.73 ± 261108.63	bc	1589684.30 ± 250103.99	c	4414049.70 ± 255513.88	d	1951477.70 ± 60394.70	abc	2001953.29 ± 66141.24	bc	3157425.18 ± 113417.51	e
Unknown MIA (geissoschizine isomer 1)	570123.83 ± 32701.96	a	1571415.11 ± 229194.25	bde	1175808.07 ± 176902.25	bd	958110.20 ± 141171.70	ad	2593610.43 ± 132045.21	c	1269091.18 ± 36405.24	d	1132908.32 ± 39506.84	ad	1878428.23 ± 76769.31	e
Unknown MIA (geissoschizine isomer 2)	61498.79 ± 8375.42	a	198383.12 ± 39106.71	bc	90652.22 ± 18901.02	ad	101180.94 ± 25685.36	acd	246594.52 ± 13297.48	b	104928.70 ± 19935.43	acd	203999.14 ± 20899.43	bd	187425.55 ± 20141.93	bd
Isositsirikine	195276.61 ± 2013.75	ab	289545.92 ± 32738.82	bd	246762.85 ± 21950.17	bd	217616.68 ± 30006.38	b	500285.21 ± 24540.06	c	306755.45 ± 14312.96	bd	263482.34 ± 15237.61	bd	337763.56 ± 7397.93	d
Unknown 1 (isositsirikine isomer)	304514.64 ± 6690.27	a	370906.49 ± 49861.56	a	311485.59 ± 30595.22	a	319953.69 ± 40361.99	a	528541.46 ± 12103.56	b	407747.41 ± 20396.93	ab	338506.06 ± 21948.50	a	408633.12 ± 9314.01	ab
Perivine	77500.61 ± 1958.16	a	329998.96 ± 57036.39	bcdf	99439.63 ± 9469.55	ad	114359.03 ± 21412.43	ad	303961.13 ± 20030.12	cdf	210373.57 ± 10500.41	d	496394.96 ± 48715.35	e	398180.07 ± 42894.45	ef
Akuammicine	137373.37 ± 5597.61	a	727458.69 ± 145196.32	bdef	253547.93 ± 32132.97	c	183977.91 ± 34245.82	c	968382.34 ± 42986.83	df	448383.84 ± 31297.80	ce	709936.97 ± 69036.30	ef	983034.25 ± 77078.21	f

O-acetylstemmadenine	113816.67 ± 9641.36	a	502984.15 ± 62387.95	bc	193477.32 ± 7164.20	a	133395.35 ± 17818.22	a	556439.36 ± 30591.19	b	233108.65 ± 8624.69	a	408625.87 ± 33884.72	c	481489.19 ± 11337.95	bc
16-hydroxytabersonine	80914.90 ± 5591.51	a	485874.77 ± 84793.03	bc	703212.60 ± 55317.69	c	112853.49 ± 22198.41	a	2379295.53 ± 122007.38	d	534157.38 ± 22011.97	bc	339230.59 ± 16354.39	ab	1567952.30 ± 81104.99	e
16-hydroxytabersonine glucoside	11654.71 ± 1357.93	a	48498.27 ± 8816.57	ab	80475.90 ± 8055.34	be	15251.98 ± 4438.25	a	233168.69 ± 13661.28	c	35502.78 ± 4289.39	a	45033.90 ± 5703.39	ae	151113.92 ± 17760.97	d
Catharanthine	6586547.17 ± 240876.72	ab	6637131.39 ± 1164743.46	ab	6432821.83 ± 637496.81	ab	7027293.38 ± 981397.24	ab	9873252.58 ± 309436.29	ab	10011238.24 ± 981359.59	a	6389266.73 ± 679522.44	b	8123683.85 ± 645254.12	ab
Unknown MIA (catharanthine isomer 1)	4483285.77 ± 143565.56	ab	4175576.27 ± 498917.24	a	4019400.89 ± 314794.82	a	4282055.57 ± 458618.14	a	5415114.71 ± 172585.81	ab	5919705.95 ± 431916.12	b	4165164.60 ± 318379.37	a	4690857.72 ± 249518.96	ab
Unknown MIA (catharanthine isomer 2)	3146655.64 ± 93617.46	ab	3054348.24 ± 429748.00	ab	2777286.75 ± 282517.61	a	2949961.39 ± 359451.51	a	4019998.50 ± 112737.07	ab	4234888.43 ± 264612.26	b	3370115.46 ± 283195.76	ab	3626089.34 ± 121601.08	ab
Unknown MIA (catharanthine isomer 3)	589747.11 ± 24632.93	ab	517141.25 ± 67525.09	a	539712.11 ± 56473.30	ab	544340.56 ± 68291.73	ab	754205.43 ± 24179.39	bc	779315.73 ± 46399.93	c	526403.13 ± 49781.83	a	629164.36 ± 33344.37	ac
Desacetoxyvindoline	572496.85 ± 36787.88	a	676426.49 ± 108263.61	a	652723.69 ± 65240.11	a	730985.35 ± 118851.25	a	881040.14 ± 34219.94	a	716408.76 ± 72773.19	a	649538.89 ± 83286.48	a	881291.02 ± 36764.44	a
Deacetylvindoline	237696.83 ± 8784.70	a	209199.29 ± 24728.42	a	203152.92 ± 19678.85	a	253281.45 ± 38531.45	a	233532.30 ± 10075.73	a	269212.29 ± 20301.13	a	259884.80 ± 16518.21	a	255929.47 ± 11027.13	a
Vindoline	9062435.33 ± 413759.41	a	8875880.17 ± 1103042.00	a	8949539.10 ± 856662.39	a	9892297.95 ± 1319574.54	a	11190508.80 ± 498438.71	a	11137011.40 ± 1017832.04	a	9415932.01 ± 896999.74	a	10612136.84 ± 460549.93	a
Anhydrovinblastine	294946.45 ± 7367.57	a	330214.63 ± 107480.02	a	228997.91 ± 61666.26	a	291103.18 ± 71641.33	a	498324.27 ± 30687.94	a	352055.39 ± 84950.86	a	277617.52 ± 78475.24	a	281310.18 ± 35430.55	a
Vinblastine	196068.75 ± 14170.45	a	235935.28 ± 75339.01	a	145213.82 ± 36744.45	a	216357.71 ± 47476.90	a	339491.03 ± 28535.21	a	254708.50 ± 54303.09	a	160679.39 ± 28666.33	a	211517.27 ± 35913.34	a
Vincristine	48430.87 ± 1888.95	a	62219.03 ± 12082.38	ab	57187.14 ± 11583.32	ab	77487.66 ± 11752.22	ab	90556.22 ± 3607.04	b	73501.85 ± 6853.79	ab	65165.31 ± 5437.77	ab	74491.45 ± 7056.38	ab
Desacetoxyvindorosine	6826.85 ± 874.77	a	8079.31 ± 1429.56	a	8734.79 ± 899.74	a	10657.55 ± 1794.77	abc	15681.26 ± 1180.10	bc	10292.56 ± 776.07	ac	8070.30 ± 1287.38	a	14655.16 ± 681.84	c
Desacetylvindorosine	14049.80 ± 591.97	a	12911.79 ± 1346.70	a	11032.18 ± 1359.84	a	14699.43 ± 1910.78	a	13289.98 ± 167.77	a	14396.46 ± 652.41	a	15390.35 ± 1103.33	a	15997.52 ± 700.07	a
Demethoxyvindoline = vindorosine	445804.30 ± 30556.19	a	410520.96 ± 77696.18	a	403909.35 ± 50553.81	a	455758.62 ± 59245.16	a	586173.39 ± 37190.59	a	561708.00 ± 64070.61	a	416085.62 ± 63025.83	a	526572.38 ± 49473.15	a
Vincadifformine	426.83 ± 141.27	a	3294.07 ± 936.88	a	1609.49 ± 379.28	a	367.00 ± 118.28	a	161717.94 ± 26586.34	b	1824.37 ± 481.91	a	3350.49 ± 1811.31	a	44784.72 ± 3955.88	a
16-Hydroxyvincadifformine	2523.05 ± 306.05	a	9292.96 ± 2029.49	a	4864.29 ± 806.95	a	4310.45 ± 852.99	a	171954.20 ± 16131.49	b	6652.05 ± 392.58	a	7063.49 ± 490.41	a	69848.73 ± 6492.19	c
Minovincinine	494.47 ± 111.91	ac	612.41 ± 183.35	ac	641.74 ± 167.28	ac	391.15 ± 60.25	ac	9238.83 ± 845.70	b	1098.05 ± 126.28	ac	378.30 ± 54.67	a	1892.15 ± 102.36	c
19-hydroxytabersonine	3202.36 ± 331.52	a	2959.23 ± 657.84	a	2635.34 ± 324.46	a	2573.33 ± 446.13	a	102841.04 ± 15335.70	b	4951.32 ± 717.16	a	2535.97 ± 315.08	a	39611.25 ± 5961.26	c
19-O-acetyltabersonine	52096.51 ± 432.48	a	163178.45 ± 19004.81	b	123485.41 ± 9155.91	ab	66025.44 ± 9462.93	a	516658.30 ± 37899.16	c	148414.08 ± 5677.54	b	145557.18 ± 9373.27	b	397300.75 ± 7568.17	d
16-hydroxy-19-O-acetyltabersonine	37.19 ± 14.46	a	46.29 ± 26.90	a	18.59 ± 18.01	a	59.04 ± 14.46	a	713.54 ± 103.70	b	38.19 ± 16.85	a	35.81 ± 25.18	a	99.71 ± 34.43	a
16-hydroxylochnericine	60875.77 ± 2312.96	a	118921.38 ± 17685.54	a	347874.15 ± 40717.38	b	66726.99 ± 9524.43	a	620212.14 ± 16731.42	c	403158.09 ± 10763.91	b	119378.89 ± 7251.55	a	546424.58 ± 37269.60	c

16-hydroxylochnericine glucoside	9703.16 ± 1610.01	a	20070.68 ± 4179.77	a	126704.48 ± 13047.73	b	13469.81 ± 4451.24	a	145039.15 ± 9232.74	b	83588.78 ± 5371.03	c	30701.25 ± 5026.19	a	114799.31 ± 16200.68	bc
Hörhammericine	5649.26 ± 488.75	a	5168.97 ± 1037.97	a	4944.01 ± 793.23	a	4522.21 ± 628.76	a	160223.61 ± 16721.94	b	7298.05 ± 414.32	a	5552.77 ± 844.45	a	112375.38 ± 18507.65	c
16-hydroxyhörhammericine	205.76 ± 16.17	a	94.76 ± 16.01	a	151.71 ± 41.33	a	106.23 ± 35.07	a	12716.05 ± 833.07	b	248.22 ± 30.72	a	128.68 ± 44.84	a	9024.61 ± 1475.29	c
16-methoxyhörhammericine	5669.46 ± 299.63	a	8416.25 ± 1018.94	a	8027.10 ± 969.87	a	5994.55 ± 1319.74	a	1278211.75 ± 148975.94	b	14673.34 ± 856.02	a	14074.94 ± 2002.04	a	600138.97 ± 80691.00	c
Vandrikidine	66372.97 ± 3644.32	a	59197.58 ± 10499.71	a	59401.41 ± 5904.84	a	64826.75 ± 9454.28	a	293123.07 ± 28000.55	b	94476.22 ± 4949.51	a	58706.73 ± 5826.50	a	138841.73 ± 8077.63	c

References

Bailey, T. L., Johnson, J., Grant, C. E., and Noble, W. S. (2015). The MEME Suite. *Nucleic Acids Res.* 43**,** W39-W49. doi:10.1093/nar/gkv416

Buchan, D. W. A., and Jones, D. T. (2019). The PSIPRED Protein Analysis Workbench: 20 years on. *Nucleic Acids Res.* 47**,** W402-W407. doi:10.1093/nar/gkz297

Disfani, F. M., Hsu, W.-L., Mizianty, M. J., Oldfield, C. J., Xue, B., Dunker, A. K., et al. (2012). MoRFpred, a computational tool for sequence-based prediction and characterization of short disorder-to-order transitioning binding regions in proteins. *Bioinformatics* 28**,** i75-i83. doi:10.1093/bioinformatics/bts209

Dugé De Bernonville, T., Carqueijeiro, I., Lanoue, A., Lafontaine, F., Sánchez Bel, P., Liesecke, F., et al. (2017). Folivory elicits a strong defense reaction in *Catharanthus roseus*: metabolomic and transcriptomic analyses reveal distinct local and systemic responses. *Sci. Rep.* 7**,** 40453. doi:10.1038/srep40453

Gongora-Castillo, E., Childs, K. L., Fedewa, G., Hamilton, J. P., Liscombe, D. K., Magallanes-Lundback, M., et al. (2012). Development of transcriptomic resources for interrogating the biosynthesis of monoterpene indole alkaloids in medicinal plant species. *PLoS ONE* 7**,** e52506. doi:10.1371/journal.pone.0052506

O'shea, C., Staby, L., Bendsen, S. K., Tidemand, F. G., Redsted, A., Willemoës, M., et al. (2017). Structures and short linear motif of disordered transcription factor regions provide clues to the interactome of the cellular hub protein radical-induced cell death1. *J. Biol. Chem.* 292**,** 512-527. doi:10.1074/jbc.M116.753426

Pan, Y.-J., Lin, Y.-C., Yu, B.-F., Zu, Y.-G., Yu, F., and Tang, Z.-H. (2018). Transcriptomics comparison reveals the diversity of ethylene and methyl-jasmonate in roles of TIA metabolism in *Catharanthus roseus*. *BMC Genomics* 19**,** 508. doi:10.1186/s12864-018-4879-3

Van Moerkercke, A., Fabris, M., Pollier, J., Baart, G. J., Rombauts, S., Hasnain, G., et al. (2013). CathaCyc, a metabolic pathway database built from Catharanthus roseus RNA-Seq data. *Plant Cell Physiol* 54**,** 673-685. doi:10.1093/pcp/pct039

Van Moerkercke, A., Steensma, P., Schweizer, F., Pollier, J., Gariboldi, I., Payne, R., et al. (2015). The bHLH transcription factor BIS1 controls the iridoid branch of the monoterpenoid indole alkaloid pathway in *Catharanthus roseus*. *Proc. Natl. Acad. Sci. USA* 112**,** 8130-8135. doi:10.1073/pnas.1504951112

Xue, B., Dunbrack, R. L., Williams, R. W., Dunker, A. K., and Uversky, V. N. (2010). PONDR-FIT: a meta-predictor of intrinsically disordered amino acids. *Biochim. Biophys. Acta - Proteins Proteomics* 1804**,** 996-1010. doi:10.1016/j.bbapap.2010.01.011
